# An intramolecular C–N cross-coupling of β-enaminones: a simple and efficient way to precursors of some alkaloids of *Galipea officinalis*

**DOI:** 10.3762/bjoc.11.99

**Published:** 2015-05-27

**Authors:** Hana Doušová, Radim Horák, Zdeňka Růžičková, Petr Šimůnek

**Affiliations:** 1 Institute of Organic Chemistry and Technology, Faculty of Chemical Technology, University of Pardubice, Studentská 573, CZ 532 10, Pardubice, Czech Republic; 2 Department of General and Inorganic Chemistry, Faculty of Chemical Technology, University of Pardubice, Studentská 573, CZ 532 10, Pardubice, Czech Republic

**Keywords:** C–N cross-coupling, copper, enaminone, palladium, tetrahydroquinoline

## Abstract

2-Aroylmethylidene-1,2,3,4-tetrahydroquinolines with the appropriate substituents can be suitable precursors for the synthesis of alkaloids from *Galipea officinalis* (cuspareine, galipeine, galipinine, angustureine). However, only two, rather low-yielding procedures for their synthesis are described in the literature. We have developed a simple and efficient protocol for an intramolecular, palladium or copper-catalysed amination of both chloro- and bromo-substituted 3-amino-1,5-diphenylpent-2-en-1-ones leading to the above-mentioned tetrahydroquinoline moiety. The methodology is superior to the methods published to date.

## Introduction

**Figure 1:**
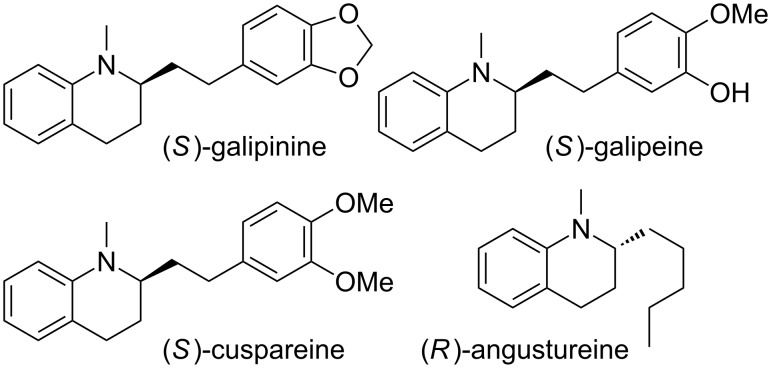
Tetrahydroquinoline alkaloids of *Galipea officinalis*.

*Galipea officinalis* Hancock is a Venezuelan shrub, the bark (angostura bark) of which is used in folk medicine for stimulation in the cure of some paralytic diseases [1] and for the treatment of fever [2]. In addition, antituberculous [3], antiplasmodial and cytotoxic [2] properties have been also described. The active constituents of the bark are mainly tetrahydroquinoline alkaloids such as galipinine, galipeine, cuspareine and angustureine (Figure 1) [1,4].

**Scheme 1:**
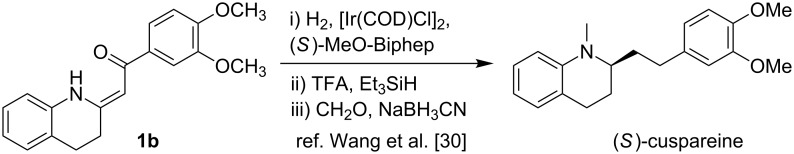
Enaminone-based synthesis of *(S)*-cuspareine.

The synthesis of these alkaloids has attracted great interest in organic chemists and many procedures have been published to date [5–30]. As part of our ongoing interest in the preparation of polarized ethylenes and their application in organic synthesis, we have been attracted by the procedure published by Zhou [30]. Here, the authors used heterocyclic enaminone **1b** as the reactant for an asymmetric reduction followed by *N*-methylation to give (*S*)-cuspareine in high yields and excellent stereoselectivity (Scheme 1).

**Scheme 2:**
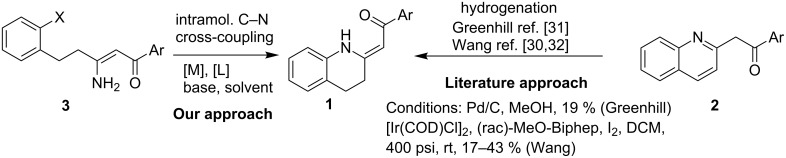
The approaches to 2-aroylmethylidene-1,2,3,4-tetrahydroquinolines **1**.

This method, however, suffers from a low-yielding (28%) synthesis of the enaminone **1a** [30]. Only two methods for the synthesis of tetrahydroquinolines **1** have been described in the literature [30–32] based on a catalytic hydrogenation of the corresponding quinolones **2** (Scheme 2) with yields not greater than 43%. In the present work, we introduce a quite different approach to the exocyclic enaminones **1** based on an intramolecular C–N cross-coupling of enaminones **3** (Scheme 2).

## Results and Discussion

### The synthesis of the starting substrates

**Scheme 3:**
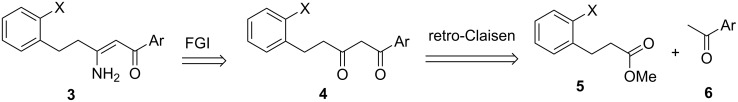
The retrosynthetic analysis of the starting substrates for C–N cross-coupling.

A simple retrosynthesis of enaminones **3** leads to the corresponding β-diketones **4** accessible through Claisen condensation of 3-phenylpropionic ester **5** with the appropriate acetophenone **6** (Scheme 3).

**Scheme 4:**
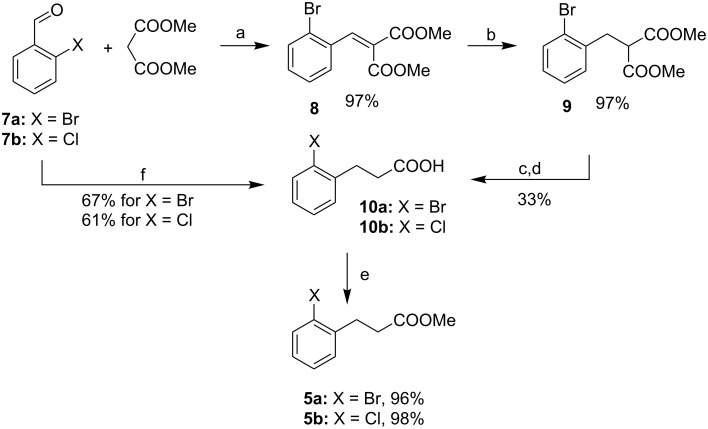
The synthesis of methyl 3-phenylpropionates. Conditions: (a) piperidine, PhCOOH, toluene, reflux 4 h; (b) NaBH_4_, MeOH/MeCN, rt, 3.5 h; (c) KOH, H_2_O, reflux, 8 h; (d) H_2_SO_4_, H_2_O, reflux, 20 h; (e) MeOH, SOCl_2_, reflux, 4 h; (f) Meldum’s acid, HCOOH, Et_3_N, 100 °C, 4 h.

The synthesis of ester **5** was accomplished according to Scheme 4. The classic Knoevenagel/reduction/hydrolysis/decarboxylation pathway (steps a–d in Scheme 4) to carboxylic acid **10** suffered from low yields of the last two steps (total yield 33%). The attempt to obtain **5a** directly from **9** by means of Krapcho decarboxylation [33] failed, as the product was contaminated with inseparable byproducts. A better and less time-consuming route to **10** consists of the reaction of 2-halobenzaldehyde **7** with Meldrum´s acid in the presence of HCOOH/Et_3_N system with a total yield of 61–67% (step f in Scheme 4). The last step for both methods was the esterification of acids **10a,b** (step e in Scheme 4).

**Scheme 5:**
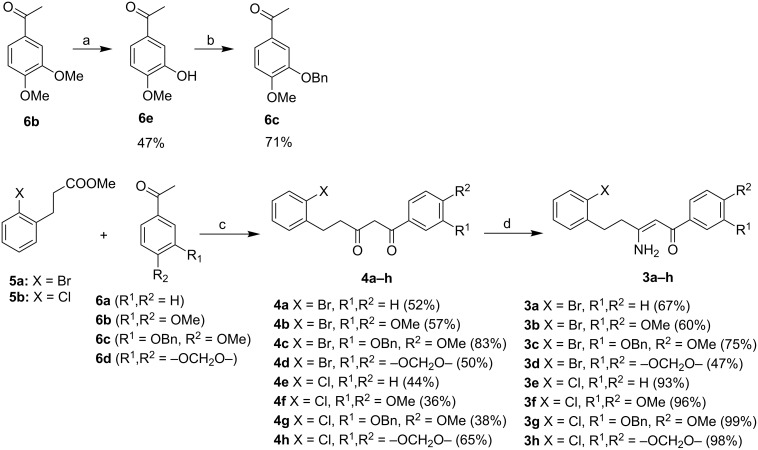
The synthesis of the starting β-enaminones. Conditions: (a) H_2_SO_4_, 65 °C, 46 h; (b) 1. *t-*BuOK/THF, rt, 30 min, 2. BnBr, reflux 5 h; (c) *t-*BuOK, *t-*BuONa or *t-*AmOK, THF, rt overnight; (d) AcONH_4_, MeOH, reflux 5 h (NH_4_HCO_3_, MeOH/THF, rt 24 h for **3d**).

β-Diketones **4** were obtained using *tert*-butoxide or *tert*-pentoxide mediated Claisen condensation of esters **5** with the appropriate acetophenones **6a–d** (Scheme 5, step c). The substitution pattern on compounds **6** was chosen so that the final products **1** are the precursors for the synthesis of galipinine, galipeine and angustureine (Figure 1). 3-Hydroxy-4-methoxyacetophenone (**6e**) was prepared by selective deprotection of commercially available 3,4-dimethoxyacetophenone (**6b**) [34] (Scheme 5, step a). The hydroxy group in **6e** was then protected with a benzyl group (step b). The final step d was the reaction of β-diketones **4** with ammonium surrogate (AcONH_4_ or NH_4_HCO_3_). The regioselectivity of the synthesis was checked by means of 2D ^1^H–^13^C HMBC (See Supporting Information File 1, page S69). The non-equivalence of NH_2_ protons together with the relatively high chemical shift of one resonance of the pair (δ ≈ 10 ppm) indicates the presence of an intramolecular hydrogen bond N–H···O. The enaminones **3** therefore possess *Z* configuration on the C=C bond.

### The intramolecular C–N cross-coupling

β-Enaminones and related polarized ethylenes (generally enamines substituted by EWG on β-carbon atom) belong among the rather neglected molecules from a C–N cross-coupling perspective. There are relatively few works (about ten) dealing with these very useful molecules [35–46], in comparison to the hundreds of papers dedicated to the other substrates. Due to their electronic nature, β-enaminones can be considered vinylogous amides. Hence, their nucleophilicity is lowered and enaminones can be more challenging substrates for C–N cross-coupling compared to ordinary enamines.

We used **3a** as the model substrate for the optimization study. The reaction conditions were surveyed from the following aspects: catalytic system [M]/[L] and base (Table 1).

**Table 1:** Optimisation study for C–N cross-coupling of bromo derivatives.

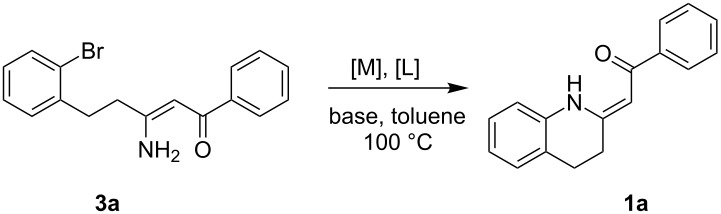					

Entry	[M]/%	[L]/%^a^	Base/equiv	Time/h	Conversion^b^

1^c^	Pd_2_(dba)_3_/2.5	**L1**/5	Cs_2_CO_3_/1.6	3	87
2^c^	Pd_2_(dba)_3_/2.5	**L1**/5	Cs_2_CO_3_/1.6	18	87
3^c^	Pd_2_(dba)_3_/3.5	**L1**/7	Cs_2_CO_3_/1.4	2	94 (92)
4^c^	Pd_2_(dba)_3_/3.5	**L5**/7	Cs_2_CO_3_/1.4	17	96 (88)
5^d^	Pd(OAc)_2_/5	**L1**/15	Cs_2_CO_3_/1.4	21	0
6^e^	CuI/10	**L7**/20	Cs_2_CO_3_/1.4	18	0
7^e^	CuI/10	**L8**/20	Cs_2_CO_3_/1.4	18	>99 (83)
8^e^	CuI/10	**L8**/20	K_3_PO_4_/2	20	12
9^e^	CuI/10	**L8**/20	K_2_CO_3_/2	20	0

^a^For ligands see Figure 2. ^b^Conversion determined by means of ^1^H NMR. Isolated yields in parentheses. ^c^Method A (See Experimental and Supporting Information File 1). ^d^Method B. Water-mediated pre-activation used [47]. (See Supporting Information File 1). ^e^Method C. (See Supporting Information File 1).

**Figure 2:**
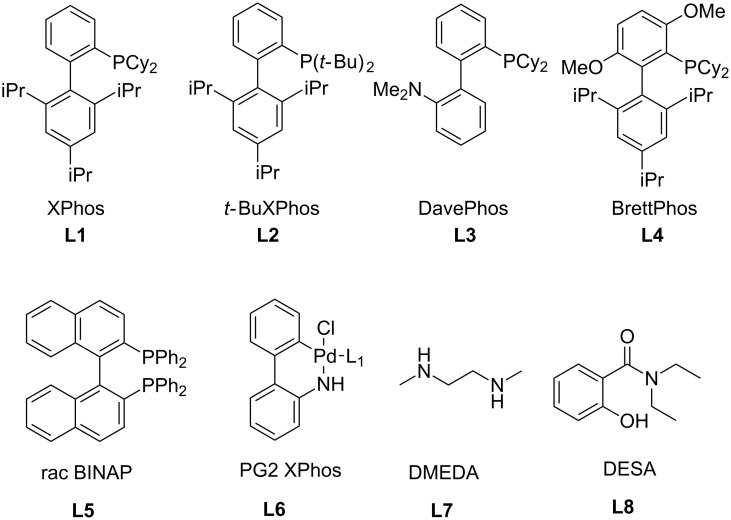
Ligands for C–N cross-coupling used in this work.

We used tris(dibenzylideneacetone)dipalladium(0) as the starting palladium source. To suppress the problems with the attenuation of its catalytic activity due to the coordination of dba ligands to the central metal, we applied preheating of Pd_2_(dba)_3_ with the corresponding ligand [L] to generate the active catalyst [48] prior to the addition to substrate **3** (see Method A in the Experimental). Dialkylbiarylphosphines currently belong among the most common ligands for palladium-catalyzed C–N cross-coupling [48–49]. The use of **L1** (Figure 2) in combination with Pd_2_(dba)_3_ in toluene led to the successful formation of product **1a** (Table 1, entries 1–3). Rather, a higher amount of palladium was necessary for completing the reaction in a short reaction time (Table 1, entry 3). Switching to the bidentate ligand **L5** (Figure 2) gave comparable conversion but over a substantially longer period (Table 1, entry 4). Buchwald et al. [47] described a protocol for generation of the highly active Pd(0) catalyst from Pd(OAc)_2_ using water-mediated pre-activation. However, no conversion was observed here for **L1** (Table 1, entry 5).

Besides palladium, copper is another widely applied metal for C–N bond formation [50–52]. We then applied the CuI/[L] catalytic system to **3a** (see method C in Supporting Information File 1, pages S25 and S26). The choice of the ligand is crucial here, as **L7** (Figure 2) had no effect (Table 1, entry 6) whereas using another common ligand **L8** (Figure 2) led to the full conversion to **1a** (Table 1, entry 7). Caesium carbonate was the optimal base as neither K_2_CO_3_ nor K_3_PO_4_ gave satisfactory results (Table 1, entries 8 and 9).

From the above-mentioned facts, summarized in Table 1, it follows that the optimal results were obtained using catalytic systems Pd_2_(dba)_3_/**L1** (Table 1, entry 3) or CuI/**L8** (Table 1, entry 7) in toluene with caesium carbonate as the base. We further preferred the first one (Table 1, entry 3) both due to the lower amount of the catalyst and shorter reaction time. Nevertheless, the copper-mediated variant could be interesting for preparations on a larger scale.

With the conditions chosen for the bromo derivatives, we turned our attention to the chloro derivatives. As chloro derivatives are more challenging substrates for cross-coupling reactions, we started with the successful catalytic system (Table 1, entry 3) but with an increased amount of palladium, applied to **3e** as the model substrate. However these conditions failed (Table 2, entry 1). Changing the ligand to DavePhos (**L3**) or BINAP (**L5**) (Figure 2) did not improve the situation at all (Table 2, entries 2 and 3). The application of a palladacycle-based pre-catalyst **L6** (Figure 2) (see method D in Supporting Information File 1, pages S25 and S26), introduced by Buchwald et al. [53] failed as well (Table 2, entries 4–6). The sterically more demanding ligand BrettPhos (**L4**, Figure 2), reported as a very effective ligand for *N*-arylations of primary amino groups [48,54], worked better but was still not satisfactory (Table 2, entry 7). The breakthrough was made after applying *t-*BuXPhos (**L2**, Figure 2), which led to 64% conversion (Table 2, entry 8). Switching from toluene to *t-*AmOH finally led to full conversion to the desired product (Table 2, entry 9). An attempt to reduce the amount of catalyst, however, only led to a decrease in the conversion (Table 2, entry 10). The copper-catalyzed protocol was quite unsuccessful (Table 2, entry 11). Generally, chloro derivatives (and especially non-activated ones) remain challenging substrates for copper catalysis [50]. The conditions in Table 2, entry 9 (5 mol % Pd_2_(dba)_3_, 10 mol % *t-*BuXPhos, Cs_2_CO_3_ in *t-*AmOH) were thus the best for the cyclization of chloro derivatives.

**Table 2:** Optimisation study for C–N cross-coupling of chloro derivatives.

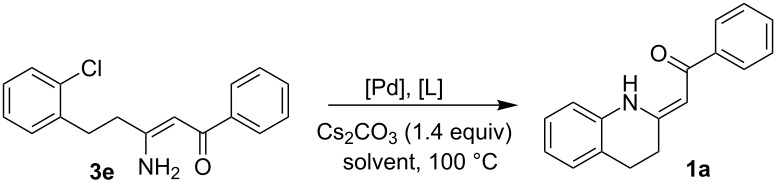					

Entry	[Pd]/%	[L]/%^a^	Solvent	Time/h	Conversion/%^b^

1^c^	Pd_2_(dba)_3_/5	**L1**/10	toluene	48	0
2^c^	Pd_2_(dba)_3_/5	**L3**/10	toluene	24	0
3^c^	Pd_2_(dba)_3_/5	**L5**/10	toluene	48	0
4^d,e^	**L6**/3		toluene	24	0
5^d,e^	**L6**/3		DMF	24	4
6^d,e^	**L6**/3		*t-*AmOH	24	0
7^c^	Pd_2_(dba)_3_/5	**L4**/10	toluene	22	29
8^c^	Pd_2_(dba)_3_/5	**L2**/10	toluene	22	64
9^c^	Pd_2_(dba)_3_/5	**L2**/10	*t-*AmOH	17	100 (72)^f^
10^c^	Pd_2_(dba)_3_/2.5	**L5**/5	*t-*AmOH	24	47
11^g^	CuI/10	**L7**/20	toluene	24	0

^a^For ligands see Figure 2. ^b^Conversion determined by means of ^1^H NMR. Isolated yields in parentheses. ^c^Method A. ^d^Method D. ^e^Two equivalents of the base used. ^f^Isolated yield. ^g^Method C.

These conditions, however, did not work for the bromo derivatives, despite the higher amount of both palladium and ligand and a substantially longer reaction time. Compound **3b** was transformed into **1b** using the above-mentioned conditions (Table 2, entry 9) only in 20% conversion.

Having the conditions for the successful cyclization of enaminones **3a,e** to tetrahydroquinoline **1a** in hand, we applied them to other enaminones **3**. In all of these cases, the corresponding tetrahydroquinolines **1** were obtained in moderate to high yields (Table 3).

**Table 3:** The intramolecular C–N cross-coupling of enaminones **3**: a summary of results.

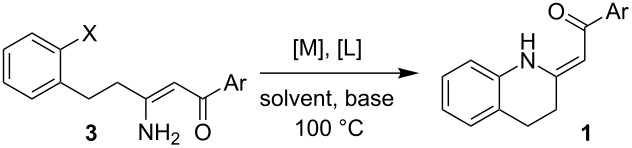						

Product^a^	Reactant	General procedure^b^	Solvent	[L]	Base	Yield[%]/Conv.[%]

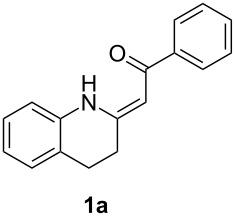 (p. S27)	**3a**	A C	toluene toluene	**L1** **L8**	Cs_2_CO_3_ Cs_2_CO_3_	92 83
	**3e**	A^c^	*t-*AmOH	**L2**	Cs_2_CO_3_	72
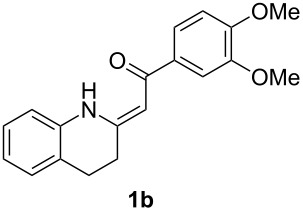 (p. S28)	**3b**	A A A B	toluene toluene toluene toluene	**L1** **L1** **L1** **L1**	Cs_2_CO_3_ K_2_CO_3_ K_3_PO_4_ Cs_2_CO_3_	65 /29 /0 /0
	**3f**	A	*t-*AmOH	**L2**	Cs_2_CO_3_	72
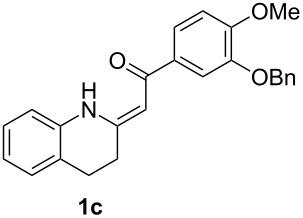 (p. S29)	**3c**	A^d^	toluene	**L1**	Cs_2_CO_3_	85/50^e^
	**3g**	A	*t-*AmOH	**L2**	Cs_2_CO_3_	82
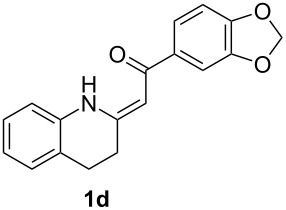 (p. S30)	**3d**	A	toluene	**L1**	Cs_2_CO_3_	45
	**3h**	A	*t-*AmOH	**L2**	Cs_2_CO_3_	61

^a^See Supporting Information File 1 for details. ^b^For procedures see Supporting Information File 1 p. S25 (methods A,B) or p. S26 (methods C,D). ^c^For 2.5 mol % Pd_2_(dba)_3_, 5 mol % **L2** conversion 47%. ^d^5 mol % of Pd_2_(dba)_3_ and 10 mol % **L1** used. ^e^Conversion for 3.5 mol % of Pd_2_(dba)_3_ and 7 mol % **L1**.

**Figure 3:**
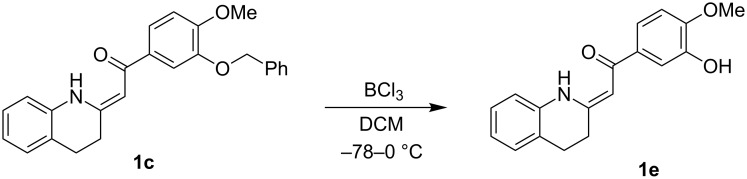
Deprotection of the hydroxy group in **1c** to give the Galipein precursor **1e**.

The deprotection of the hydroxy group in **1c** was accomplished using BCl_3_ in DCM (Figure 3) to obtain the precursor **1e** for galipeine (Figure 1).

**Figure 4:**
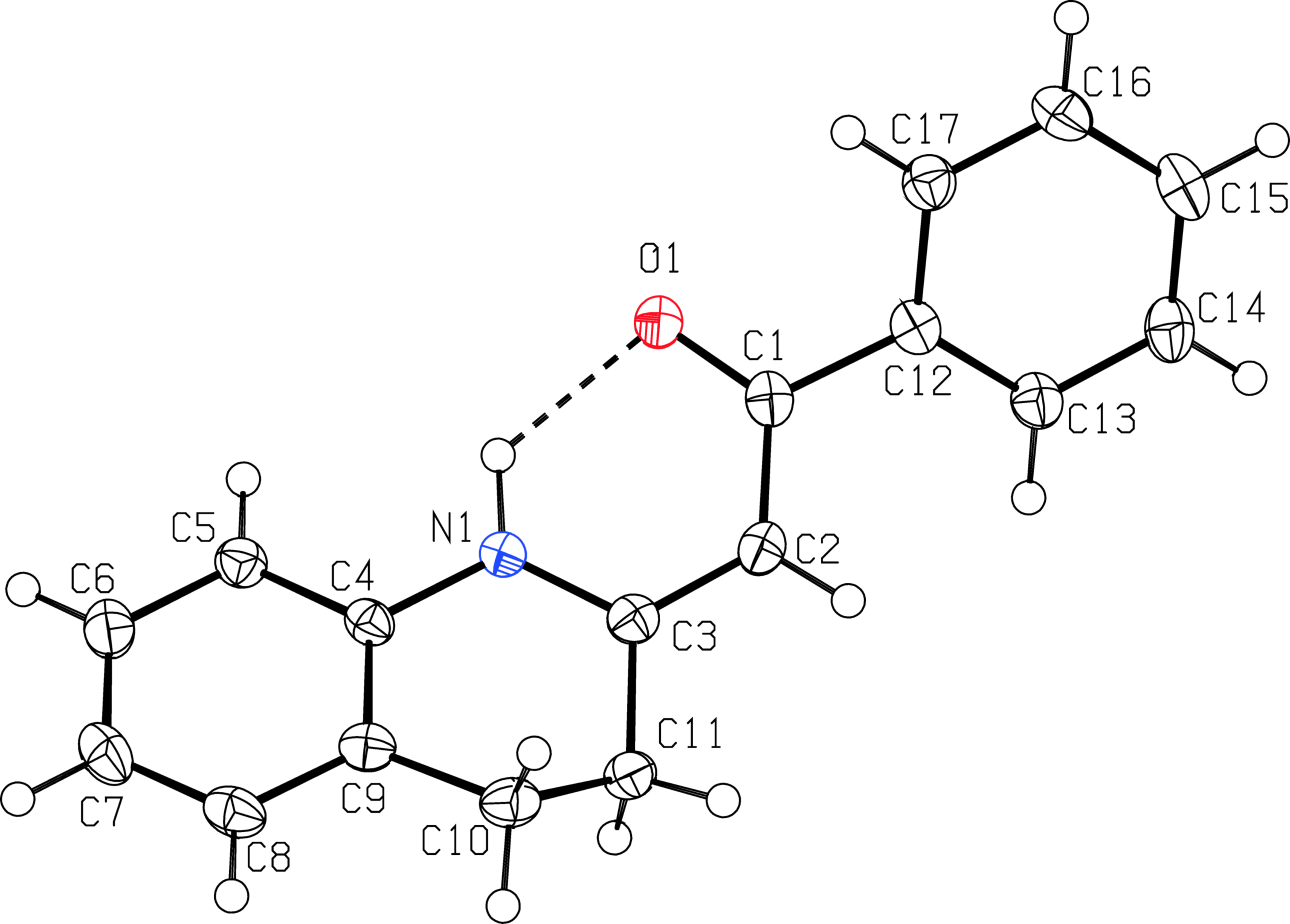
ORTEP (50% probability level) view for compound **1a**. For selected parameters see Supporting Information File 1.

On the basis of relatively high chemical shifts of NH protons in compounds **1** (δ > 12), it can be assumed that all of these compounds have *Z* configuration on the C=C double bond (increased chemical shifts due to the presence of an intramolecular hydrogen bond C=O···H–N). This assumption was confirmed by means of X-ray characterization of the compound **1a** (Figure 4).

According to the literature [55], as well as the Cambridge Structural Database, there is a plethora of compounds with an intramolecular N–H···O=C contact like **1a**; on the other hand, the family of structurally related 1,2-dihydroquinolines [56] and ethanones [57] is limited to only seven examples. In the structure of **1a**, some extent of π-electron delocalization is reflected in slight shortening of the formal single bond between C1 and C2 atoms, on the contrary to a slight elongation of C2–C3 and C1–O1 distances – formally the double bonds (for crystal data see Supporting Information File 1, pages S27 and S28), similarly to the situation found for peptide type of bonding [55].

## Conclusion

An asymmetric reduction of suitably substituted 2-aroylmethylidene-1,2,3,4-tetrahydroquinolines is one of the possible routes to tetrahydroquinoline alkaloids of *Galipea officinalis*. The methodology, however, suffered from the unsatisfactory source of the 2-aroylmethylidene reactants. In this work, we have established a novel, simple protocol for the synthesis of the above-mentioned 2-aroylmethylidene-1,2,3,4-tetrahydroquinolines, which is superior to the methods published so far. The methodology is based on an intramolecular C–N cross-coupling of acyclic β-enaminones. The reaction conditions are described for the successful cyclization of both bromo and chloro derivatives. The crucial factors here are the ligand and the solvent. The best system for bromo derivatives is Pd_2_(dba)_3_/XPhos/Cs_2_CO_3_ in toluene, although the transformation is also feasible using CuI/DESA/Cs_2_CO_3_ in toluene, albeit for a substantially longer time than in the palladium-catalyzed version. The more challenging chloro derivatives required higher amounts of palladium, different ligands and solvents, together with much longer reaction times. Pd_2_(dba)_3_/*t-*BuXPhos/Cs_2_CO_3_ in *t-*AmOH worked best. Due to the importance of polarized ethylenes, the extension of the methodology to other substrates and substituents could be useful and is the subject of thorough examination nowadays.

## Experimental

For analytical and synthetic procedures as well as characterization data of individual compounds and copies of their NMR spectra see Supporting Information File 1.

### Representative procedure for palladium-catalyzed synthesis of tetrahydroquinolines **1a–d** (Method A)

An oven-dried vial equipped with a magnetic stir bar and fitted with a Teflon septum was charged with Pd_2_(dba)_3_ and the corresponding ligand. The vessel was evacuated three times and backfilled with argon. Subsequently, the solvent (3 mL) was added via a syringe and the mixture was preheated at 100 °C for 30 min. Another oven-dried vial was charged with Cs_2_CO_3_ and substrate **3**. Also, this vessel was evacuated three times and backfilled with argon. The solution of the activated catalyst was transferred from the first vial into the second one via a syringe. The vessel was then heated at 100 °C until the starting component was fully consumed (control by TLC). The mixture was then diluted with EtOAc and filtered through a small plug of Celite^®^ S which was subsequently thoroughly washed with EtOAc. The filtrate was concentrated in vacuo and the residue was purified by recrystallization or column chromatography (see details at individual compounds).

**1-Phenyl-2-((2*Z*)-1,2,3,4-tetrahydroquinolin-2-ylidene)ethan-1-one (1a):** Method A: from **3a**, 3.5 mol % Pd_2_(dba)_3_, 7 mol % XPhos, toluene, 2 h. Column chromatography (silica gel; DCM/EtOAc 10:1). Yield 92%. From **3e**, 5 mol % Pd_2_(dba)_3_, 10 mol % *t-*BuXPhos, *t*-AmOH, 17 h. Column chromatography (silica gel; DCM). Yield 72%. Yellow solid, mp 103–105 °C (ref. [31] reports 105–106 °C). Proton NMR data are in accordance with [32]. ^1^H NMR (400.13 MHz) δ 12.85 (brs, 1H), 7.94–7.91 (m, 2H), 7.50–7.42 (m, 3H), 7.21–7.17 (m, 1H), 7.12–7.10 (m, 1H), 6.99–6.94 (m, 2H), 5.88 (s, 1H), 2.89–2.86 (m, 2H), 2.75–2.71 (m, 2H) ppm; ^13^C NMR (100.62 MHz) δ 189.7, 159.0, 139.9, 136.7, 131.3, 128.5, 128.4, 127.9, 127.3, 125.3, 123.3, 116.8, 92.6, 28.8, 24.4 ppm.

## Supporting Information

File 1 Experimental procedures, characterization data and copies of NMR spectra.
