# Supplementary material for: An intramolecular C–N cross-coupling of β-enaminones: a simple and efficient way to precursors of some alkaloids of Galipea officinalis
Source: Beilstein J Org Chem. 2015 May 27;11:884–92. doi: 10.3762/bjoc.11.99 (PMC4464440; doi:10.3762/bjoc.11.99)
Supplement: File 1 — Experimental procedures, characterization data and copies of NMR spectra. [file Beilstein_J_Org_Chem-11-884-s001.pdf]

Supporting Information

for

An Intramolecular C–N cross-coupling of β-enaminones: a simple and efficient way to precursors of some alkaloids of *Galipea officinalis*

Hana Doušová[1], Radim Horák[1], Zdeňka Růžičková[2] and Petr Šimůnek[*1]

Address: [1]Institute of Organic Chemistry and Technology, Faculty of Chemical Technology, University of Pardubice, Studentská 573, CZ 532 10, Pardubice, Czech Republic and [2]Department of General and Inorganic Chemistry, Faculty of Chemical Technology, University of Pardubice, Studentská 573, CZ 532 10, Pardubice, Czech Republic

Email: Petr Šimůnek[*] - petr.simunek@upce.cz

*Corresponding author

Experimental

General

All the solvents and reagents were commercial and used without further purification. Anhydrous solvents were stored under argon with using Sure/Seal™ or AcroSeal™. Ligands, bases and transition metal sources were stored under argon in a desiccator.

NMR spectra were measured in $CDCl_3$ using either a Bruker AVANCE III 400 spectrometer operating at 400.13 MHz (^{1}H) and 100.62 MHz (^{13}C) or a Bruker Ascend™ 500 machine operating at 500.20 MHz (^{1}H) and 125.79 MHz (^{13}C). All the pulse sequences were taken from the Bruker software library. Proton spectra were calibrated on internal TMS (δ = 0.00) and carbon spectra on the middle signal of the solvent multiplet (δ = 77.23). Carbon spectra were measured with broadband proton decoupling either in normal way or by means of an APT pulse sequence.

Elemental analyses were performed on a Flash EA 2000 CHNS automatic analyzer (Thermo Fisher Scientific). MALDI–HRMS were measured using a MALDI LTQ Orbitrap XL (Thermo Fisher Scientific) with DHB (2,5-dihydroxybenzoic acid) as the matrix.

Melting points were measured on a Kofler hot-stage microscope Boetius PHMK 80/2644 and were not corrected.

The X-ray data for yellow crystals of **1a** were obtained at 150 K using an Oxford Cryostream low-temperature device on a Nonius Kappa CCD diffractometer with Mo K_{α} radiation (λ = 0.71073 Å), a graphite monochromator, and the ϕ and χ scan mode. Data reductions were performed with DENZO-SMN [1]. The absorption was corrected by integration methods [2]. Structures were solved by direct methods (Sir92) [3] and refined by full matrix least-square based on F^2 (SHELXL97) [4]. Hydrogen atoms were mostly

localized on a difference Fourier map, however to ensure uniformity of treatment of crystal, all hydrogen atoms were recalculated into idealized positions (riding model) and assigned temperature factors H_{iso}(H) = 1.2 U_{eq} (pivot atom) or of 1.5U_{eq} (methyl). H atoms in methylene, vinylidene moieties and hydrogen atoms in aromatic rings were placed with C–H distances of 0.96 Å and 0.93 Å, and 0.86 Å for N–H bond.

$R_{int} = \sum|F_o^2 - F_{o,mean}^2|/\sum F_o^2$, GOF = $[\sum(w(F_o^2 - F_c^2)^2)/(N_{diffrs} - N_{params})]^{1/2}$ for all data, $R(F) = \sum||F_o| - |F_c||/\sum|F_o|$ for observed data, $wR(F^2) = [\sum(w(F_o^2 - F_c^2)^2)/(\sum w(F_o^2)^2)]^{1/2}$ for all data.

Crystallographic data for structural analysis have been deposited with the Cambridge Crystallographic Data Centre, CCDC no. 1042186 for **1a**. Copies of this information may be obtained free of charge from the Director, CCDC, 12 Union Road, Cambridge CB2 1EY, UK (fax: +44-1223-336033; e-mail: deposit@ccdc.cam.ac.uk or www: http:// www.ccdc.cam.ac.uk).

Experimental procedures and characterization data

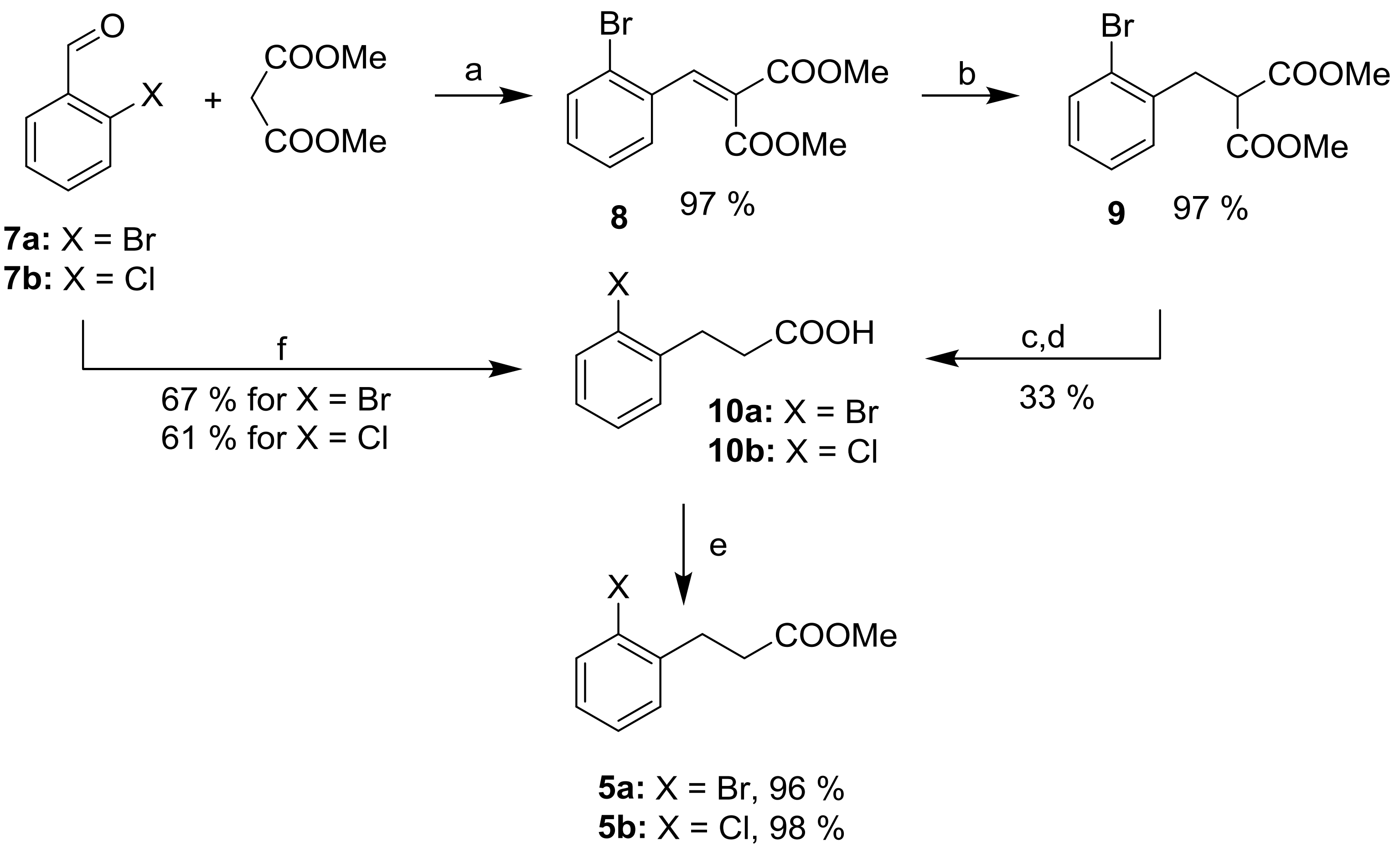

Conditions: (a) piperidine, PhCOOH, toluene, reflux 4h; (b) $NaBH_4$, MeOH/MeCN, rt, 3.5 h; (c) KOH, H_2O, reflux, 8 h; (d) H_2SO_4, H_2O, reflux 20 h; (e) MeOH, $SOCl_2$, reflux 4 h; (f) Meldum's acid, HCOOH, Et_3N, 100°C, 4 h.

Dimethyl 2-bromobenzylidenemalonate (8): The compound was prepared according to the procedure adopted from ref.[5] A 250 mL round-bottom flask was charged with 2-bromobenzaldehyde (20.5 g, 111 mmol), dimethyl malonate (15.35 g, 116 mmol) and piperidine (1.64 g, 19 mmol) followed with benzoic acid (1.5 g, 12 mmol) and toluene (120 mL). The mixture was then heated and water formed during the reaction was removed by azeotropic distillation (4 h). Toluene was then distilled off and the residue was dissolved in EtOAc (100 mL), washed with water (40 mL), HCl (1.4M, 2 × 40 mL), saturated aqueous $NaHCO_3$ (2 × 40 mL) and brine (40 mL), dried over anhydrous sodium sulphate and evaporated *in vacuo* to give 32.4 g of red oil (97%). The crude product is, according to NMR, pure enough to be used in the next reaction step. ^{1}H NMR Data were in accordance with the literature [6].

^{1}H NMR (400.13 MHz): δ = 8.01 (s, 1H), 7.63 (dd, *J* = 1.4 Hz, 7.8 Hz, 1H), 7.38 (dd, *J* = 1.8 Hz, 7.6 Hz, 1H), 7.30 (td, *J* = 1.3 Hz, 7.5 Hz, 1H), 7.27–7.23 (m, 1H), 3.88 (s, 3H), 3.74 (s, 3H) ppm.

Dimethyl 2-bromobenzylmalonate (9): A 500 mL round-bottom flask was charged with substrate **8** (32.4 g, 108 mmol) dissolved in 1:1 v/v MeOH/MeCN mixture (250 mL). Sodium borohydride (6.15 g, 162 mmol) was carefully portion-wise added into the solution under cooling by cold water during one hour. The reaction mixture was further stirred at laboratory temperature for 3.5 h, then quenched by pouring on saturated aqueous ammonium chloride (450 mL) and extracted by DCM (3 × 200 mL). The organic phase was dried over anhydrous sodium sulphate and evaporated *in vacuo* to give a red oil (31.5 g, 97%) sufficiently pure (according to NMR) to be subjected to the next reaction step without any purification. Proton NMR data were in accordance with

literature [7]. ***^{1}H NMR (400.13 MHz):*** δ = 7.55–7.53 (m, 1H), 7.25–7.19 (m, 2H), 7.12–7.08 (m, 1H), 3.89 (t, *J* = 7.8 Hz, 1H), 3.70 (s, 6H), 3.34 (d, *J* = 7.8 Hz, 2H,) ppm.

3-(2-Bromophenyl)propanoic acid (10a): ***Method A:*** A mixture of crude diester **9** (31.5 g, 105 mmol) and KOH (11.8 g, 210 mmol) in H_2O (180 mL) was refluxed for 8 h. After cooling and washing with DCM (3 × 80 mL), the aqueous layer was separated, diluted with H_2O (63 mL) and acidified by conc. H_2SO_4 (21 mL, 393 mmol). The mixture was refluxed for 20 h and cooled in an ice bath. The crude solid was separated by suction, dried and recrystallized from petroleum ether/chloroform mixture giving 7.8 g of white solid (33% yield) with mp 96–100 °C (ref.[8] reports 99–100 °C). Proton NMR spectrum is in accordance with ref.[8]

Method B: A three-necked flask fitted with a dropping funnel, reflux condenser and thermometer was charged with 85% formic acid (8 mL, 180 mmol). After cooling the solution to 5 °C, triethylamine (2.7 mL, 27 mmol) was added dropwise maintaining the temperature below 10 °C. Subsequently, 2-bromobenzaldehyde (**7a**, 5 g, 27 mmol) and Meldrum´s acid (3.9 g, 27 mmol) were added to the solution and the mixture was refluxed for 4 h. Afterwards the mixture was cooled to an ambient temperature and poured onto ice-cold water (30 mL). The resulting suspension was acidified by 5.5M HCl until pH ≈ 1 and stored in a refrigerator overnight. The precipitated crystals were filtered with suction, washed with water (3 × 20 mL), dried in desiccator and dissolved in chloroform (100 mL). Undissolved impurities were filtered off. The filtrate was concentrated to the volume of about 10 mL and petroleum ether (15 mL) was added under stirring and heating the solution. After gradual cooling a white solid was formed. Yield 4.17 g (67%) of solid with mp 93–95 °C (ref. [8] reports 99–100 °C). Proton NMR spectrum is in accordance with ref.[8]

^{1}H NMR (400.13 MHz): δ = 11.14 (brs, 1H), 7.55–7.53 (m, 1H), 7.28–7.22 (m, 2H), 7.12–7.07 (m, 1H), 3.10–3.06 (m, 2H), 2.74–2.70 (m, 2H) ppm.

3-(2-Chlorophenyl)propanoic acid (10b): This compound was prepared from 2-chlorobenzaldehyde (**7b**) in the same manner as **10a** (Method B). Yield 61%, mp 90–91 °C (ref. [9] reports 94–96 °C). Proton NMR spectrum is consistent with that in ref. [9]

^{1}H NMR (400.13 MHz): δ = 7.37–7.34 (m, 1H), 7.27–7.25 (m, 1H), 7.22–7.15 (m, 2H), 3.09–3.05 (m, 2H), 2.73–2.69 (m, 2H) ppm.

Methyl 3-(2-bromophenyl)propanoate (5a): To a solution of acid **10a** (7.7 g, 33.6 mmol) in methanol (60 mL) stirred at ambient temperature thionyl chloride (0.9 mL, 12 mmol) was added dropwise. The mixture was refluxed for 1 h. Subsequently another thionyl chloride was added in two portions (2 × 0.9 mL, 24 mmol) under reflux, the second portion was added 1 h after the first one. After next two hours of reflux, the volatile components were distilled off under reduced pressure (70 °C, 0.8 kPa). The light yellow oily residue was dissolved in DCM (60 mL) and washed with saturated aqueous $NaHCO_3$ (60 mL). The organic layer was dried over anhydrous sodium sulfate, filtered, and concentrated under reduced pressure to give yellow oily liquid (7.8 g, 96%). Proton NMR data are consistent with literature [10].

^{1}H NMR (400.13 MHz): δ = 7.52–7.50 (m, 1H), 7.25–7.19 (m, 2H), 7.08–7.03 (m, 1H), 3.66 (s, 3H), 3.07–3.04 (m, 2H), 2.66–2.62 (m, 2H) ppm.

Methyl 3-(2-chlorophenyl)propanoate (5b): This compound was prepared from acid **10b** using the same procedure as for the synthesis of **5a**. Yield of yellow oily liquid was 98%. NMR data are in accordance with those reported in ref. [9]

^{1}H NMR (500.20 MHz): δ = 7.34 (dd, *J* = 7.6 Hz, 1.6 Hz, 1H), 7.25–7.24 (m, 1H), 7.20–7.14 (m, 2H), 3.67 (s, 3H), 3.08–3.05 (m, 2H), 2.67–2.64 (m, 2H) ppm.

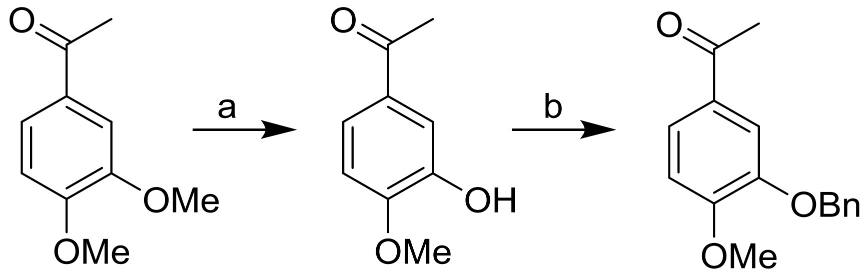

6b **6e** **6c**

47 % 71 %

Conditions: (a) H_2SO_4, 65 °C, 46 h; (b) 1. *t*-BuOK/THF, rt, 30 min, 2. BnBr, reflux 5 h;

3-Hydroxy-4-methoxyacetophenone (6e): A solution of ketone **6b** (12.5 g, 69.5 mmol) in concentrated H_2SO_4 (62.5 mL) was stirred at 65 °C for 46 h. After cooling the reaction mixture to ambient temperature, it was poured onto ice (250 g) and stirred for 1 h. The precipitate was filtered off, washed with water and redissolved in 1M aqueous NaOH (16 mL, 160 mmol). The mixture was extracted with DCM (65 mL). The aqueous layer was acidified with concentrated HCl (25 mL), stirred for 1.5 h and the precipitate was filtered off to give 5.39 g (47%) of the phenol as brown solid with mp 80–81 °C (lit. [11] reports 88–90 °C). ^{1}H NMR data are in accordance with those published in ref.[11].

^{1}H NMR (400.13 MHz): δ = 7.57–7.54 (m, 2H), 6.91–6.88 (m, 1H), 5.73 (brs, 1H), 3.97 (s, 3H), 2.55 (s, 3H) ppm.

3-Benzyloxy-4-methoxyacetophenone (6c): A three-necked flask (250 mL) fitted with a dropping funnel and reflux condenser was charged with *t*-BuOK (1.69 g, 15 mmol). The apparatus was evacuated and backfilled with argon three-times. Subsequently, the base was suspended in anhydrous THF (80 mL). After homogenization of the mixture by intensive stirring, the solution of **6e** (2.5 g, 15 mmol) in anhydrous THF (20 mL) was added dropwise during 10 min. The mixture was then stirred for 30 min. at room temperature. Afterwards, the solution of benzyl bromide (2.57 g, 15 mmol) in anhydrous THF (30 mL) was added dropwise during 15 min. The mixture was refluxed for 5 h and then let stir overnight under argon atmosphere. Subsequently, water (100 mL) was added into the reaction mixture, the organic layer was separated and the aqueous layer was extracted with diethyl ether (3 × 50 mL). The organic layers were combined, dried over anhydrous sodium sulfate and concentrated under reduced pressure to give the crude product which was purified by column chromatography (silica gel; DCM). Yield: 2.72 g (71%); mp 68–70 °C (ref. [12] reports 75–76 °C). Proton NMR data are consistent with those reported in ref.[12]

^{1}H NMR (400.13 MHz): δ = 7.60–7.58 (m, 2H), 7.48–7.46 (m, 2H), 7.40–7.36 (m, 2H), 7.33–7.29 (m, 1H), 6.91 (d, *J* = 8.5 Hz, 1H), 5.19 (s, 2H), 3.94 (s, 3H), 2.53 (s, 3H) ppm.

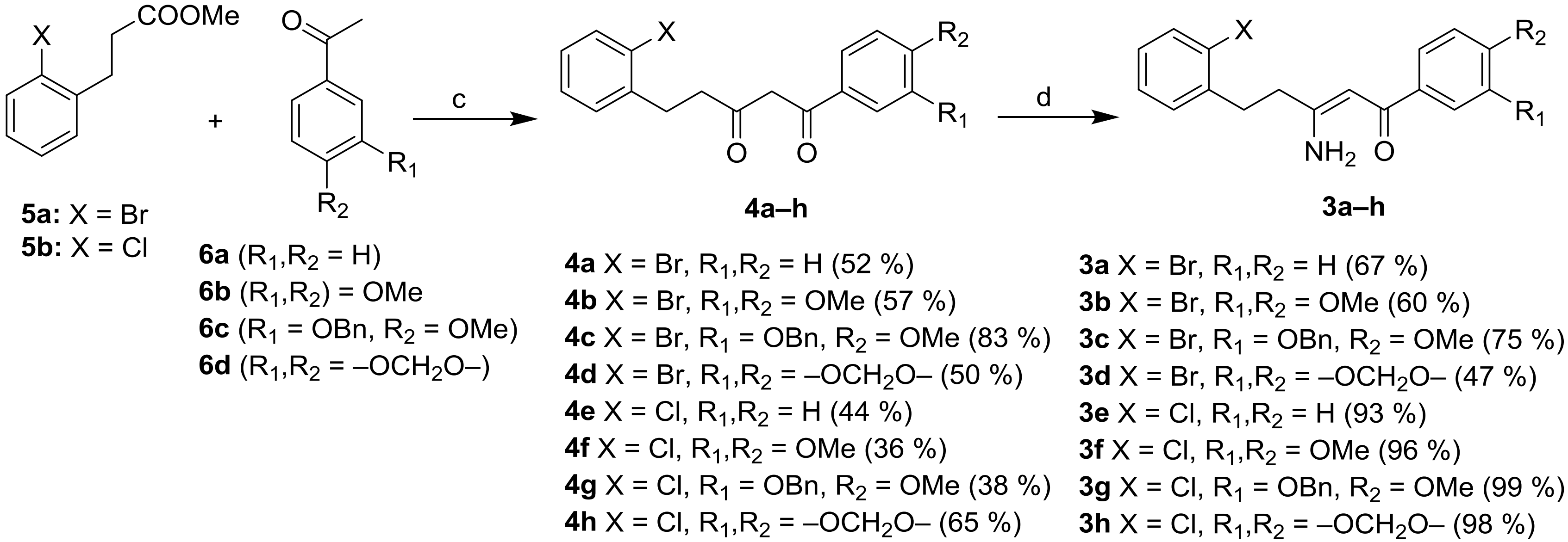

5a: X = Br
5b: X = Cl

6a (R_1,R_2 = H)
6b (R_1,R_2) = OMe
6c (R_1 = OBn, R_2 = OMe)
6d (R_1,R_2 = –OCH_2O–)

4a–h

4a X = Br, R_1,R_2 = H (52 %)
4b X = Br, R_1,R_2 = OMe (57 %)
4c X = Br, R_1 = OBn, R_2 = OMe (83 %)
4d X = Br, R_1,R_2 = –OCH_2O– (50 %)
4e X = Cl, R_1,R_2 = H (44 %)
4f X = Cl, R_1,R_2 = OMe (36 %)
4g X = Cl, R_1 = OBn, R_2 = OMe (38 %)
4h X = Cl, R_1,R_2 = –OCH_2O– (65 %)

3a–h

3a X = Br, R_1,R_2 = H (67 %)
3b X = Br, R_1,R_2 = OMe (60 %)
3c X = Br, R_1 = OBn, R_2 = OMe (75 %)
3d X = Br, R_1,R_2 = –OCH_2O– (47 %)
3e X = Cl, R_1,R_2 = H (93 %)
3f X = Cl, R_1,R_2 = OMe (96 %)
3g X = Cl, R_1 = OBn, R_2 = OMe (99 %)
3h X = Cl, R_1,R_2 = –OCH_2O– (98 %)

(c) *t*-BuOK, *t*-BuONa or *t*-AmOK, THF, rt overnight; (d) $AcONH_4$, MeOH, reflux 5 h (NH_4HCO_3, MeOH/THF, rt 24 h for **3d**).

General procedure for the synthesis of β-diketones 4a–h

A three-necked flask (100 mL) fitted with a dropping funnel and reflux condenser was charged with 1.1–2 equivalents of the base (see details at individual compounds). The apparatus was evacuated and backfilled with argon three-times. Subsequently, the base was suspended in anhydrous THF (20 mL). In the case of using a toluene solution of *t*-AmOK as the base the empty apparatus was 3× evacuated and backfilled with argon and the solution of the base was thereafter added via syringe. After homogenization of the mixture by intensive stirring (in the cases of using solid *t*-BuONa or *t*-BuOK, resp.), the solution of the ketone **6** (8.5 mmol) in

anhydrous THF (8 mL) was added dropwise during 10 min. The mixture was then stirred for 30 min. at room temperature. Afterwards, the solution of the ester **5** (8.5 mmol) in anhydrous THF (8 mL) was added dropwise during 15 min. The reaction mixture was then stirred over 1–2 nights (see details at individual compounds) at room temperature under argon atmosphere. Subsequently, saturated aqueous NH_4Cl (15–40 mL, depending on the amount of the base) was added into the reaction mixture, the organic layer was separated and the aqueous layer was extracted with EtOAc (3 × 35 mL). The organic layers were combined, washed with saturated aqueous $NaHCO_3$ (2 × 35 mL) and brine (1 × 35 mL) and dried over anhydrous sodium sulfate. The volatile components were evaporated under reduced pressure. The residue was subjected to purification either through Cu^{2+} complex (the procedure published in [13] was adopted, for details *vide infra*) or chromatography or recrystallization (see details at individual compounds).

Purification of crude β-diketones through copper-diketonate complex

To a solution of crude β-diketone (9 mmol) in EtOH (3.6 mL), the solution of $CuCl_2 \cdot 2H_2O$ (0.77 g, 4.5 mmol) in EtOH (7.7 mL) was added. A solution of NaOH (0.36 g, 9 mmol) in water (2 mL) was added dropwise. After 30 min. of stirring the pH of the solution was 6–7 and the green-gray precipitate of Cu^{2+} complex was formed, filtered off, washed three-times with EtOH and dried on the air. The dried complex was dissolved in diluted (1:9) H_2SO_4 (7 mL) and DCM (5 mL) and stirred for 1.5 h at 60 °C. The mixture was cooled down and extracted with DCM (3 × 20 mL). The combined organic layers were dried over Na_2SO_4 and the solvent was evaporated under reduced pressure.

The following compounds were prepared using the above-mentioned procedure:

5-(2-Bromophenyl)-1-phenylpentane-1,3-dione (4a): 1.2 equiv *t*-BuONa, reaction time overnight, purification through copper diketonate, yield 52% of red oil. Product is 9:1 mixture of keto-enol tautomers.

^{1}H NMR (400.13 MHz): **Enol form** δ = 16.1 (s, 1H), 7.87–7.84 (m, 2H), 7.56–7.53 (m, 1H), 7.52–7.50 (m, 1H), 7.47–7.42 (m, 2H), 7.28–7.21 (m, 2H), 7.10–7.06 (m, 1H), 6.15 (s, 1H), 3.15–3.11 (m, 2H), 2.78–2.75 (m, 2H). **Keto form** δ = 7.92–7.90 (m, 2H), 7.61–7.57 (m, 1H), 4.10 (s, 2H), 3.06–3.01 (m, 2H), 2.96–2.92 (m, 2H) ppm. Other signals are overlapped by the signals of the major enol form.

^{13}C NMR (100.62 MHz): δ = 195.7, 182.9, 139.9, 134.7, 132.9, 132.3, 130.5, 128.6, 128.1, 127.6, 127.0, 124.3, 96.3, 39.2, 32.1 ppm. Only the signals of the enol form.

Elemental analysis: for $C_{17}H_{15}BrO_2$ (331.2): calcd. C 61.65, H 4.56; found: C 61.77, H 4.51.

5-(2-Bromophenyl)-1-(3,4-dimethoxyphenyl)pentane-1,3-dione (4b): 1.2 equiv *t*-BuONa, reaction time overnight, purification through copper diketonate, yield 57% of yellow solid, mp 81–85 °C. Product is 12:1 mixture of keto-enol tautomers.

^{1}H NMR (400.13 MHz): **Enol form** δ = 16.28 (brs, 1H), 7.55 (dd, *J* = 8.0 Hz, 1.1 Hz, 2H), 7.47 (dd, *J* = 8.4 Hz, 2.0 Hz, 1H), 7.44 (d, *J* = 2 Hz, 1H), 7.29–7.17 (m, 2H), 7.10–7.06 (m, 1H), 6.89 (d, *J* = 8,5 Hz, 1H), 6.09 (s, 1H), 3.95 (s, 3H), 3.94 (s, 3H), 3.15–3.11 (m, 2H), 2.75–2.72 (m, 2H). **Keto form** δ = 7.53–7.50 (m, 2H), 6.88 (d, *J* = 8.4 Hz, 1H), 4.06 (s, 2H), 3.95 (s, 3H), 3.93 (s, 3H), 3.05–3.00 (m, 2H), 2.95–2.91 (m, 2H) ppm. Other signals are overlapped by the signals of the major enol form.

^{13}C NMR (100.62 MHz): δ = 192.7, 184.7, 153.0, 149.2, 140.1, 133.1, 130.7, 128.3, 128.0, 127.8, 124.5, 121.4, 110.6, 109.7, 95.7, 56.24, 56.19, 38.7, 32.5 ppm. Only the signals of the enol form.

Elemental analysis: for $C_{19}H_{19}BrO_4$ (391.3): calcd. C 58.33, H 4.89; found C 58.45, H 4.90.

5-(2-Bromophenyl)-1-(3-benzyloxy-4-methoxyphenyl)pentane-1,3-dione (4c): 1.5 equiv *t*-BuOK, reaction time two nights.

Purification: the product was precipitated from the residue by EtOH in an ultrasonic bath and isolated by suction. Another part of the product can be obtained by evaporation of ethanol and subjecting the residue to a column chromatography (silica gel DCM). Yield 83%, mp 95–96.5 °C. Product is 5:1 mixture of keto-enol tautomers.

^{1}H NMR (400.13 MHz): **Enol form** δ = 16.23 (brs, 1H), 7.56–7.54 (m, 1H), 7.50–7.45 (m, 4H), 7.41–7.31 (m, 4H), 7.25–7.23 (m, 1H), 7.10–7.06 (m, 1H), 6.92–6.88 (m, 1H), 6.02 (s, 1H), 5.19 (s, 2H), 3.94 (s, 3H), 3.13–3.09 (m, 2H), 2.73–2.69 (m, 2H). **Keto form** δ = 7.54–7.51 (m, 2H), 7.28–7.27 (m, 1H), 7.22–7.17 (m, 2H), 7.05–7.03 (m, 1H), 5.17 (s, 2H), 4.01 (s, 2H), 3.95 (s, 3H), 3.03–3.00 (m, 2H), 2.90–2.89 (m, 2H) ppm. Other signals are overlapped by the signals of the major enol form.

^{13}C NMR (100.62 MHz): δ = 192.6, 184.3, 153.4, 148.1, 139.9, 136.6, 132.9, 130.5, 128.6, 128.1, 127.6, 127.5, 124.3, 121.6, 112.2, 110.9, 95.5, 71.0, 56.1, 38.5, 32.3, 30.0, 29.7 ppm. Only the signals of the enol form.

Elemental analysis: for $C_{25}H_{23}BrO_4$ (467,352): calcd. C 64.25, H 4.96, Br 17.10; found C 64.50, H 4.98, Br 17.08.

HRMS *m/z* calculated for $C_{25}H_{24}BrO_4$ $[M + H]^+$ 467.08525, found 467.08540; for $C_{25}H_{23}BrNaO_4$ $[M + Na]^+$ 489.06719, found 489.06732; for $C_{25}H_{23}BrKO_4$ $[M + K]^+$ 505.04113, found 505.04124.

1-(1,3-Benzodioxol-5-yl)-5-(2-bromophenyl)pentane-1,3-dione (4d): 1.2 equiv *t*-BuONa, reaction time overnight, purification through column chromatography (silica gel, DCM), resulting oil solidified upon standing with cyclohexane. Yield 50%, mp 68–71 °C. Product is 7:1 mixture of keto-enol tautomers.

^{1}H NMR (400.13 MHz): **Enol form** δ = 16.21 (brs, 1H), 7.56–7.53 (m, 2H), 7.47–7.44 (m, 1H), 7.34–7.33 (d, *J* = 1.8 Hz, 1H), 7.28–7.21 (m, 2H), 6.84 (d, *J* = 8.2 Hz, 1H), 6.04 (s, 2H), 6.03 (s, 1H), 3.13–3.09 (m, 2H), 2.74–2.70 (m, 2H) ppm. **Keto form** δ 7.51–7.48 (m, 2H), 7.39 (d, *J* = 1.8 Hz, 1H), 6.84 (d, *J* = 8.2 Hz, 1H), 6.05 (s, 2H), 4.01 (s, 2H), 3.04–3.00 (m, 2H), 2.90–2.95 (m, 2H) ppm. Other signals are overlapped by the signals of the major enol form.

^{13}C NMR (100.62 MHz): δ = 193.1, 183.7, 151.3, 148.1, 139.9, 132.9, 130.5, 129.3, 128.0, 127.6, 124.3, 122.7, 108.2, 107.1, 101.8, 95.6, 38.6, 32.3 ppm. Only the signals of the enol form.

Elemental analysis: for $C_{18}H_{15}BrO_4$ (375.2): calcd. C 57.62, H 4.03; found C 57.43, H 3.95.

5-(2-Chlorophenyl)-1-phenylpentane-1,3-dione (4e): 2 equiv *t*-BuOK, reaction time two nights, purification through column chromatography (silica gel, DCM), yield 44%, oil. Product is 10:1 mixture of keto-enol tautomers.

^{1}H NMR (500.20 MHz): **Enol form** δ = 16.10 (brs, 1H), 7.86–7.85 (m, 2H), 7.53–7.50 (m, 1H), 7.46–7.43 (m, 2H), 7.37–7.35 (m, 1H), 7.28–7.26 (m, 1H), 7.20–7.15 (m, 2H), 6.15 (s, 1H), 3.14–3.11 (m, 2H), 2.78–2.75 (m, 2H) ppm. **Keto form** δ = 8.00–7.99 (m, 1H), 7.92–7.90 (m, 2H), 7.61–7.55 (m, 2H), 7.34–7.30 (m, 2H), 7.24–7.22 (m, 1H), 7.14–7.12 (m, 1H), 4.10 (s, 2H), 3.05–3.02 (m, 2H), 2.96–2.93 (m, 2H) ppm.

^{13}C NMR (125.79 MHz): δ = 196.0, 183.1, 138.4, 134.9, 134.1, 132.5, 130.7, 129.8, 128.8, 128.0, 127.18, 127.16, 96.5, 39.2, 29.8 ppm. Only the signals of the enol form.

Elemental analysis: for $C_{17}H_{15}ClO_2$ (286.75): calcd. C 71.20, H 5.27; found C 71.40, H 5.32.

5-(2-Chlorophenyl)-1-(3,4-dimethoxyphenyl)pentane-1,3-dione (4f): 1.1 equiv *t*-AmOK (1.7M solution in toluene), reaction time two nights, purification through copper diketonate, yield 36%, oil. Product is 5:1 mixture of keto-enol tautomers.

^{1}H NMR (500.20 MHz): **Enol form** δ = 16.29 (s, 1H), 7.48–7.46 (m, 1H), 7.45 (d, *J* = 2 Hz, 1H), 7.37–7.35 (m, 1H), 7.28–7.26 (m, 1H), 7.21–7.16 (m, 2H), 6.89 (d, *J* = 8,5 Hz, 1H), 6.09 (s, 1H), 3.95 (s, 3H), 3.94 (s, 3H), 3.14–3.11 (m, 2H), 2.75–2.72 (m, 2H) ppm. **Keto form** δ = 7.53–7.50 (m, 2H), 7.33–7.30 (m, 1H), 7.23–7.21 (m, 1H), 7.15–7.11 (m, 3H), 4.06 (s, 2H), 3.95 (s, 3H), 3.93 (s, 3H), 3.04–3.01 (m, 2H), 2.95–2.92 (m, 2H) ppm.

^{13}C NMR (100.62 MHz): δ = 192.5, 184.5, 152.7, 148.9, 138.2, 134.1 130.5, 129.5, 128.0, 127.97, 127.1, 121.3, 110.5, 109.7, 95.7, 56.19, 56.14, 38.3, 29.8 ppm. Only the signals of the enol form.

Elemental analysis: for $C_{19}H_{19}ClO_4$ (346.8): calcd. C 65.80, H 5.52; found C 65.95, H 5.54.

HRMS *m/z* calculated for $C_{19}H_{20}ClO_4$ $[M + H]^+$ 347.10446, found 347.10481; for $C_{19}H_{19}ClNaO_4$ $[M + Na]^+$ 369.08641, found 369.08681; for $C_{19}H_{19}ClKO_4$ $[M + K]^+$ 385.06035, found 385.06078.

5-(2-Chlorophenyl)-1-(3-benzyloxy-4-methoxyphenyl)pentane-1,3-dione (4g): 2 equiv *t*-BuOK, reaction time two nights, purification through column chromatography (silica gel, DCM), yield 38%, white solid, mp 102–103 °C. Product is 5:1 mixture of keto-enol tautomers.

^{1}H NMR (400.13 MHz): **Enol form** δ = 16.23 (brs, 1H), 7.50–7.45 (m, 4H), 7.38–7.31 (m, 4H), 7.27–7.25 (m, 1H), 7.19–7.14 (m, 2H), 6.92–6.89 (m, 1H), 6.01 (s, 1H), 5.19 (s, 2H), 3.93 (s, 3H), 3.13–3.09 (m, 2H), 2.73–2.69 (m, 2H) ppm. **Keto form** δ = 7.54–7.51 (m, 3H), 7.40 (m, 3H), 7.30 (m, 1H), 7.31–7.20 (m, 2H), 7.14–7.12 (m, 1H), 5.17 (s, 2H), 4.00 (s, 2H), 3.94 (s, 3H), 3.03–2.99 (m, 2H), 2.92–2.88 (m, 2H) ppm. Other signals are overlapped by the signals of the major enol form.

^{13}C NMR (100.62 MHz): δ = 192.6, 184.3, 153.4, 148.1, 138.2, 136.6, 133.9, 130.5, 129.5, 128.6, 128.1, 127.8, 127.5, 126.9, 121.6, 112.2, 110.9, 95.4, 71.1, 56.1, 38.3, 29.8 ppm. Only the signals of the enol form.

Elemental analysis: for $C_{25}H_{23}ClO_4$ (422.9): calcd. C 71.00, H 5.48, Cl 8.38; found C 71.24, H 5.52, Cl 8.32.

1-(1,3-Benzodioxol-5-yl)-5-(2-chlorophenyl)pentane-1,3-dione (4h): 1.1 equiv *t*-AmOK (1.7M solution in toluene), reaction time two nights, purification through column chromatography (silica gel, DCM), yield 65%, red solid, mp 77–78 °C. Product is 6:1 mixture of keto-enol tautomers.

^{1}H NMR (400.13 MHz): **Enol form** δ = 16.22 (brs, 1H), 7.45–7.43 (m, 1H), 7.38–7.30 (m, 2H), 7.26–7.22 (m, 1H), 7.20–7.12 (m, 2H), 6.83 (d, *J* = 8.3 Hz, 1H), 6.02 (s, 2H), 6.03 (s, 1H), 3.12–3.08 (m, 2H), 2.73–2.69 (m, 2H) ppm. **Keto form** δ = 7.49–7.47 (m, 1H), 6.04 (s, 2H), 4.00 (s, 2H), 3.03–3.00 (m, 2H), 2.93–2.89 (m, 2H) ppm.

^{13}C NMR (100.62 MHz): δ = 193.4, 184.0, 151.5, 148.3, 138.4, 134.1, 130.7, 129.7, 129.5, 128.0, 127.1, 122.9, 108.4, 107.3, 102.0, 95.7, 38.6, 29.9 ppm. Only the signals of the enol form.

Elemental analysis: for $C_{18}H_{15}ClO_4$ (330.8): calcd. C 65.36, H 4.57, Cl 10.72; found C 65.28, H 4.55, Cl 10.61.

HRMS *m/z* calculated for $C_{18}H_{16}ClO_4$ $[M + H]^+$ 331.07316, found 331.07348; for $C_{18}H_{15}ClNaO_4$ $[M + Na]^+$ 353.05511, found 353.05549, for $C_{18}H_{15}ClKO_4$ $[M + K]^+$ 369.02905, found 369.02950.

General procedure for the synthesis of β-enaminones 3a–h

Method A: The solution of the starting β-diketone **4** (3.8 mmol) and ammonium acetate (19 mmol) in methanol (45 mL) was refluxed for 5 h. The solution was then concentrated under reduced pressure. The residue was dissolved in DCM (15 mL) and washed with saturated aqueous $NaHCO_3$ (30 mL). The aqueous layer was extracted with DCM (2 × 15 mL). The organic layers were combined, washed with brine (1 × 15 mL), dried over anhydrous sodium sulfate and concentrated under reduced pressure. The pure products were isolated by a column chromatography, precipitation from the crude mixture or recrystallization (see details at individual compounds).

Method B: The suspension of 1,3-diketone **4d** (4.14 mmol) and NH_4HCO_3 (2 g, 25.3 mmol) in MeOH (10 mL) and THF (3 mL) was stirred for 24 h at room temperature. After filtration, the filtrate was dried over anhydrous Na_2SO_4 and concentrated under reduced pressure. The residue was subjected to a column chromatography (silica gel; DCM:EtOAc 10:1).

The following compounds were prepared according to the above-mentioned protocols:

(*2Z*)-3-Amino-5-(2-bromophenyl)-1-phenylpent-2-en-1-one (3a): ***Method A***, recrystallization from petroleum ether-EtOAc 10:1, yield 67%, white solid, mp 84–86 °C.

^{1}H NMR (400.13 MHz): δ = 10.21 (brs, 1H), 7.88–7.85 (m, 2H), 7.56 (d, *J* = 7.9 Hz, 1H), 7.47–7.38 (m, 3H), 7.27–7.22 (m, 2H), 7.12–7.08 (m, 1H), 5.78 (s, 1H), 5.27 (brs, 1H), 3.08–3.04 (m, 2H), 2.56–2.52 (m, 2H) ppm.

^{13}C NMR (100.62 MHz): δ = 190.1, 165.6, 140.4, 139.5, 133.2, 131.1, 130.8, 128.6, 128.4, 128.0, 127.3, 124.4, 92.0, 37.3, 35.4 ppm.

Elemental analysis: for $C_{17}H_{16}BrNO$ (330.2): calcd. C 61.83, H 4.88, N 4.24; found C 62.03, H 4.69, N 4.32.

(2*Z*)-3-Amino-5-(2-bromophenyl)-1-(3,4-dimethoxyphenyl)pent-2-en-1-one (3b): ***Method A.*** The product was precipitated from the crude yellow oil by diethyl ether. Yield 60%, light brown solid, mp 78–81 °C.

^{1}H NMR (400.13 MHz): δ = 10.13 (brs, 1H), 7.56 (d, *J* = 8 Hz, 2H), 7.51 (d, *J* = 2 Hz, 1H), 7.46 (dd, *J* = 2 Hz, 8.4 Hz, 1H), 7.25–7.23 (m, 2H), 7.12–7.08 (m, 1H), 6.86 (d, *J* = 8.4 Hz, 1H), 5.75 (s, 1H), 5.21 (brs, 1H), 3.94 (s, 3H), 3.92 (s, 3H), 3.09–3.05 (m, 2H), 2.56–2.52 (m, 2H) ppm.

^{13}C NMR (100.62 MHz): δ = 189.0, 165.0, 151.6, 148.9, 139.6, 133.3, 133.2, 130.7, 128.5, 127.9, 124.4, 120.8, 110.2, 110.1, 91.5, 56.13, 56.08, 37.3, 35.4 ppm.

Elemental analysis: for $C_{19}H_{20}BrNO_3$ (390.3): calcd. C 58.43, H 5.17, N 3.59; found C 58.67, H 5.11, N 3.39.

(2*Z*)-3-Amino-5-(2-bromophenyl)-1-(3-benzyloxy-4-methoxyphenyl)pent-2-en-1-one (3c): ***Method A.*** Column chromatography (silica gel; DCM:EtOAc 10:1). If necessary, the product can be further recrystallized from EtOH. Yield 75%, yellow solid, mp 105–106 °C.

^{1}H NMR (400.13 MHz): δ = 10.09 (br s, 1H), 7.58–7.56 (m, 1H), 7.54 (d, *J* = 2 Hz, 1H), 7.49–7.47 (m, 3H), 7.39–7.35 (m, 2H), 7.32–7.22 (m, 3H), 7.13 - 7.08 (m, 1H), 6.89 (d, *J* = 8.3 Hz, 1H), 5.68 (s, 1H), 5.20 (s, 2H), 5.10 (br s, 1H), 3.92 (s, 3H), 3.07–3.03 (m, 2H), 2.54–2.50 (m, 2H) ppm.

^{13}C NMR (100.62 MHz): δ = 189.0, 164.9, 152.4, 148.1, 139.6, 137.2, 133.22, 133.20, 130.8, 128.8, 128.5, 128.1, 128.0, 127.7, 124.4, 121.3, 112.8, 110.8, 91.5, 71.1, 56.2, 37.3, 35.4 ppm.

Elemental analysis: for $C_{25}H_{24}BrNO_3$ (466.4): calcd. C 64.38, H 5.19, N 3.00; found C 64.62, H 5.18, N 3.07.

(2*Z*)-3-Amino-1-(1,3-benzodioxol-5-yl)-5-(2-bromophenyl)pent-2-en-1-one (3d): ***Method B.*** Yield 47% of light yellow solid, mp 98–99 °C.

^{1}H NMR (400.13 MHz): δ = 10.09 (brs, 1H), 7.57–7.55 (m, 1H), 7.45–7.43 (m, 1H), 7.37 (s, 1H), 7.25–7.23 (m, 2H), 7.12–7.08 (m, 1H), 6.82 (d, *J* = 8.1 Hz, 1H), 6.00 (s, 2H), 5.68 (s, 1H), 5.23 (brs, 1H), 3.07–3.03 (m, 2H), 2.54–2.50 (m, 2H) ppm.

^{13}C NMR (100.62 MHz): δ = 188.6, 165.2, 150.1, 147.9, 139.6, 135.0, 133.2, 130.7, 128.5, 127.9, 124.4, 122.4, 107.9, 107.7, 101.6, 91.5, 37.3 35.4 ppm.

Elemental analysis: for $C_{18}H_{16}BrNO_3$ (374.2): calcd. C 57.77, H 4.31, N 3.74; found C 57.69, H 4.28, N 3.64.

(2*Z*)-3-Amino-5-(2-chlorophenyl)-1-phenylpent-2-en-1-one (3e): ***Method A.*** Column chromatography (silica gel; DCM:EtOAc 10:1). Yield 93% of white solid. Mp 73.5–75 °C.

^{1}H NMR (400.13 MHz): δ = 10.20 (br s, 1H), 7.87–7.84 (m, 2H), 7.46–7.34 (m, 4H), 7.23–7.15 (m, 3H), 5.76 (s, 1H), 5.33 (br s, 1H), 3.07–3.03 (m, 2H), 2.56–2.52 (m, 2H) ppm.

^{13}C NMR (100.62 MHz): δ = 190.0, 165.8, 140.4, 137.9, 131.1, 130.7, 129.8, 128.4, 128.3, 127.28, 127.26, 91.9, 37.1, 32.8 ppm.

Elemental analysis: for $C_{17}H_{16}ClNO$ (285.8): calcd. C 71.45, H 5.64, N 4.90; found C 71.60, H 5.58, N 4.83.

(2*Z*)-3-Amino-5-(2-chlorophenyl)-1-(3,4-dimethoxyphenyl)pent-2-en-1-one (3f): ***Method A.*** Column chromatography (silica gel; DCM:EtOAc 10:1). Yield 96% of yellow oil.

^{1}H NMR (400.13 MHz): δ = 10.13 (brs, 1H), 7.50 (s, 1H), 7.45 (dd, *J* = 8.3 Hz, 1.8 Hz, 1H), 7.36–7.34 (m, 1H), 7.21–7.15 (m, 3H), 6.85 (d, *J* = 8.3 Hz, 1H), 5.73 (s, 1H), 5.41 (brs, 1H), 3.93 (s, 3H), 3.91 (s, 3H), 3.07–3.03 (m, 2H), 2.55–2.51 (m, 2H) ppm.

^{13}C NMR (100.62 MHz): δ = 188.8, 165.1, 151.5, 148.7, 137.8, 133.8, 133.2, 130.6, 129.6, 128.1, 127.1, 120.6, 110.1, 110.0, 91.2, 56.0, 55.9, 36.9, 32.7 ppm.

Elemental analysis: for $C_{19}H_{20}ClNO_3$ (345.8): calcd. C 65.99, H 5.83, N 4.05; found C 65.70, H 5.87, N 3.92.

HRMS: *m/z* calculated for $C_{19}H_{21}ClNO_3$ $[M + H]^+$ 346.12045, found 346.12076; for $C_{19}H_{20}ClNNaO_3$ $[M + Na]^+$ 368.10239, found 368.10282; for $C_{19}H_{20}ClKNO_3$ $[M + K]^+$ 384.07633, found 384.07680.

(2*Z*)-3-Amino-5-(2-chlorophenyl)-1-(3-benzyloxy-4-methoxyphenyl)pent-2-en-1-one (3g): ***Method A.*** Column chromatography (silica gel; DCM:EtOAc 10:1). Yield 99% of yellow solid. Mp 118–119 °C.

^{1}H NMR (400.13 MHz): δ = 10.09 (br s, 1H), 7.54 (d, *J* = 2Hz, 1H), 7.48–7.46 (m, 3H), 7.39–7.34 (m, 3H), 7.32–7.27 (m, 1H), 7.23–7.16 (m, 3H), 6.88 (d, *J* = 8.5 Hz, 1H), 5.67 (s, 1H), 5.19 (s, 2H), 5.16 (br s, 1H), 3.92 (s, 3H), 3.06–3.02 (m, 2H), 2.54–2.50 (m, 2H) ppm.

^{13}C NMR (100.62 MHz): δ = 188.9, 165.0, 152.3, 148.0, 137.9, 137.1, 133.2, 130.8, 129.9, 128.7, 128.3, 128.1, 127.7, 127.3, 121.3, 112.7, 110.8, 91.5, 71.1, 56.2, 37.1, 32.9 ppm.

Elemental analysis: for $C_{25}H_{24}ClNO_3$ (421.9): calcd. C 71.17, H 5.73, N 3.32; found C 71.26, H 5.84, N 3.31.

HRMS *m/z* calculated for $C_{25}H_{25}ClNO_3$ $[M + H]^+$ 422.15175, found 422.15232; for $C_{25}H_{24}ClNNaO_3$ $[M + Na]^+$ 444.13369, found 444.13431; for $C_{25}H_{24}ClKNO_3$ $[M + K]^+$ 460.10763, found 460.10828.

(2*Z*)-3-Amino-1-(1,3-benzodioxol-5-yl)-5-(2-chlorophenyl)pent-2-en-1-one (3h): ***Method A.*** Column chromatography (silica gel; DCM:EtOAc 10:1). Yield 98% of yellow solid. Mp. 67–69 °C.

^{1}H NMR (400.13 MHz): δ = 10.10 (br s, 1H), 7.43 (dd, *J* = 8.2Hz, 1.6 Hz, 1H), 7.39–7.35 (m, 2H), 7.24 –7.16 (m, 3H), 6.81 (d, *J* = 8.3 Hz, 1H), 6.00 (s, 2H), 5.67 (s, 1H), 5.21 (br s, 1H), 3.07–3.03 (m, 2H), 2.55–2.51 (m, 2H) ppm.

^{13}C NMR (100.62 MHz): δ = 188.6, 165.2, 150.1, 147.9, 137.9, 135.0, 133.9, 130.7, 129.9, 128.3, 127.3, 122.4, 107.9, 107.7, 101.6, 91.3, 37.1, 32.9 ppm.

Elemental analysis: for $C_{18}H_{16}ClNO_3$ (329.8): calcd. C 65.56, H 4.89, N 4.25; found C 65.54, H 4.84, N 4.20.

HRMS *m/z* calculated for $C_{18}H_{17}ClNO_3$ $[M + H]^+$ 330.08915, found 330.08926; for $C_{18}H_{16}ClNNaO_3$ $[M + Na]^+$ 352.07109, found 352.07142; for $C_{18}H_{16}ClKNO_3$ $[M + K]^+$ 368.04503, found 368.04544.

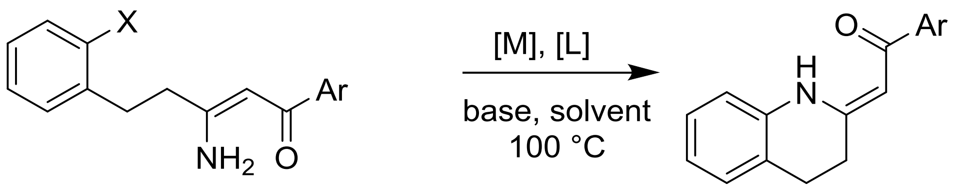

3a–h **1a–d**

General procedure for synthesis of tetrahydroquinolines 1a–d

For stoichiometric ratios, solvents and ligands see details at individual compounds.

Method A: An oven-dried vial equipped with a magnetic stir bar and fitted with a Teflon septum was charged with $Pd_2(dba)_3$ and corresponding ligand. The vessel was 3× evacuated and backfilled with argon. Subsequently the solvent (3 mL) was added via syringe and the mixture was preheated at 100 °C for 30 min. Another oven-dried vial was charged with the base and substrate **3**. The vessel was 3× evacuated and backfilled with argon. The solution of the activated catalyst was transferred from the first vial into the second one via syringe. The vessel was then heated at 100 °C until the starting component was fully consumed (control by TLC). The mixture was then diluted with EtOAc and filtered through a small plug of Celite® S which was subsequently thoroughly washed with EtOAc. The filtrate was concentrated *in vacuo* and the residue was purified by recrystallization or a column chromatography (see details at individual compounds).

Method B: An oven-dried vial equipped with a magnetic stir bar and fitted with a Teflon septum was charged with palladium diacetate (5.6 mg, 5 mol %) and XPhos (35.8 mg, 15 mol %). The vessel was 3× evacuated and backfilled with argon. Subsequently

toluene (3 mL) was added via syringe, the vial was opened and water (1.8 μl, 20 mol. %) was added via micropipette. The vial was closed and flushed with argon. The mixture was heated at 100 °C for 1.5 min. Another oven-dried vial equipped with a magnetic stir bar and fitted with a Teflon septum was charged with caesium carbonate (228 mg, 1.4 equiv) and **3a** (165 mg, 0.5 mmol), 3× evacuated and backfilled with argon. The content of the first vial (active catalyst) was transferred via syringe into the second vial (with the substrate and base). The mixture was heated at 100 °C for 21 h, then cooled and filtered through a small plug of Celite® S which was subsequently thoroughly washed with EtOAc. The filtrate was concentrated *in vacuo*.

Method C: An oven-dried vial equipped with magnetic stir bar and fitted with a Teflon septum was charged with CuI, ligand, base and substrate **3a** (for details see Table 1, Entries 6–9). The vessel was 3× evacuated and backfilled with argon. Subsequently dry toluene (5 mL) was added via syringe and the mixture was heated at 100 °C for 18–20 h. The mixture was cooled and transferred into a separatory funnel. Conc. aqueous ammonia (1 mL) was then added together with EtOAc (5 mL) and water (10 mL). The aqueous layer was extracted with EtOAc (3 × 5 mL). The combined organic layers were washed with brine, dried over anhydrous sodium sulfate and evaporated *in vacuo*.

Method D: like method C, but **L6** was used instead of CuI and [L]. The reaction was quenched by diluting the mixture with EtOAc and filtering through a small plug of Celite® S which was subsequently thoroughly washed with EtOAc.

The following compounds were prepared using the above-mentioned procedures:

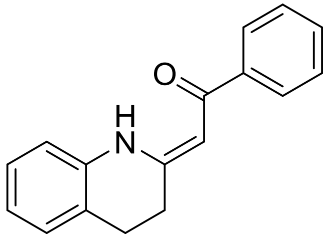

1-Phenyl-2-((2*Z*)-1,2,3,4-tetrahydroquinolin-2-ylidene)ethan-1-one (1a): ***Method A:*** from **3a**, 3.5 mol % $Pd_2(dba)_3$, 7 mol % XPhos, 1.4 equiv Cs_2CO_3, toluene, 2 h. Column chromatography (silica gel; DCM:EtOAc 10:1). Yield 92%. From **3e**, 5 mol % $Pd_2(dba)_3$, 10 mol % *t*-BuXPhos, 1.4 equiv Cs_2CO_3, *t*-AmOH, 17 h. Column chromatography (silica gel; DCM). Yield 72%. ***Method C:*** from **3a**, 10 mol % CuI, 20 mol % DESA, 1.4 equiv Cs_2CO_3, toluene, 18 h. Column chromatography (silica gel; DCM:EtOAc 10:1), yield 83%. Yellow solid, mp 103–105 °C (ref.[14] reports 105–106 °C). Proton NMR data are in accordance with [15].

^{1}H NMR (400.13 MHz): δ = 12.85 (brs, 1H), 7.94–7.91 (m, 2H), 7.50–7.42 (m, 3H), 7.21–7.17 (m, 1H), 7.12–7.10 (m, 1H), 6.99–6.94 (m, 2H), 5.88 (s, 1H), 2.89–2.86 (m, 2H), 2.75–2.71 (m, 2H) ppm.

^{13}C NMR (100.62 MHz): δ = 189.7, 159.0, 139.9, 136.7, 131.3, 128.5, 128.4, 127.9, 127.3, 125.3, 123.3, 116.8, 92.6, 28.8, 24.4 ppm.

Crystallographic data for **1a**: $C_{17}H_{15}NO$, M = 249.30, monoclinic, $P2_1/c$, *a* = 6.0830(4), *b* = 19.8091(9), *c* = 10.7900(6) Å, β = 100.172(6) °, Z = 4, V = 1279.75(13) Å^3, D_c = 1.294 g.cm^{-3}, μ = 0.080 mm^{-1}, T_{min}/T_{max} = 0.985/ 0.991; -6 ≤ h ≤ 7, -25 ≤ k ≤ 24, -11 ≤ l

≤ 13; 10576 reflections measured (θ_{max} = 27.5 °), 2876 independent (R_{int} = 0.0611), 2181 with $I > 2\sigma(I)$, 172 parameters, *S* = 1.098, *R1*(obs. data) = 0.0504, *wR2*(all data) = 0.1078; max., min. residual electron density = 0.281, -0.260 eÅ^{-3}.

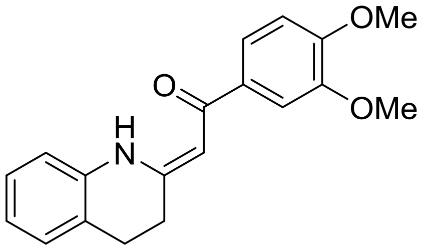

1-(3,4-Dimethoxyphenyl-2-((2*Z*)-1,2,3,4-tetrahydroquinolin-2-ylidene)ethan-1-one (1b): ***Method A:*** from **3b**, 3.5 mol % $Pd_2(dba)_3$, 7 mol % XPhos, 1.4 equiv Cs_2CO_3, toluene, 2 h. Recrystallization from toluene. Yield 65%. From **3f**, 5 mol % $Pd_2(dba)_3$, 10 mol % *t*-BuXPhos, *t*-AmOH, 1.4 equiv Cs_2CO_3, 20 h. Column chromatography (silica gel; DCM:EtOAc 10:1). Yield 72%. Yellow solid, mp 156–159 °C. NMR data are consistent with those published in ref.[16]

^{1}H NMR (400.13 MHz): δ = 12.79 (brs, 1H), 7.56 (d, J = 2 Hz, 1H), 7.52 (dd, *J* = 8.3 Hz, 1.8 Hz, 1H), 7.18 (t, *J* = 7.7 Hz, 1H), 7.10 (d, *J* = 7.4 Hz, 1H), 6.98–6.87 (m, 3H), 5.85 (s, 1H), 3.97 (s, 3H), 3.93 (s, 3H), 2.88–2.84 (m, 2H), 2.74–2.70 (m, 2H) ppm.

^{13}C NMR (100.62 MHz): δ = 188.6, 158.5, 151.9, 149.0, 136.9, 132.9, 128.3, 127.9, 125.2, 123.1, 120.9, 116.7, 110.3, 110.0, 92.1, 56.14, 56.10, 28.9, 24.5 ppm.

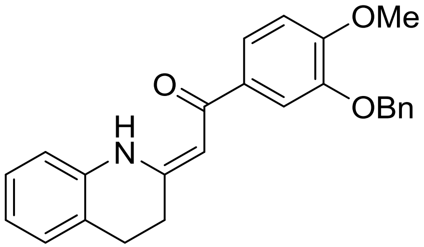

1-(3-Benzyloxy-4-methoxyphenyl-2-((2*Z*)-1,2,3,4-tetrahydroquinolin-2-ylidene)ethan-1-one (1c):

Method A: from **3c**, 5 mol % $Pd_2(dba)_3$, 10 mol % XPhos, toluene, 1.4 equiv Cs_2CO_3, 2 h. Column chromatography (silica gel; DCM:EtOAc 10:1). Yield 85%. From **3g**, 5 mol % $Pd_2(dba)_3$, 10 mol % *t*-BuXPhos, 1.4 equiv Cs_2CO_3, *t*-AmOH, 19 h. Column chromatography (silica gel; DCM:EtOAc 10:1). Yield 82%. Yellow solid with mp 93.5–94.5 °C.

^{1}H NMR (500.20 MHz): δ = 12.75 (br s, 1H), 7.61 (d, *J* = 2.1 Hz, 1H), 7.55 (dd, *J* = 8.4 Hz, 2.0 Hz, 1H), 7.50–7.48 (m, 2H), 7.40–7.37 (m, 2H), 7.33–7.30 (m, 1H), 7.8 (t, *J* = 7.6 Hz, 1H), 7.11 (d, *J* = 7.2 Hz, 1H), 6.96 (t, *J* = 7.6 Hz, 1H), 6.93–6.91 (m, 2H), 5.80 (s, 1H), 5.22 (s, 2H), 3.94 (s, 3H), 2.88–2.85 (m, 2H), 2.73–2.70 (m, 2H) ppm.

^{13}C NMR (125.79 MHz): δ = 188.7, 158.4, 152.6, 148.2, 137.1, 136.9, 132.8, 128.8, 128.4, 128.2, 128.0, 127.8, 125.2, 123.1, 121.4, 116.8, 112.5, 110.8, 92.2, 71.1, 56.3, 28.9, 24.6 ppm.

Elemental analysis: for $C_{25}H_{23}NO_3$ (385.5): calcd. C 77.90, H 6.01, N 3.63; found C 77.81, H 6.20, N 3.54.

HRMS: *m/z* calculated for $C_{25}H_{24}NO_3$ $[M + H]^+$ 386.17507, found 386.17526; for $C_{25}H_{23}NNaO_3$ $[M + Na]^+$ 408.15732, found 408.15701.

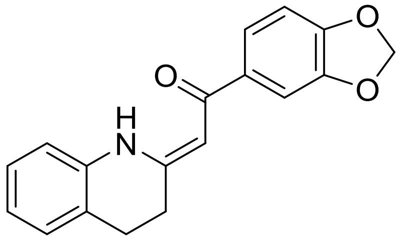

1-(2*H*-1,3-Benzodioxol-5-yl)-2-((2*Z*)-1,2,3,4-tetrahydroquinolin-2-ylidene)ethan-1-one (1d):

Method A: from **3d**, 3.5 mol % $Pd_2(dba)_3$, 7 mol % XPhos, 1.4 equiv Cs_2CO_3, toluene, 2 h. Recrystallization from isopropyl alcohol. Yield 45 %. From **3h**, 5 mol % $Pd_2(dba)_3$, 10 mol % *t*-BuXPhos, 1.4 equiv Cs_2CO_3, *t*-AmOH, 20 h. Column chromatography (silica gel; DCM:EtOAc 10:1). Yield 61%. Yellow solid with mp 139–142 °C.

^{1}H NMR (400.13 MHz): δ = 12.74 (brs, 1H), 7.50 (dd, *J* = 1.8 Hz, 8.2 Hz, 1H), 7.43 (d, *J* = 1.7 Hz 1H), 7.17 (t, *J* = 7.7 Hz, 1H), 7.09 (d, *J* = 7.3 Hz, 1H), 6.97–6.91 (m, 2H), 6.84 (d, *J* = 8.1 Hz, 1H), 6.01 (s, 2H), 5.77 (s, 1H), 2.87–2.83 (m, 2H), 2.72–2.68 (m, 2H) ppm. ***^{13}C NMR (100.62 MHz):*** δ = 188.3, 158.6, 150.4, 148.1, 136.8, 134.6, 128.3, 127.9, 125.2, 123.1, 122.5, 116.8, 108.0, 107.6, 101.7, 92.2, 28.9, 24.5 ppm.

Elemental analysis: for $C_{18}H_{15}NO_3$ (293.3): calcd. C 73.71, H 5.15, N 4.78; found C 73.90, H 5.24, N 4.60.

HRMS: *m/z* calculated for $C_{18}H_{16}NO_3$ $[M+H]^+$ 294.11247, found 294.11101; for $C_{18}H_{14}NO_3$ $[M-H]^+$ 292.09682, found 292.09621.

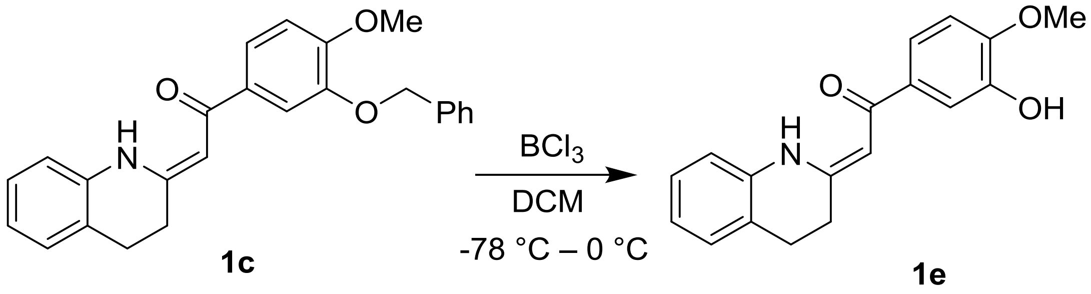

1-(3-Hydroxy-4-methoxyphenyl-2-((2*Z*)-1,2,3,4-tetrahydroquinolin-2-ylidene)ethan-1-one (1e): To a stirred solution of tetrahydroquinoline **1c** (77.1 mg, 0.2 mmol) in dry DCM (10 mL), 1M solution of BCl_3 in DCM (0.6 mL, 0.6 mmol) was added dropwise at -78 °C. The mixture was stirred for 30 min. at -78 °C and 3 h at 0 °C. Afterwards, the reaction was quenched with saturated aqueous $NaHCO_3$ (20 mL) and the mixture was extracted with DCM (2 × 60 mL). The combined organic layers were washed with brine (100 mL) and dried over anhydrous Na_2SO_4. The solvent was removed *in vacuo* and the residue was subjected to a column chromatography (silica gel; DCM:EtOAc 20:1). Yield: 38.8 mg (66%) of yellow solid with mp 166–167.5 °C.

^{1}H NMR (400.13 MHz): δ = 12.80 (brs, 1H), 7.54–7.51 (m, 2H), 7.18 (t, *J* = 7.7 Hz, 1H), 7.10 (d, *J* = 7.3 Hz, 1H), 6.98–6.93 (m, 2H), 6.91–6.89 (m, 1H), 5.81 (brs, 1H), 3.95 (s, 3H), 2.88–2.85 (m, 2H), 2.73–2.70 (m, 2H) ppm.

^{13}C NMR (100.62 MHz): δ = 188.8, 149.4, 145.5, 136.9, 133.5, 128.4, 127.9, 125.3, 123.2, 123.2, 120.3, 116.9, 113.7, 110.2, 56.2, 28.9, 24.5 ppm.

Elemental analysis: for $C_{18}H_{17}NO_3$ (295.3): calcd. C 73.20, H 5.80, N 4.74; found C 72.93, H 6.00, N 4.59.

HRMS: *m/z* calculated for $C_{18}H_{18}NO_3$ $[M+H]^+$ 296.12812, found 296.12801; for $C_{18}H_{17}NNaO_3$ $[M+Na]^+$ 318.11007, found 318.11038.

Proton and carbon NMR spectra of compounds 1a–e, 3a–h, 4a–h

Figure S1: 400 MHz ^{1}H NMR spectrum of the compound **4a**.

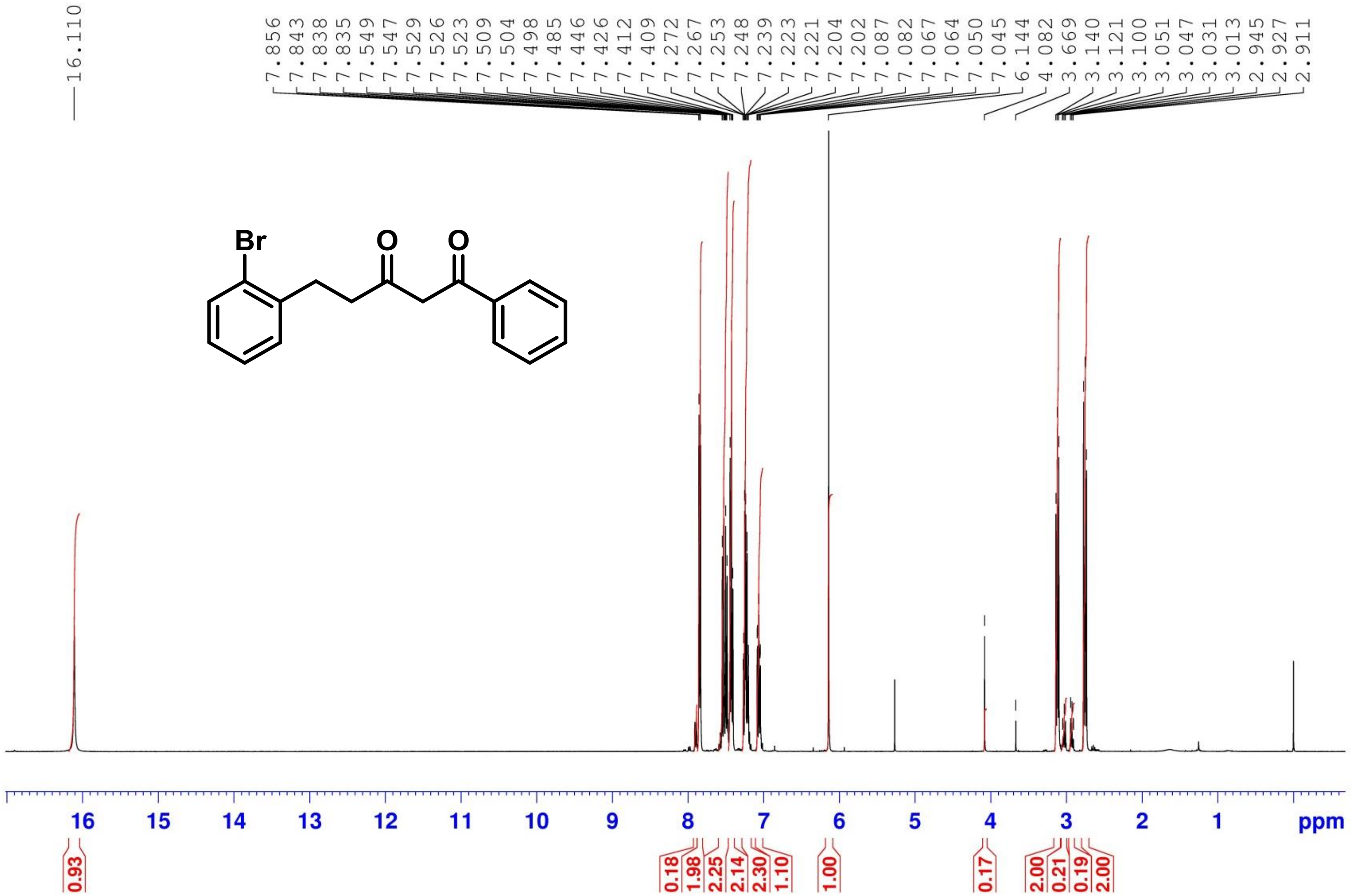

Figure S1a: 400 MHz ^{1}H NMR spectrum of the compound **4a**, detail.

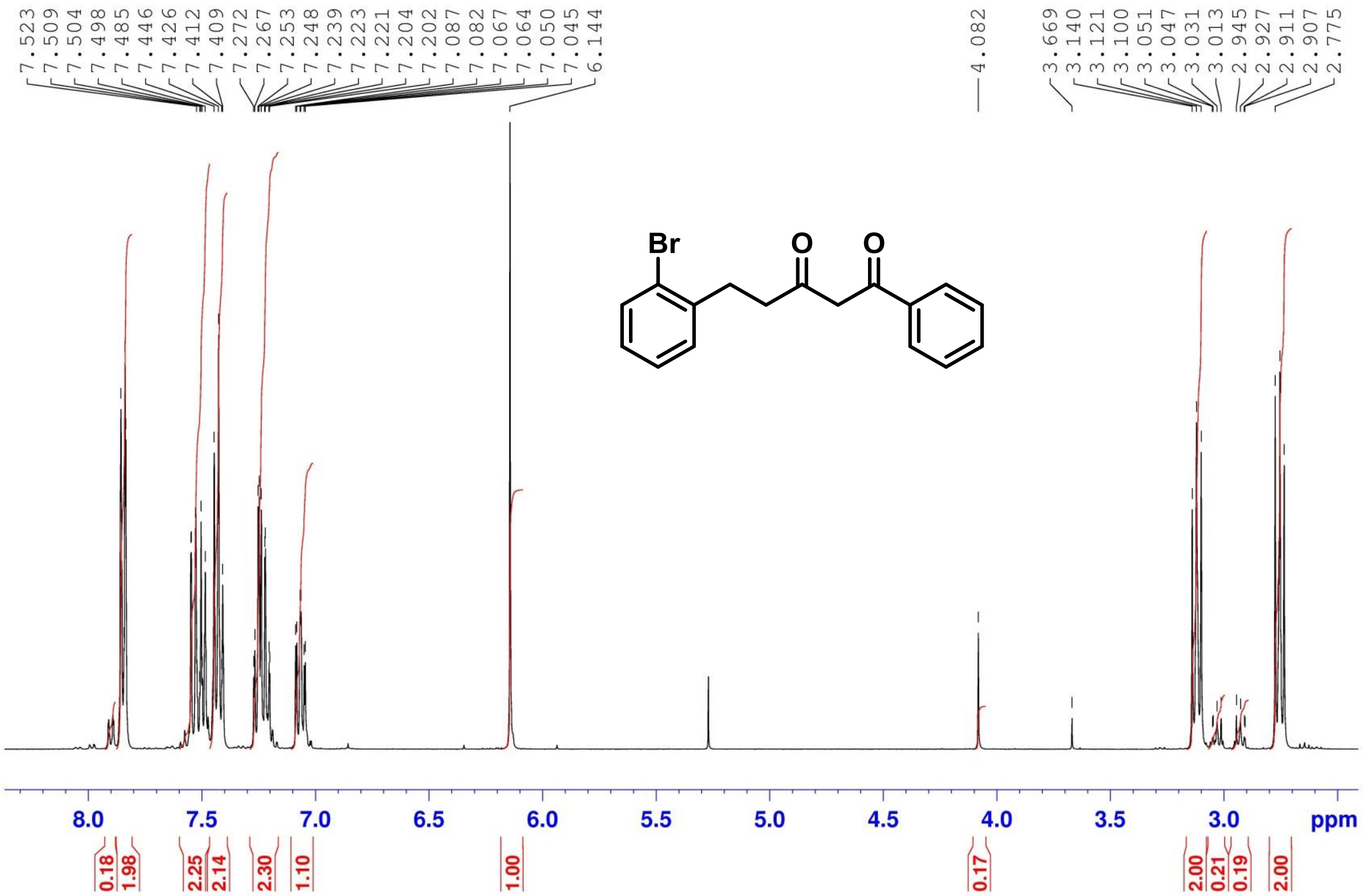

Figure S2: 100 MHz APT spectrum of the compound **4a**.

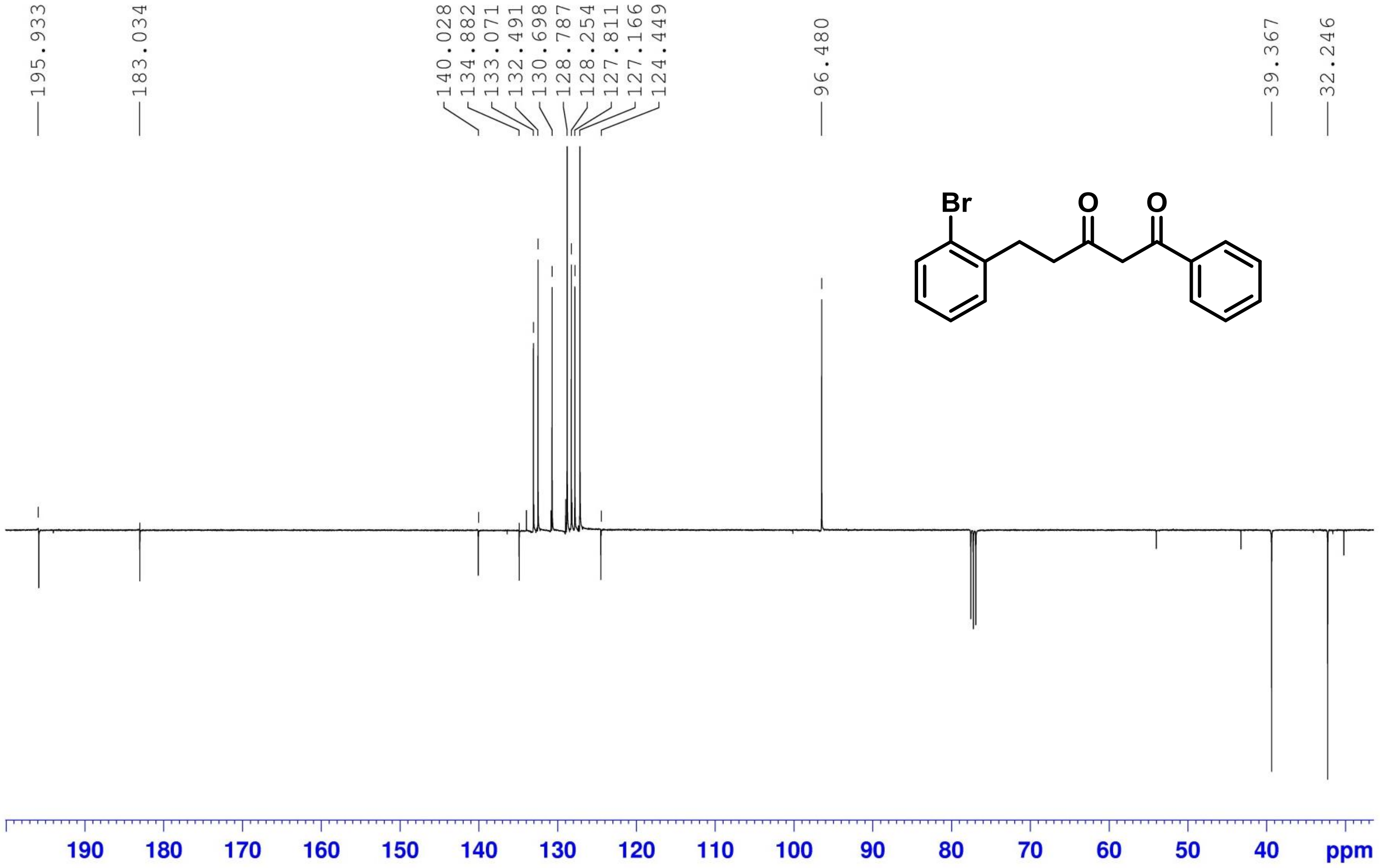

Figure S3: 400 MHz ^{1}H spectrum of the compound **4b**.

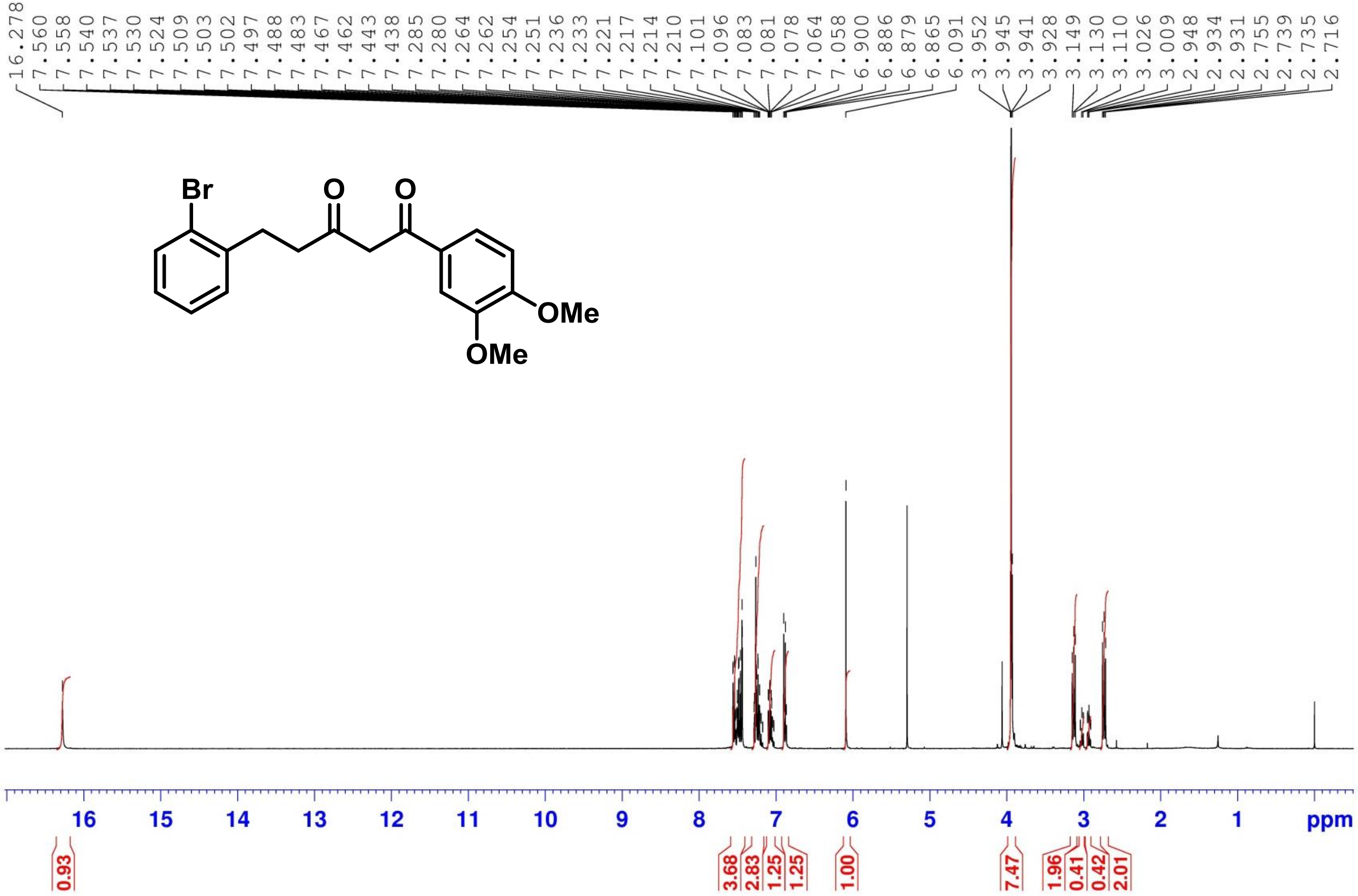

Figure S3a: 400 MHz ^{1}H spectrum of the compound **4b**, detail.

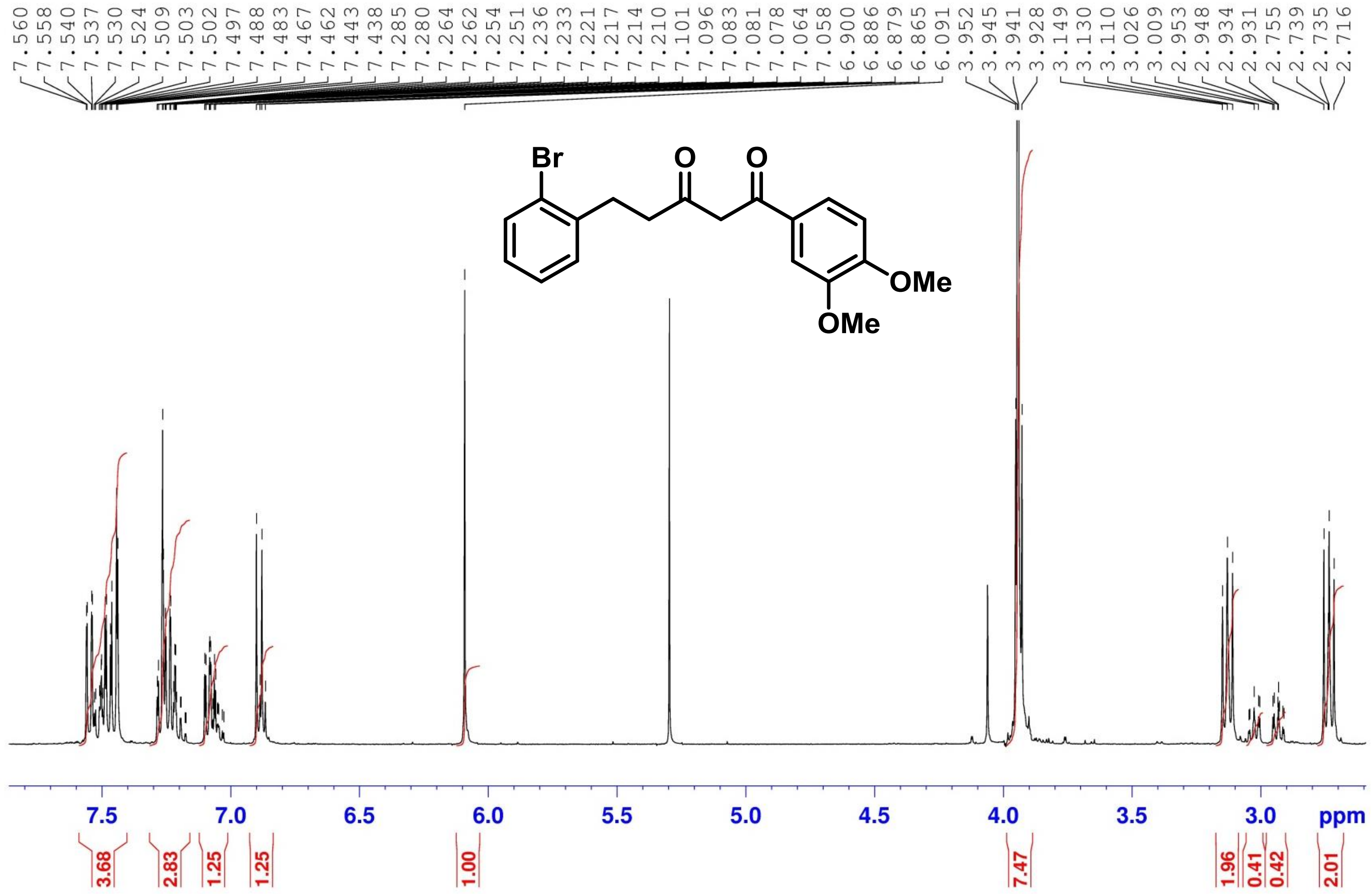

Figure S4: 100 MHz ^{13}C NMR spectrum of the compound **4b**.

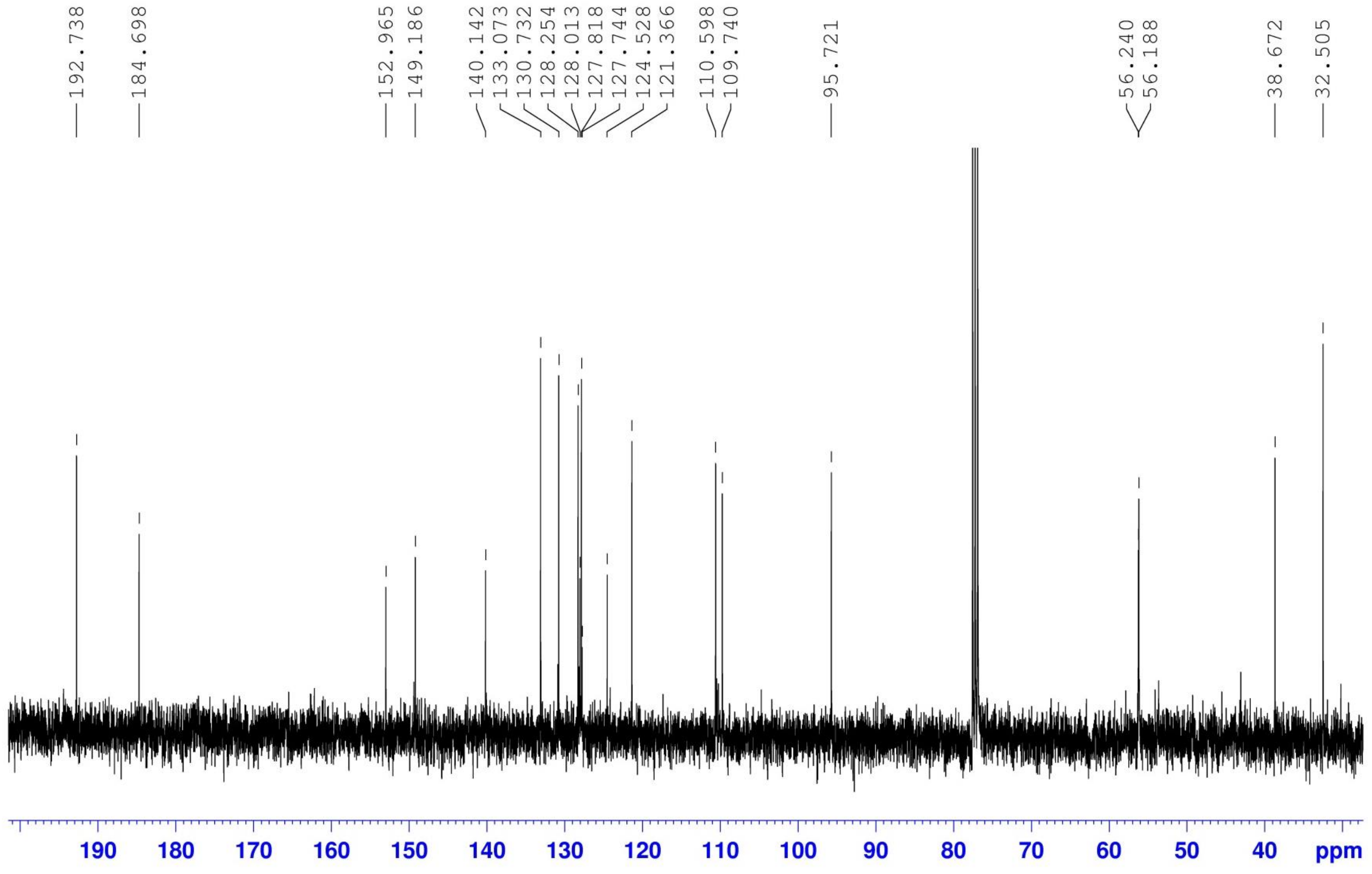

Figure S5: 400 MHz ^{1}H NMR spectrum of the compound **4c**.

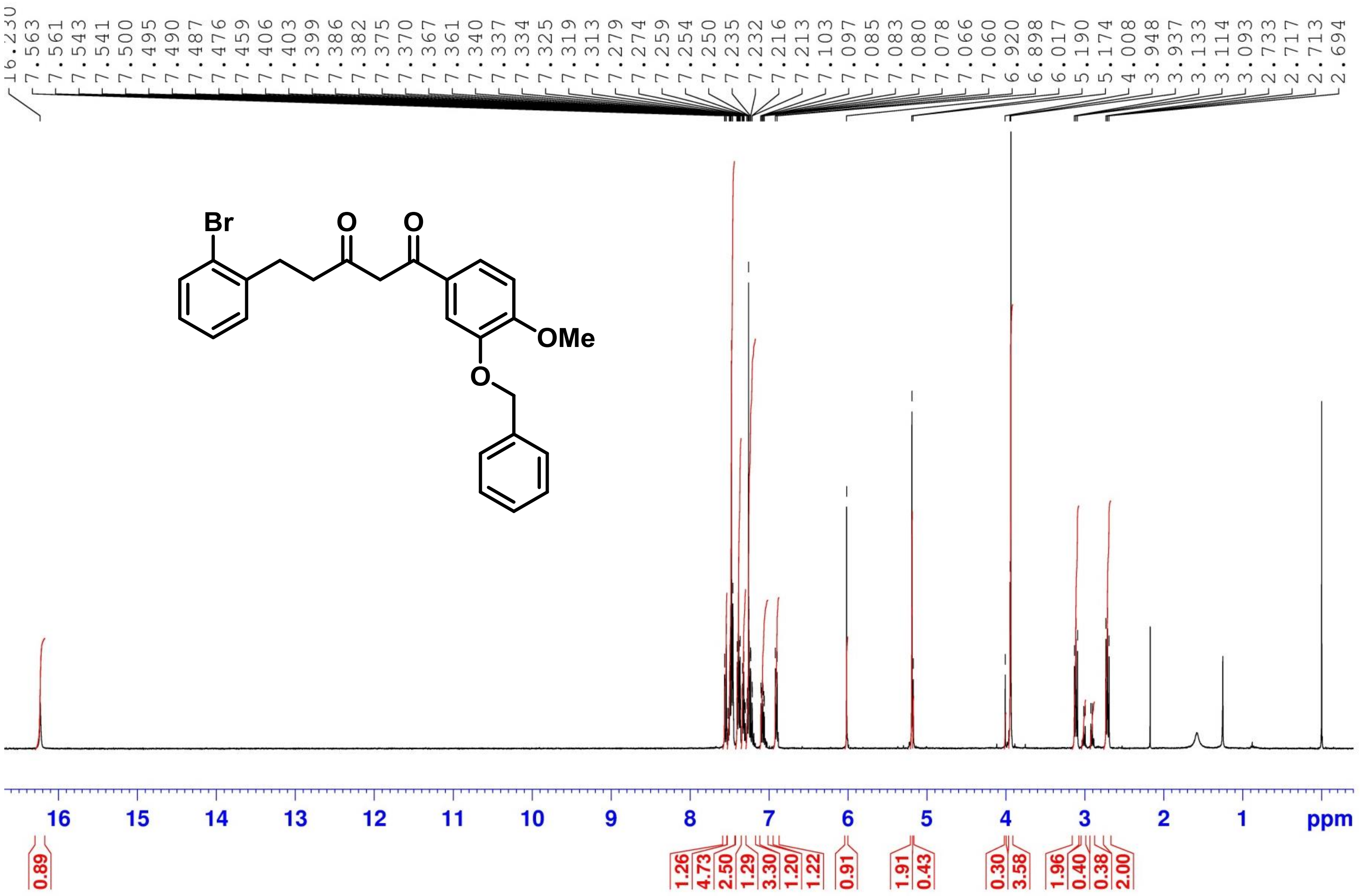

Figure S5a: 400 MHz ^{1}H NMR spectrum of the compound 4**c**, a detail.

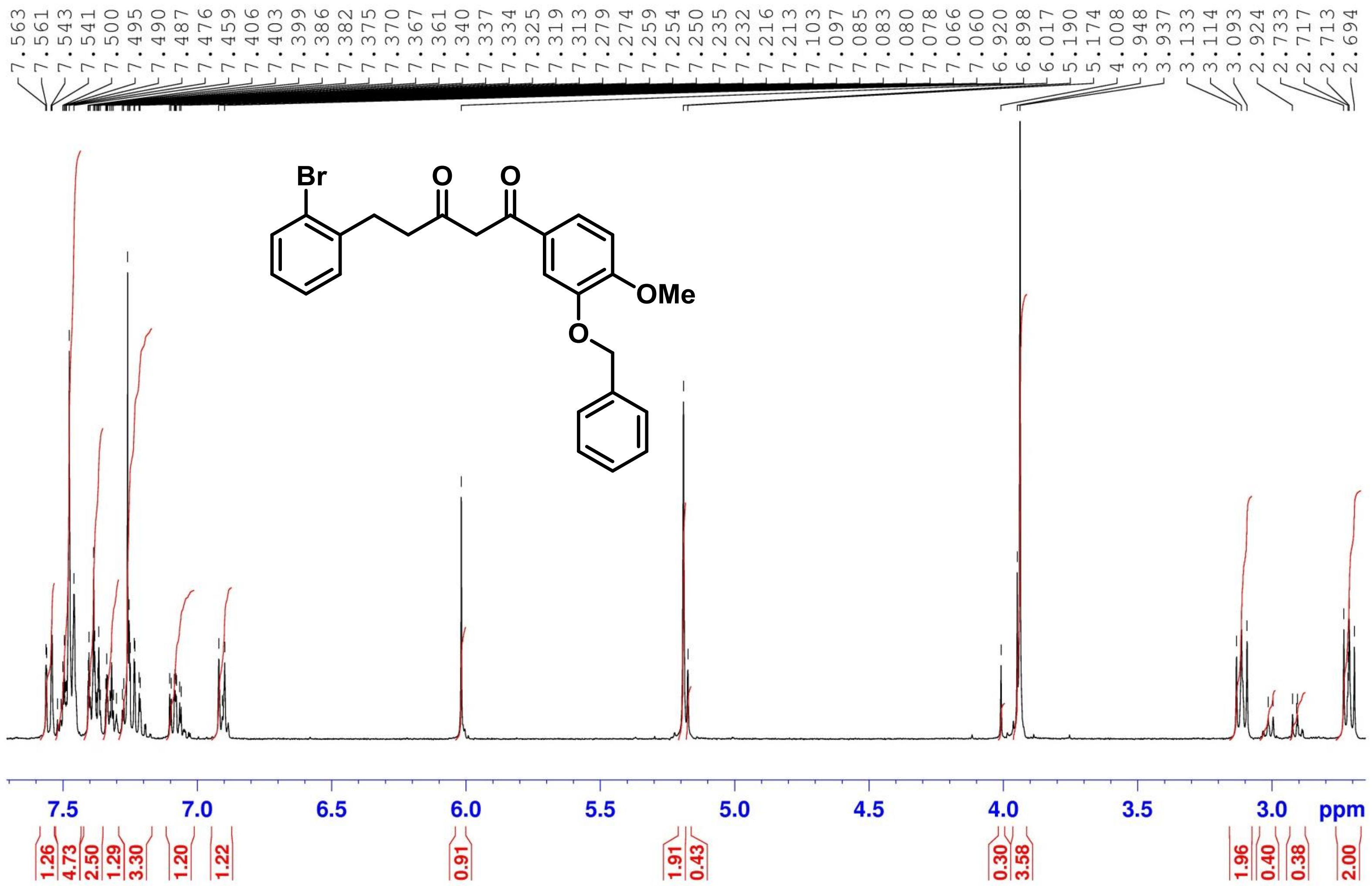

Figure S6: 100 MHz ^{13}C NMR spectrum of the compound **4c**.

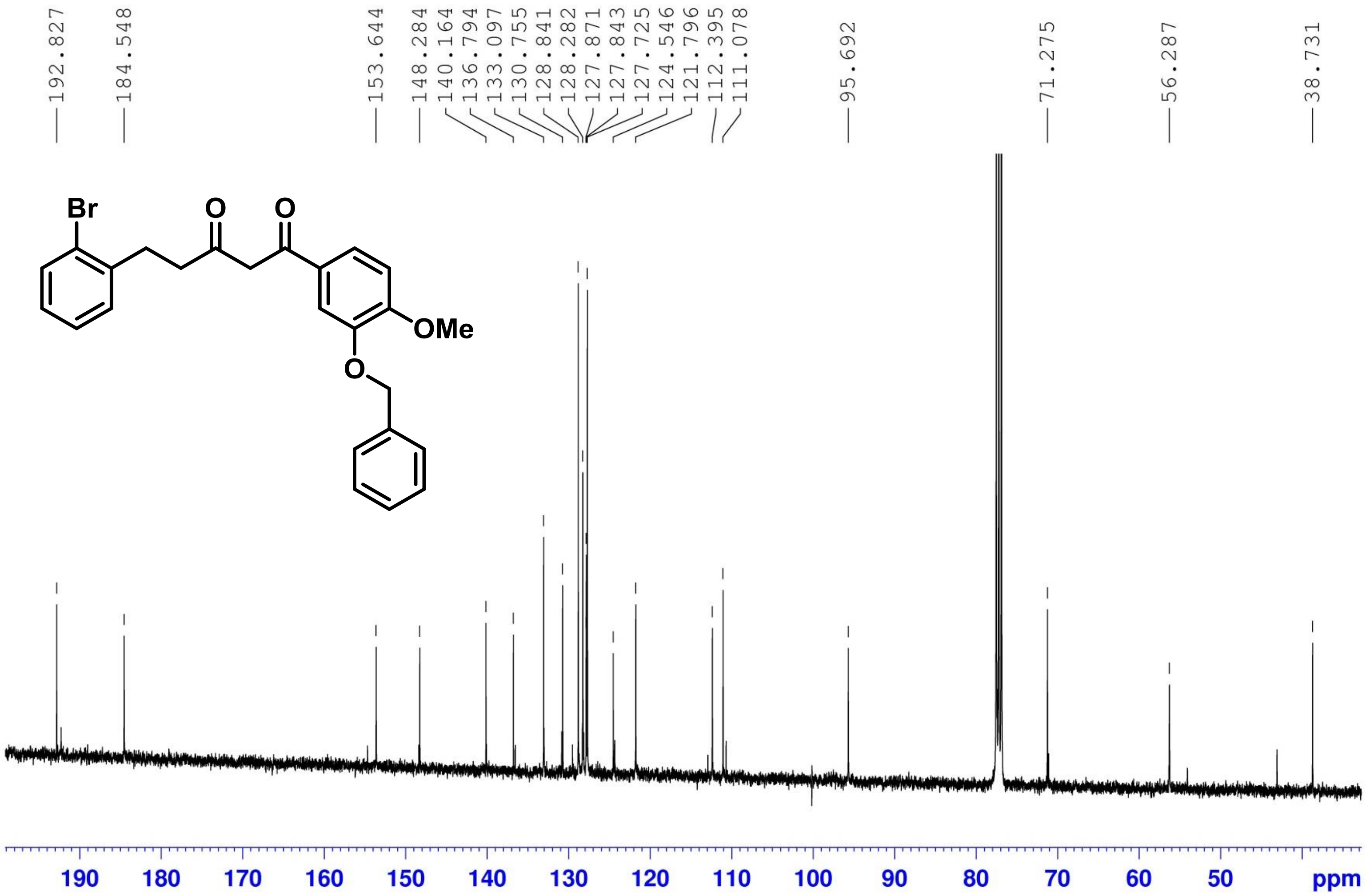

Figure S7: 400 MHz ^{1}H NMR spectrum of the compound **4d**.

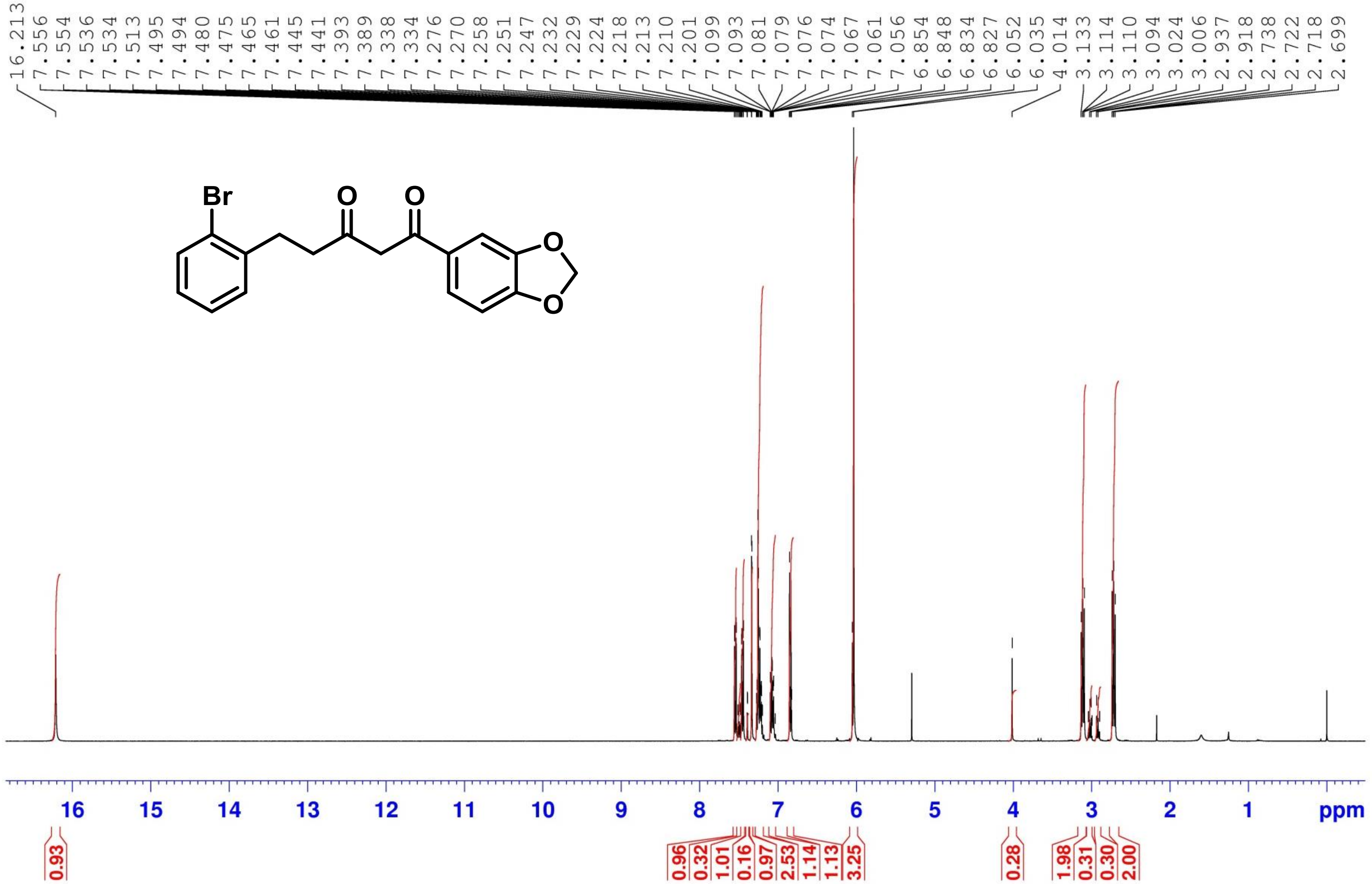

Figure S7a: 400 MHz ^{1}H NMR spectrum of the compound **4d**, a detail.

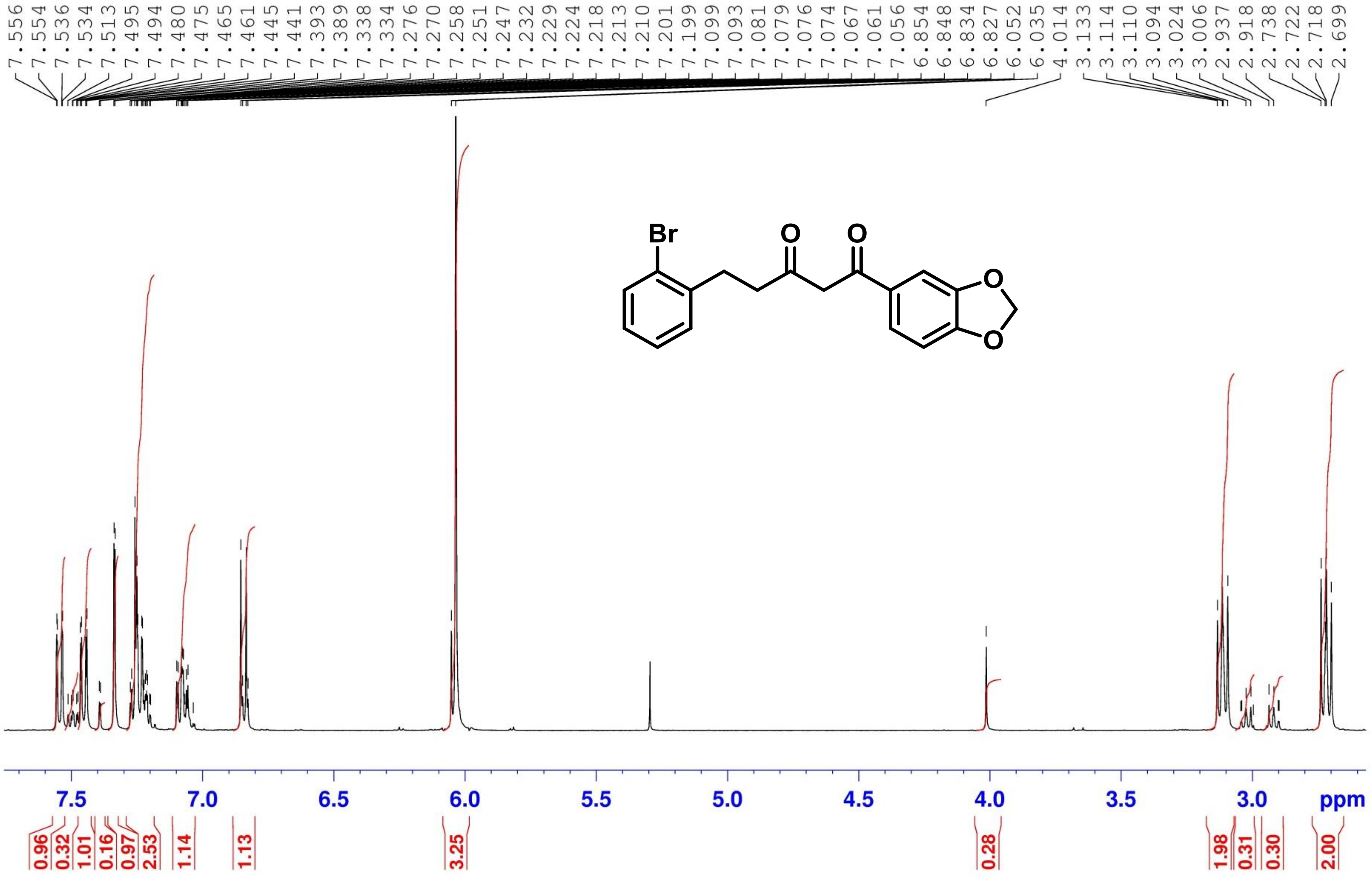

Figure S8: 100 MHz ^{13}C NMR spectrum of the compound **4d**.

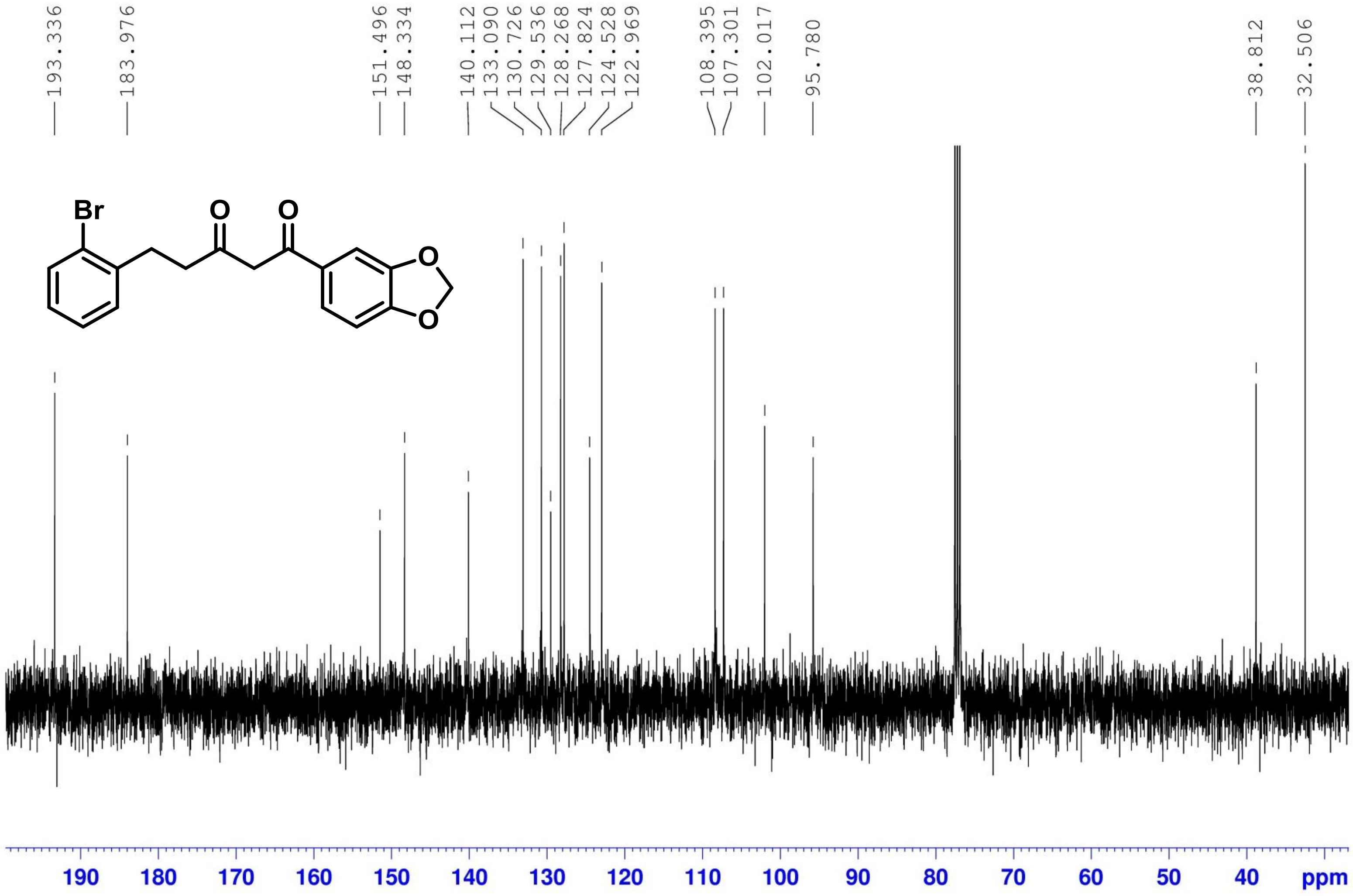

Figure S9: 500 MHz ^{1}H NMR spectrum of the compound **4e**.

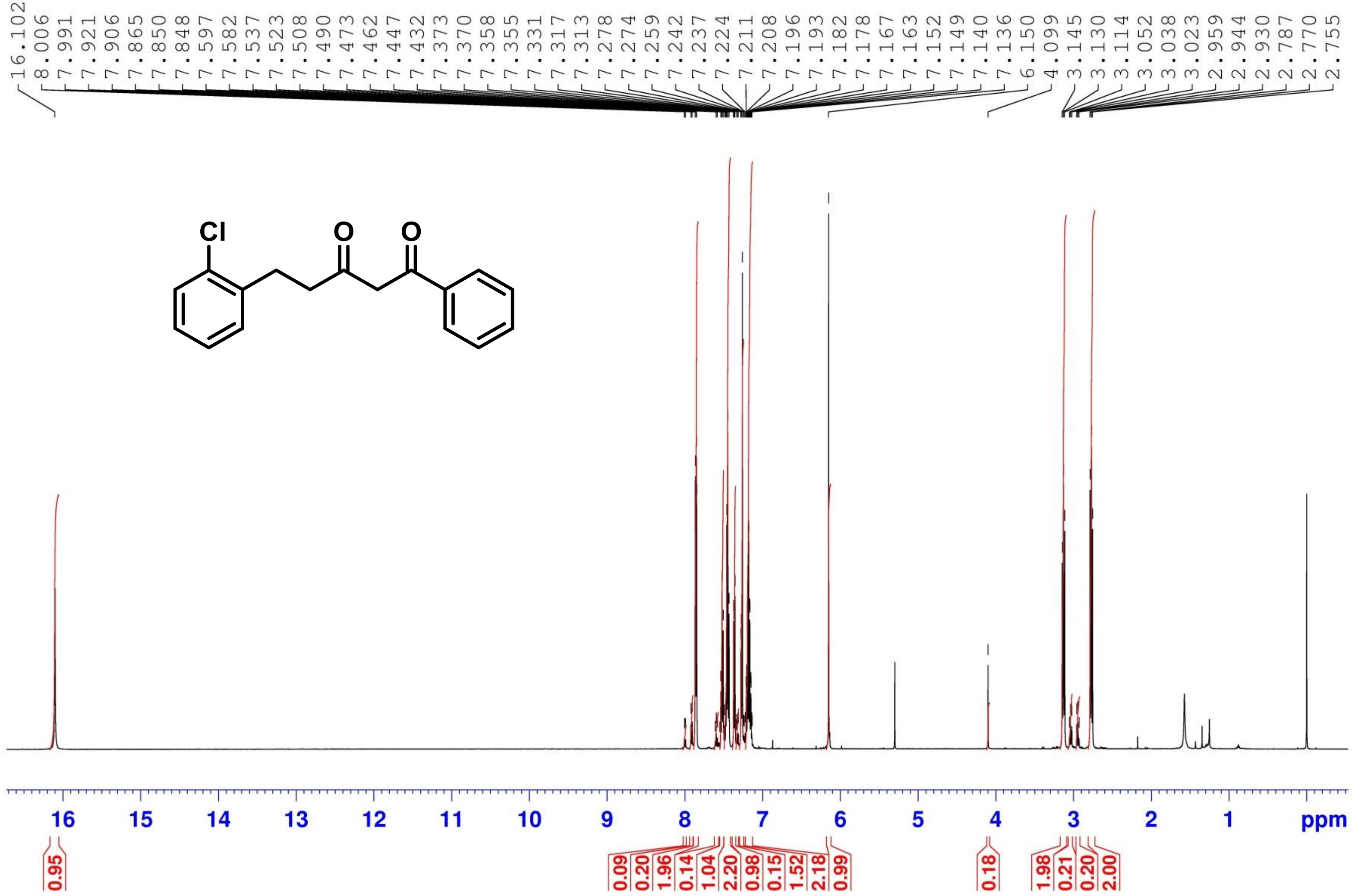

Figure S9a: 500 MHz ^{1}H NMR spectrum of the compound **4e**, a detail.

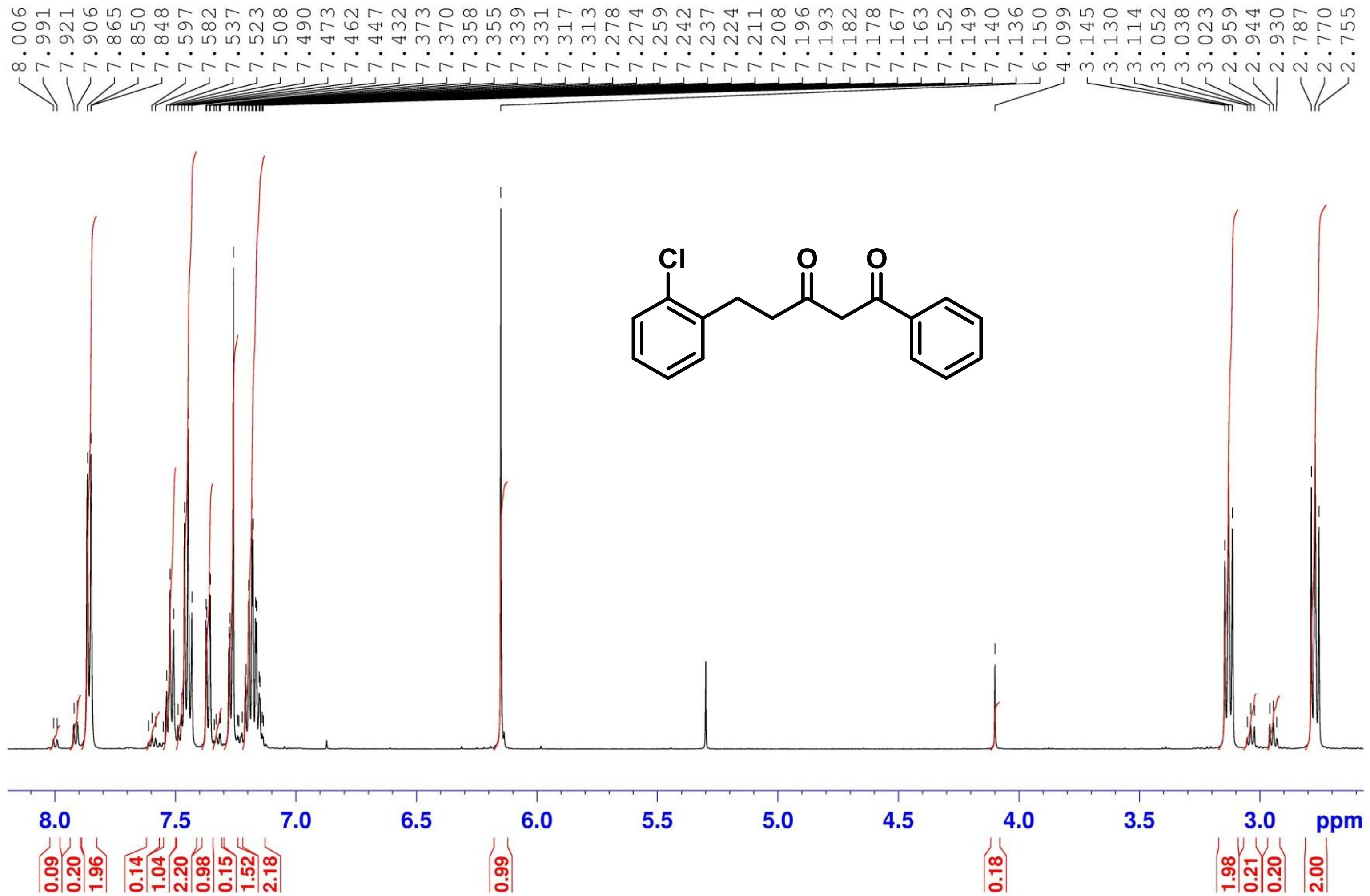

Figure S10: 125 MHz ^{13}C NMR spectrum of the compound **4e**.

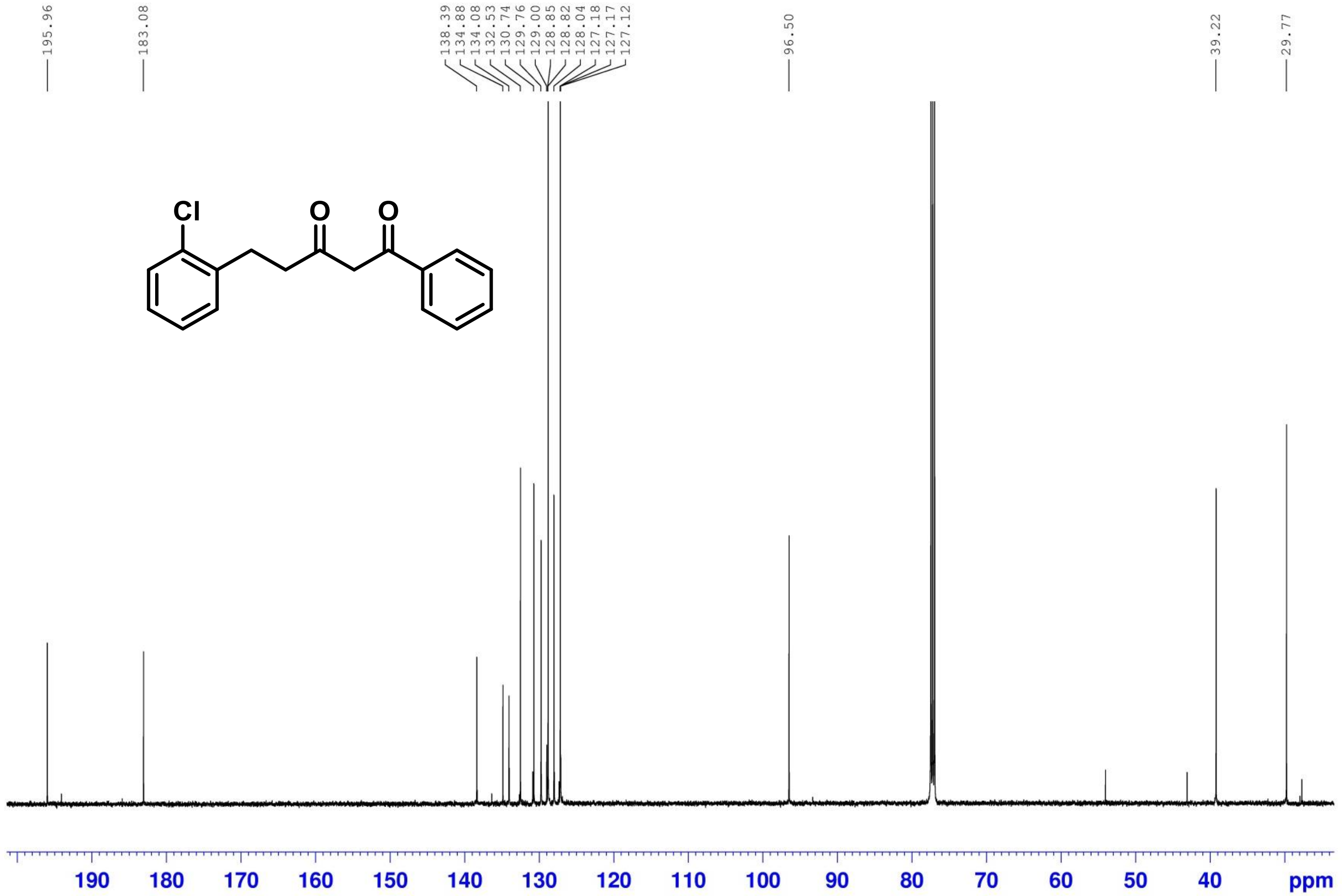

Figure S11: 500MHz ^{1}H NMR spectrum of the compound **4f**.

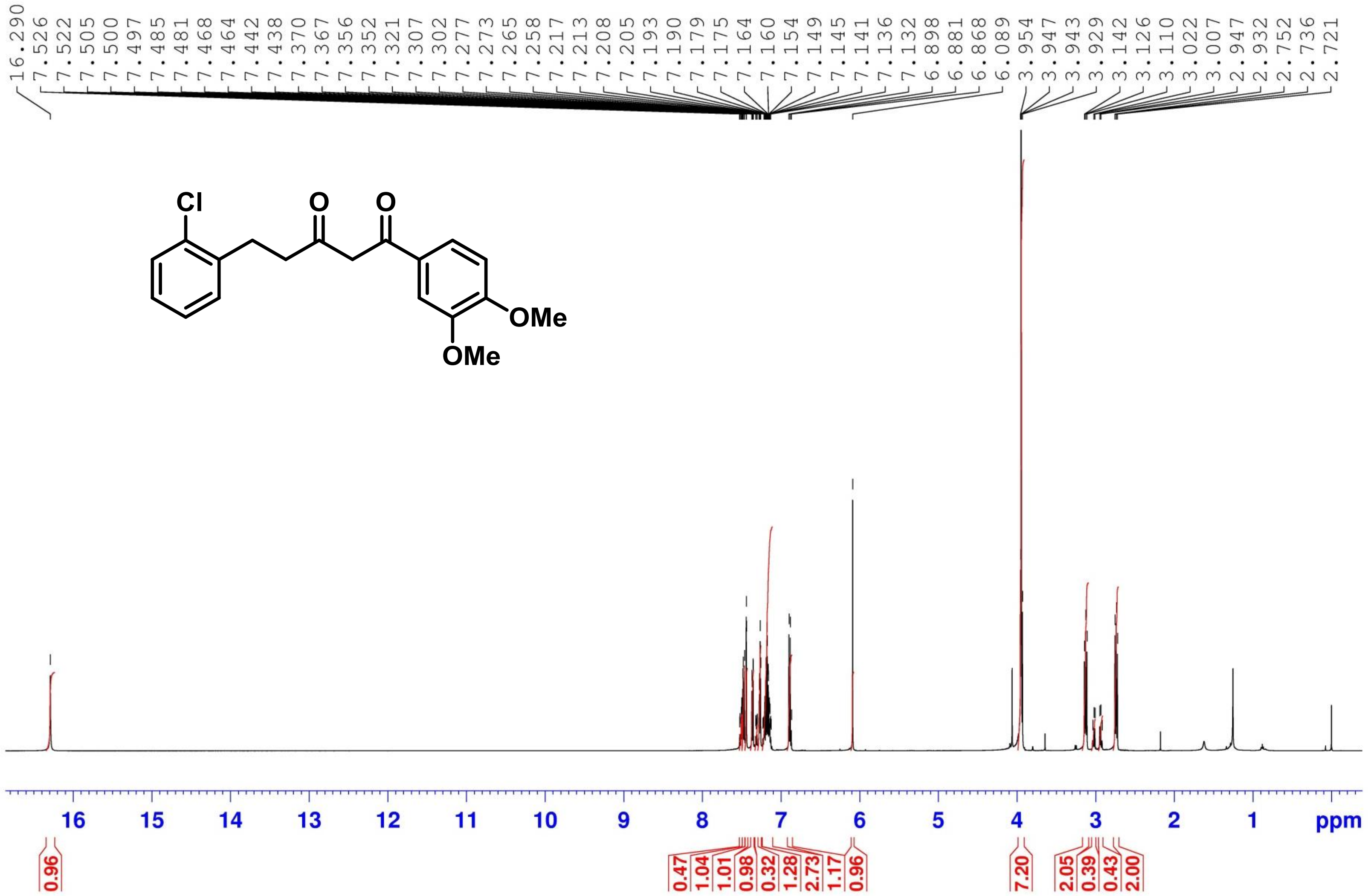

Figure S11a: 500 MHz ^{1}H NMR spectrum of the compound **4f**, a detail.

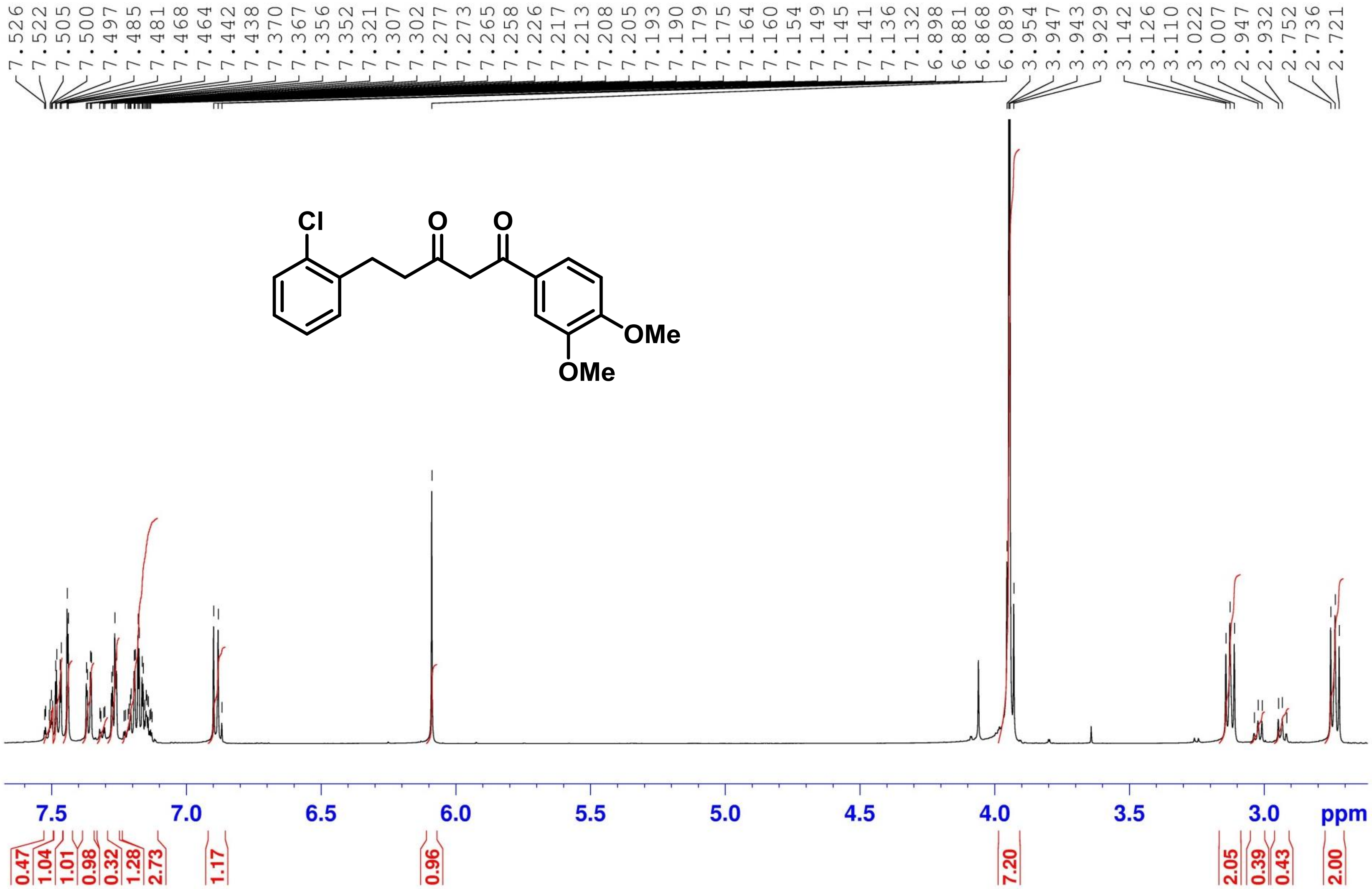

Figure S12: 125 MHz ^{13}C NMR spectrum of the compound **4f**.

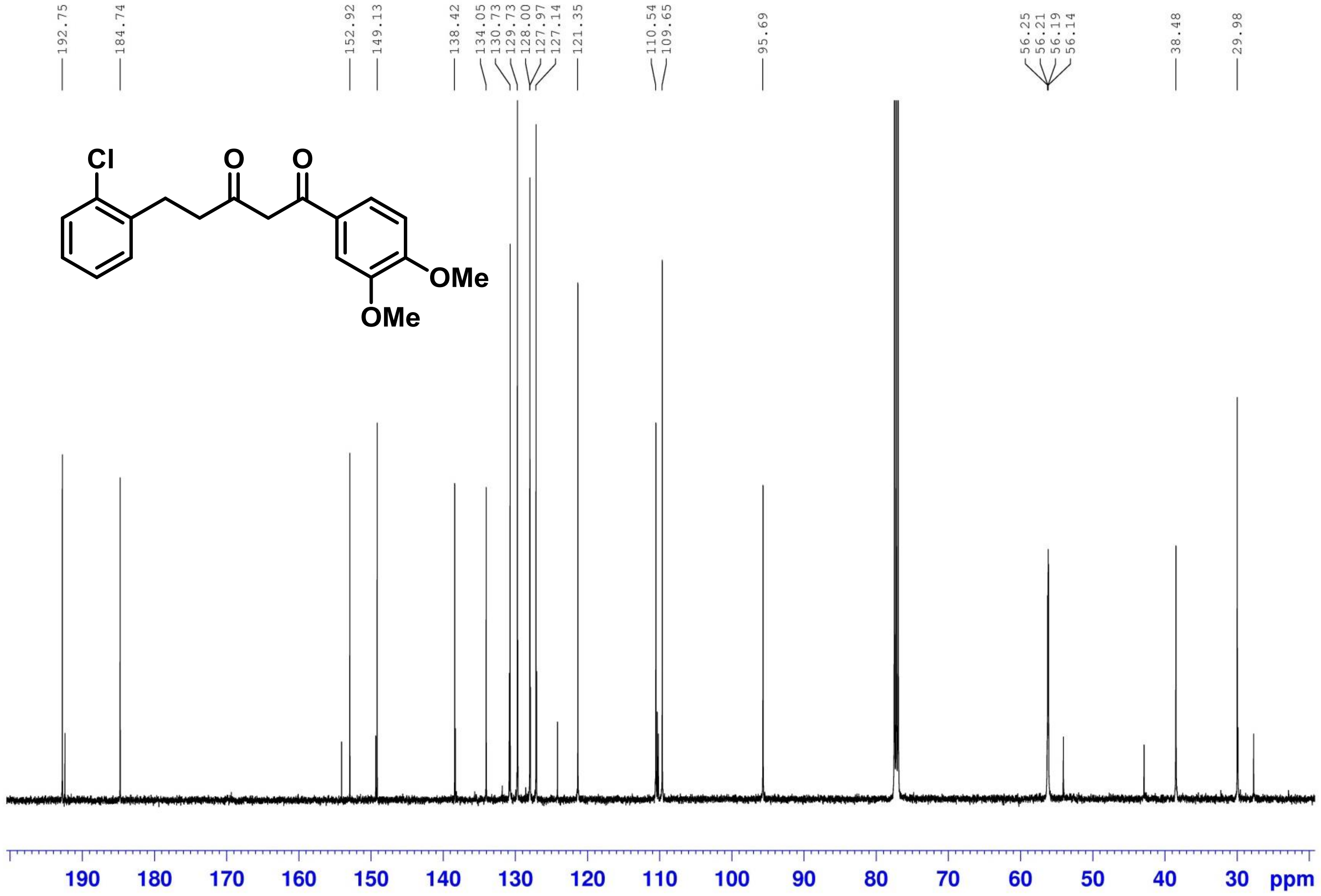

Figure S13: 400 MHz ^{1}H NMR spectrum of the compound **4g**.

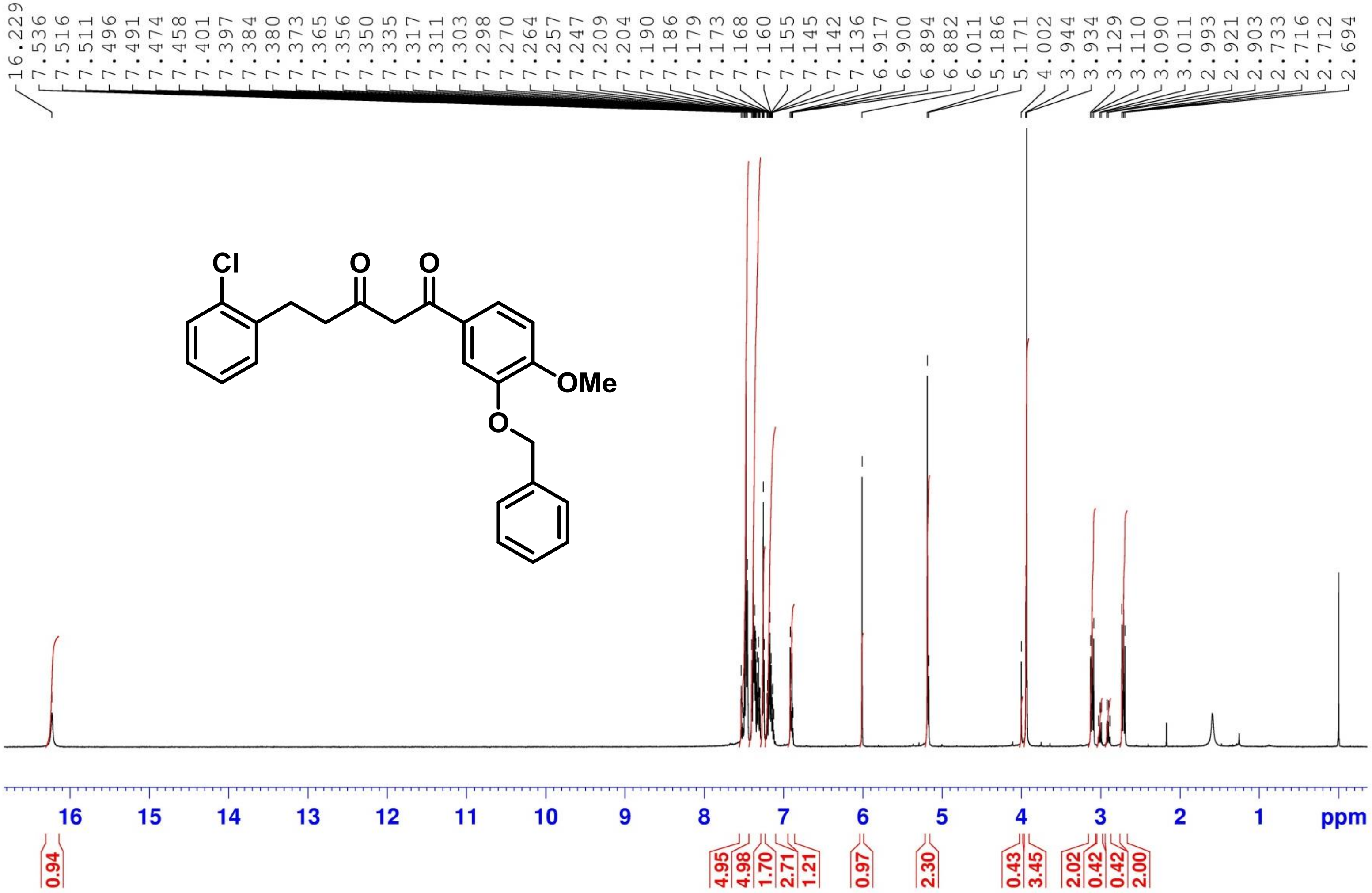

Figure S13a: 400 MHz ^{1}H NMR spectrum of the compound **4g**, a detail.

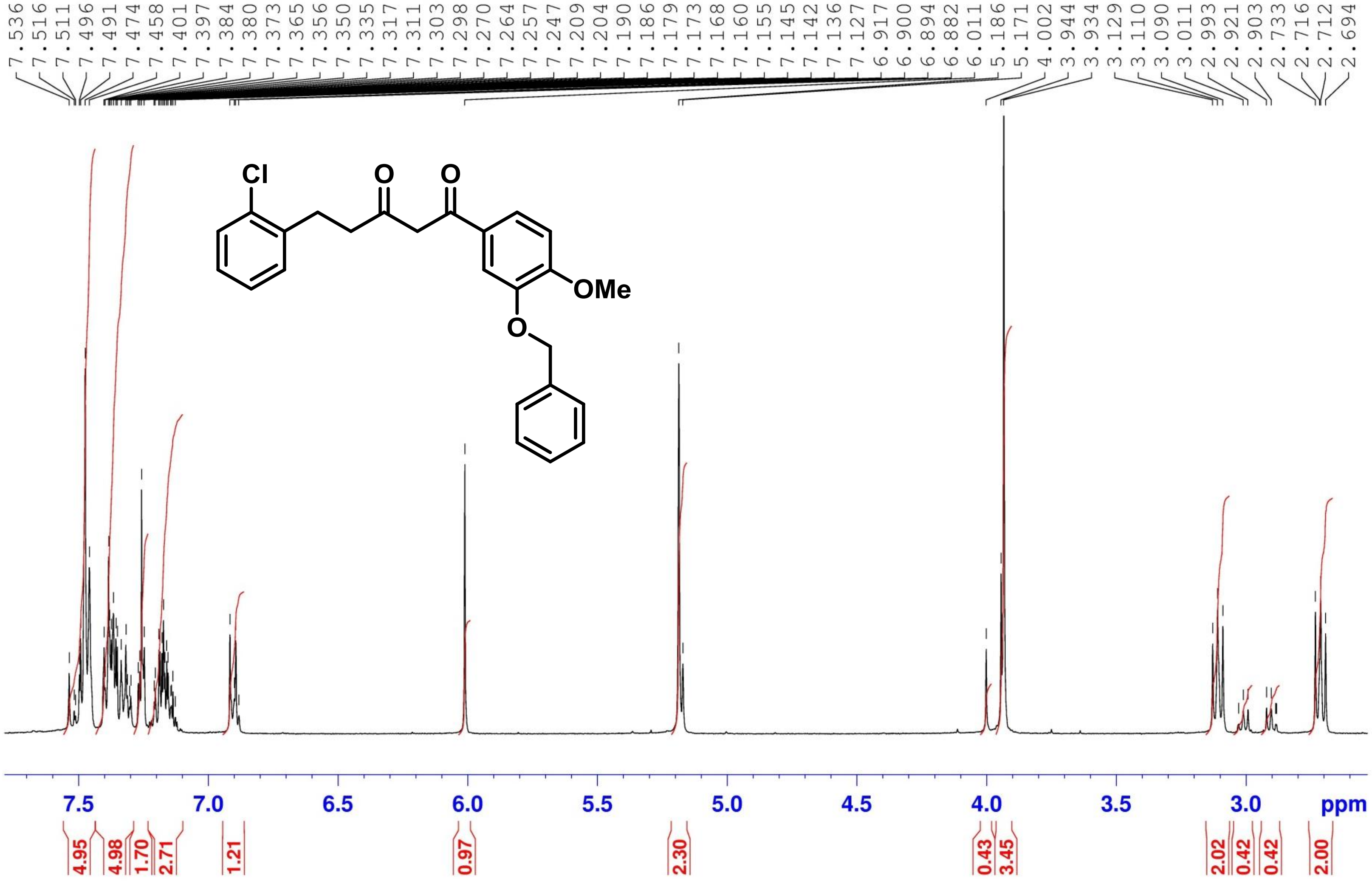

Figure S14: 100 MHz ^{13}C NMR spectrum of the compound **4g**.

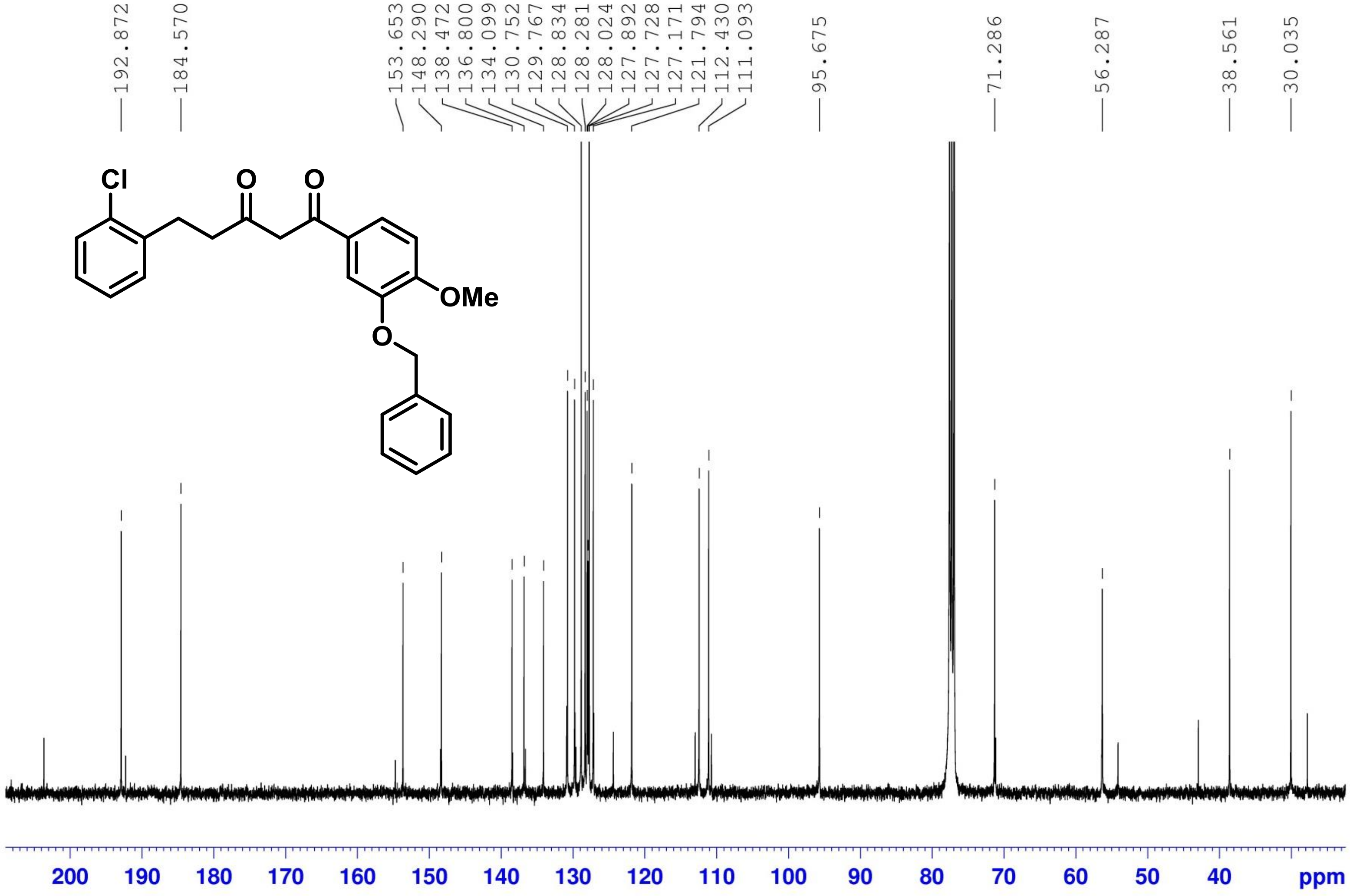

Figure S15: 400 MHz ^{1}H NMR spectrum of the compound **4h**.

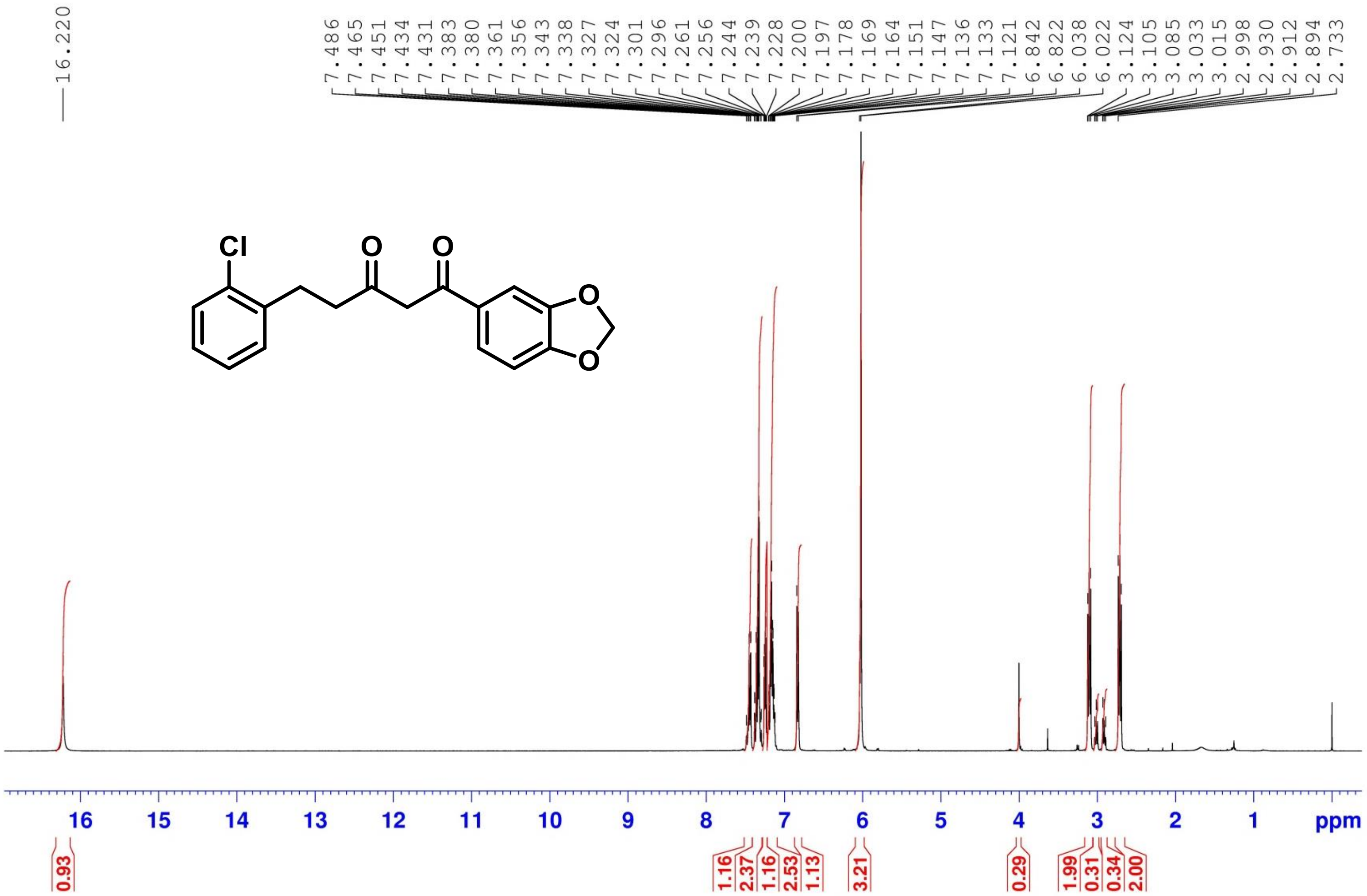

Figure S15a: 400 MHz ^{1}H NMR spectrum of the compound **4h**, a detail.

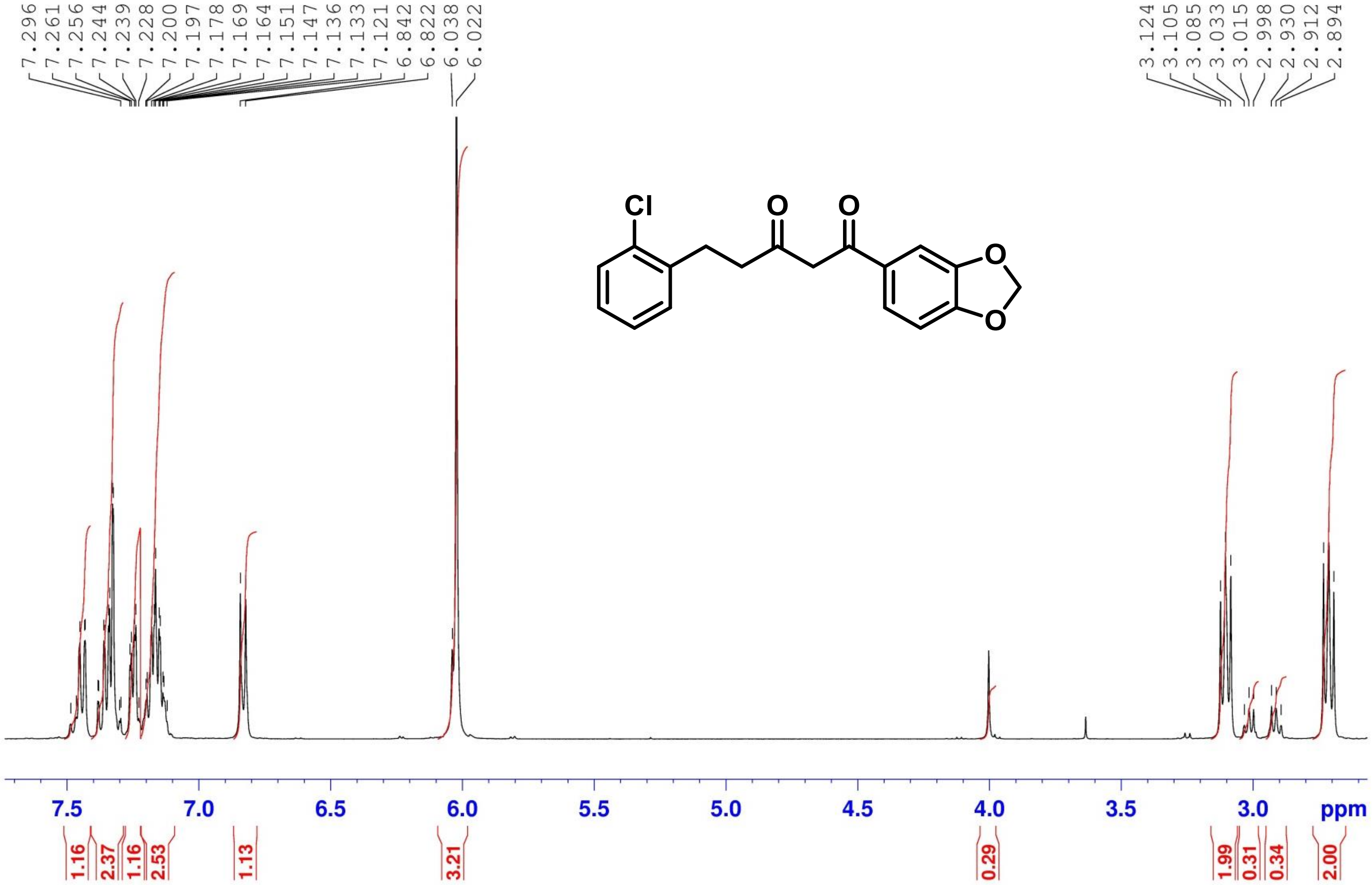

Figure S16: 100 MHz ^{13}C NMR spectrum of the compound **4h**.

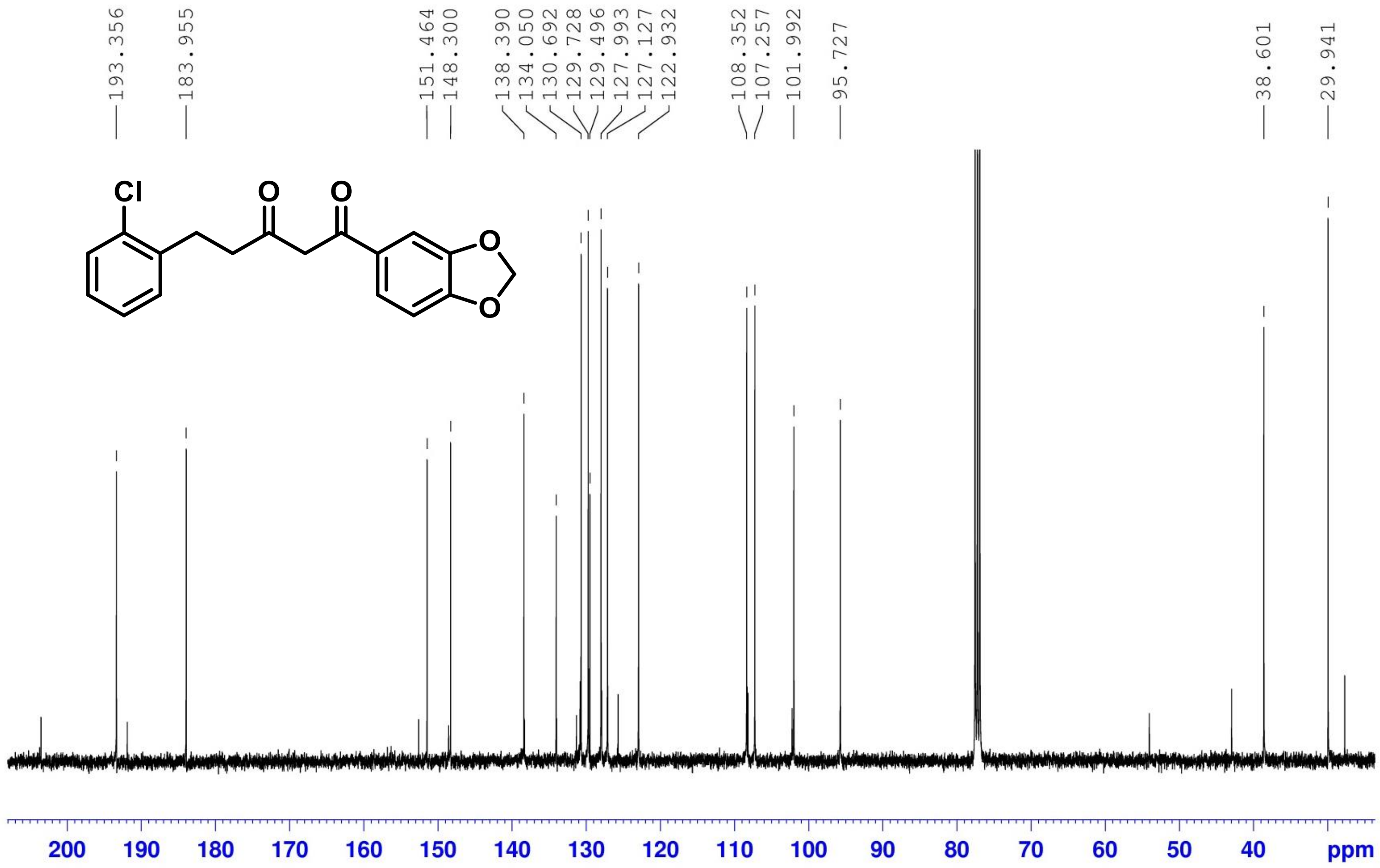

Figure S17: 400 MHz ^{1}H NMR spectrum of the compound **3a**.

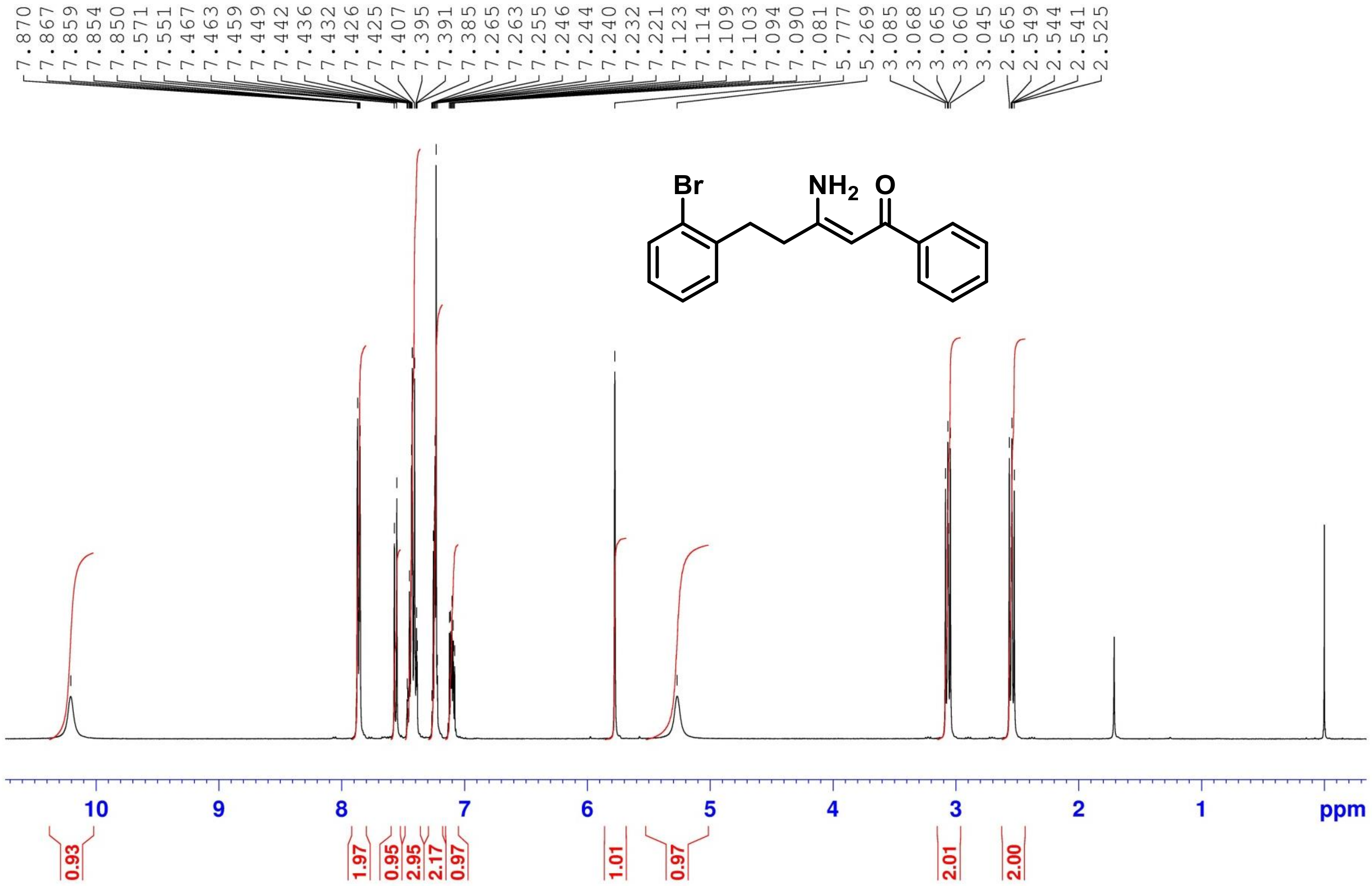

Figure S17a: 400 MHz ^{1}H NMR spectrum of the compound **3a**, a detail.

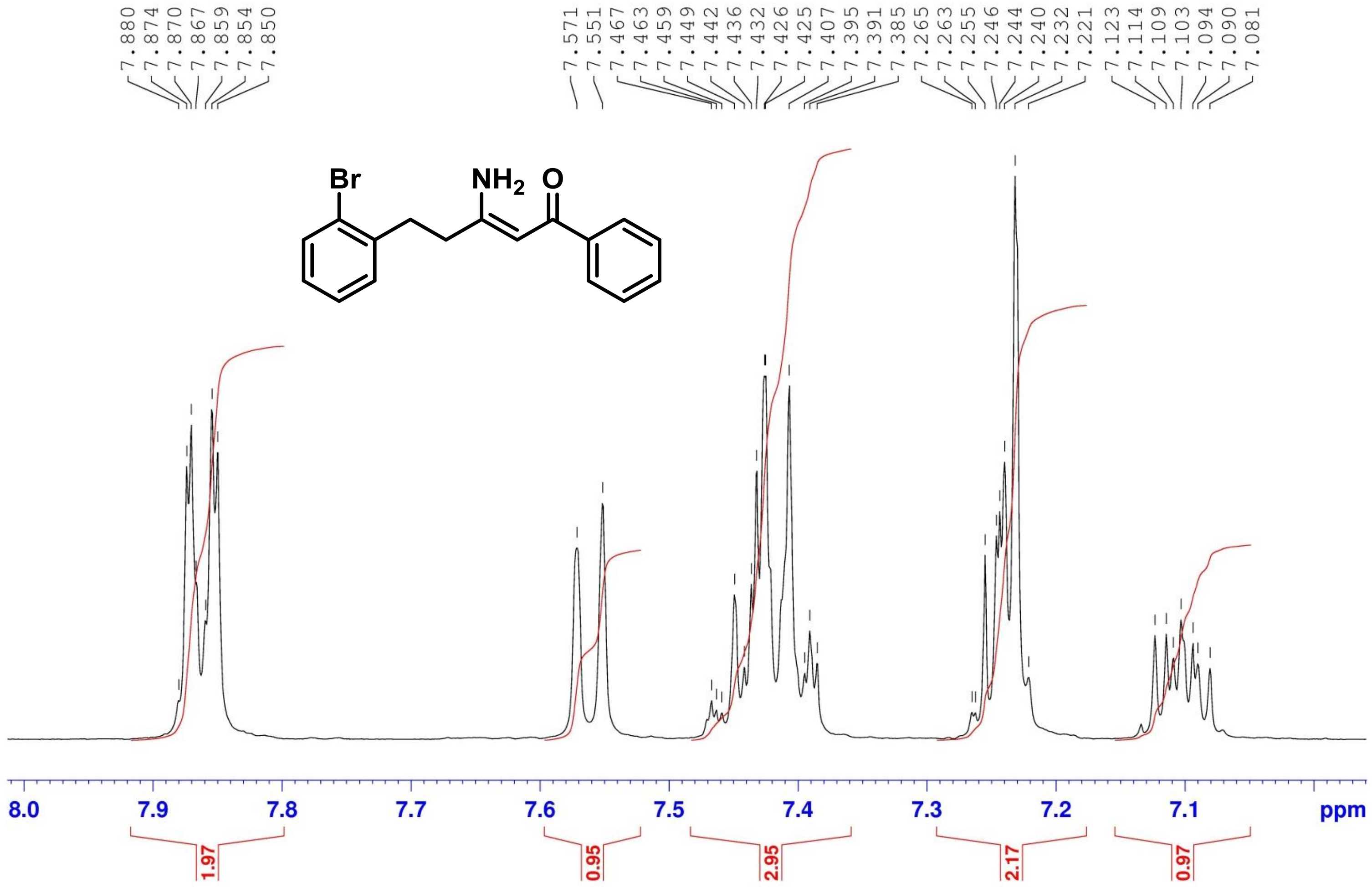

Figure S18: 100 MHz APT spectrum of the compound **3a**.

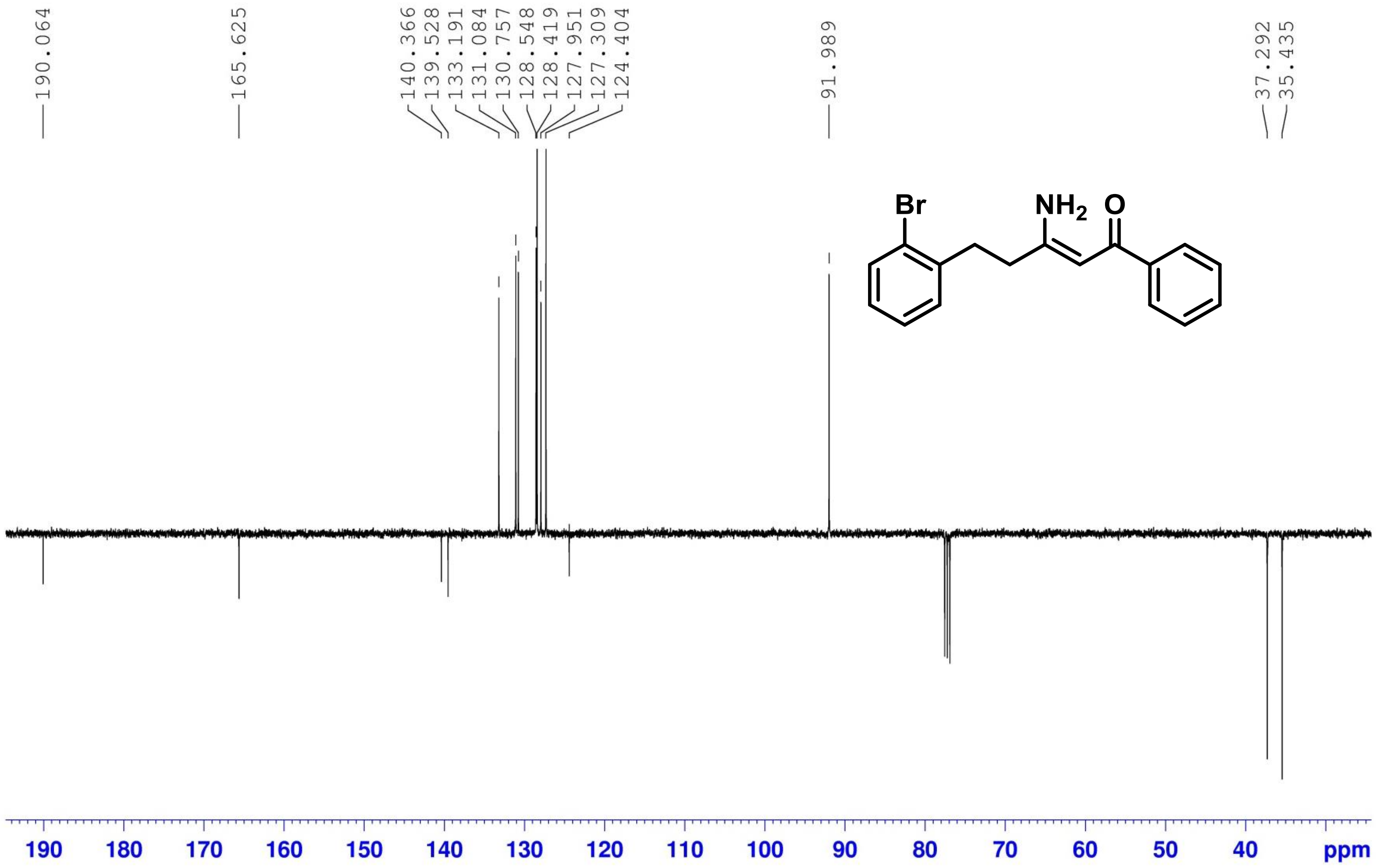

Figure S19: 400 MHz ^{1}H NMR spectrum of the compound **3b**.

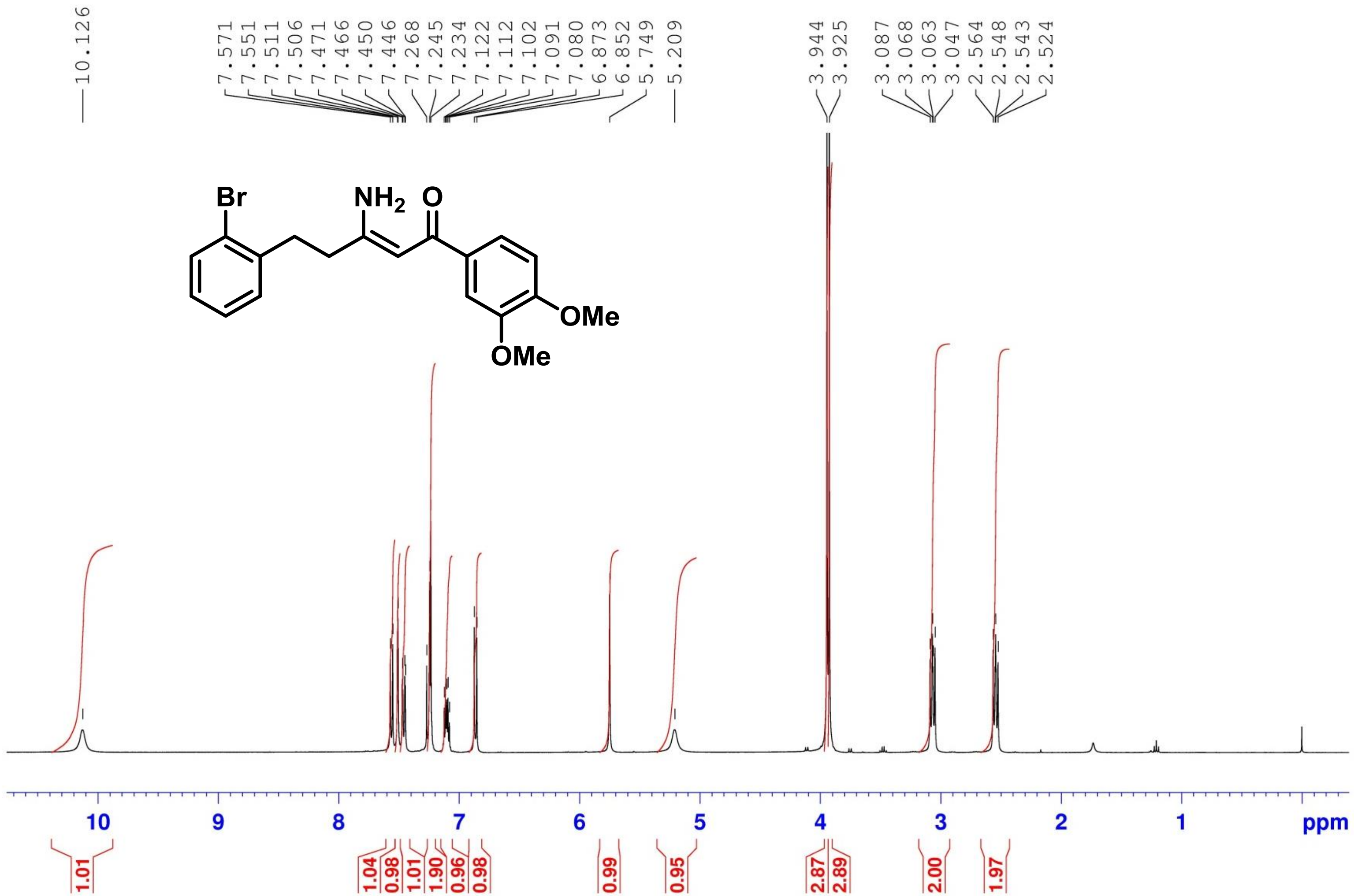

Figure S19a: 400 MHz ^{1}H NMR spectrum of the compound **3b**, a detail.

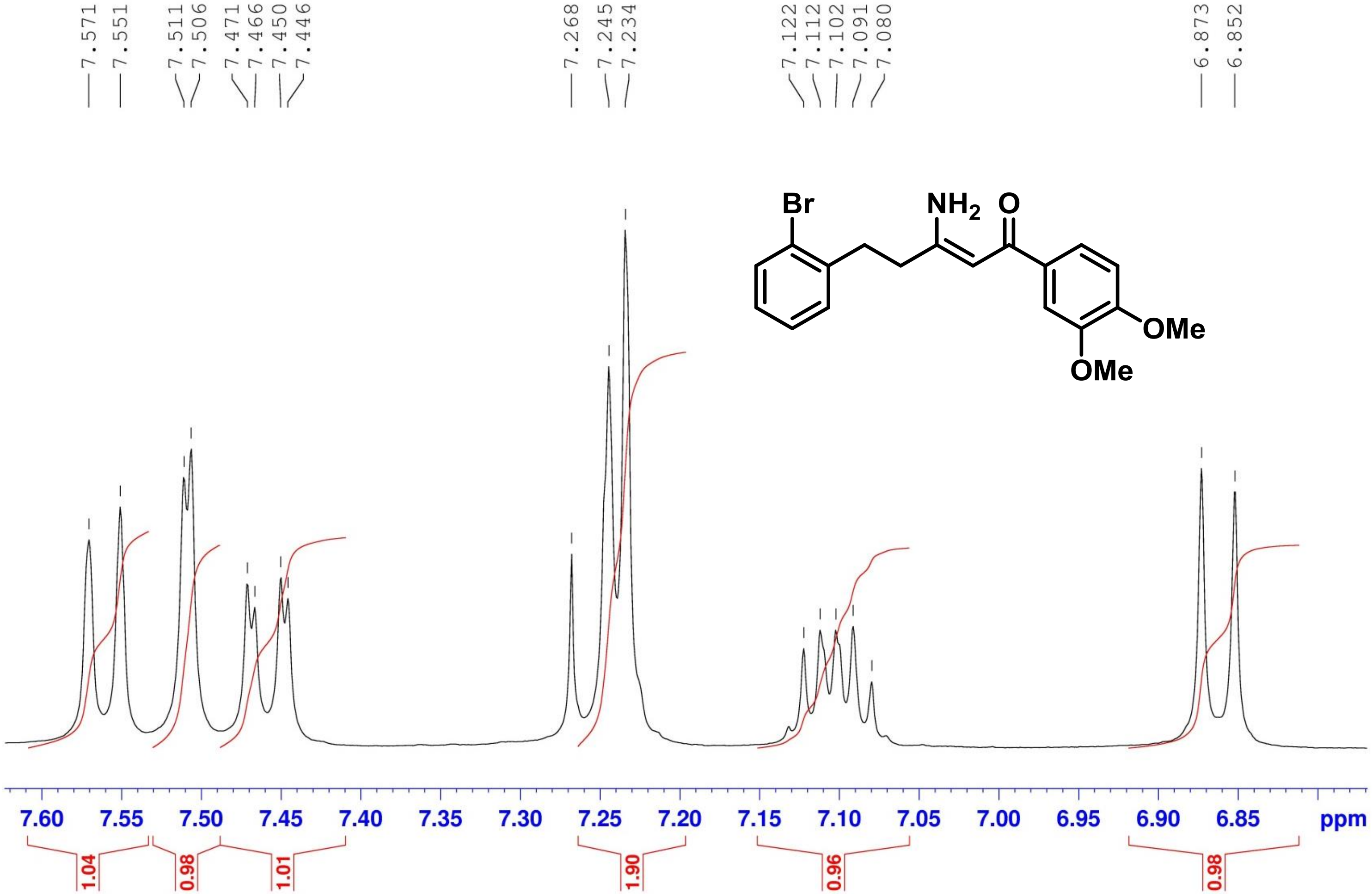

Figure S20: 100 MHz ^{13}C NMR spectrum of the compound **3b**.

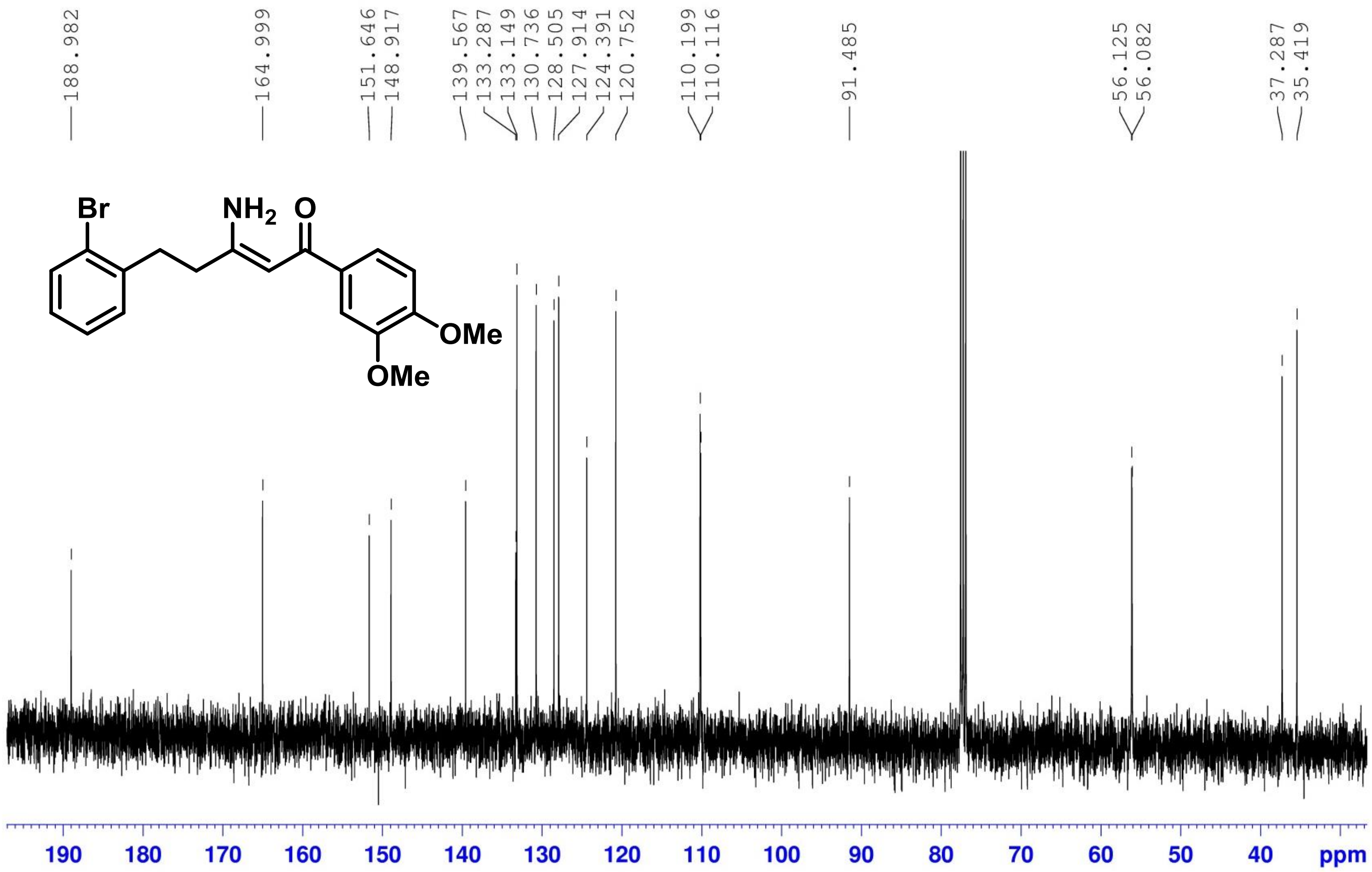

Figure S21: 400 MHz ^{1}H NMR spectrum of the compound **3c**.

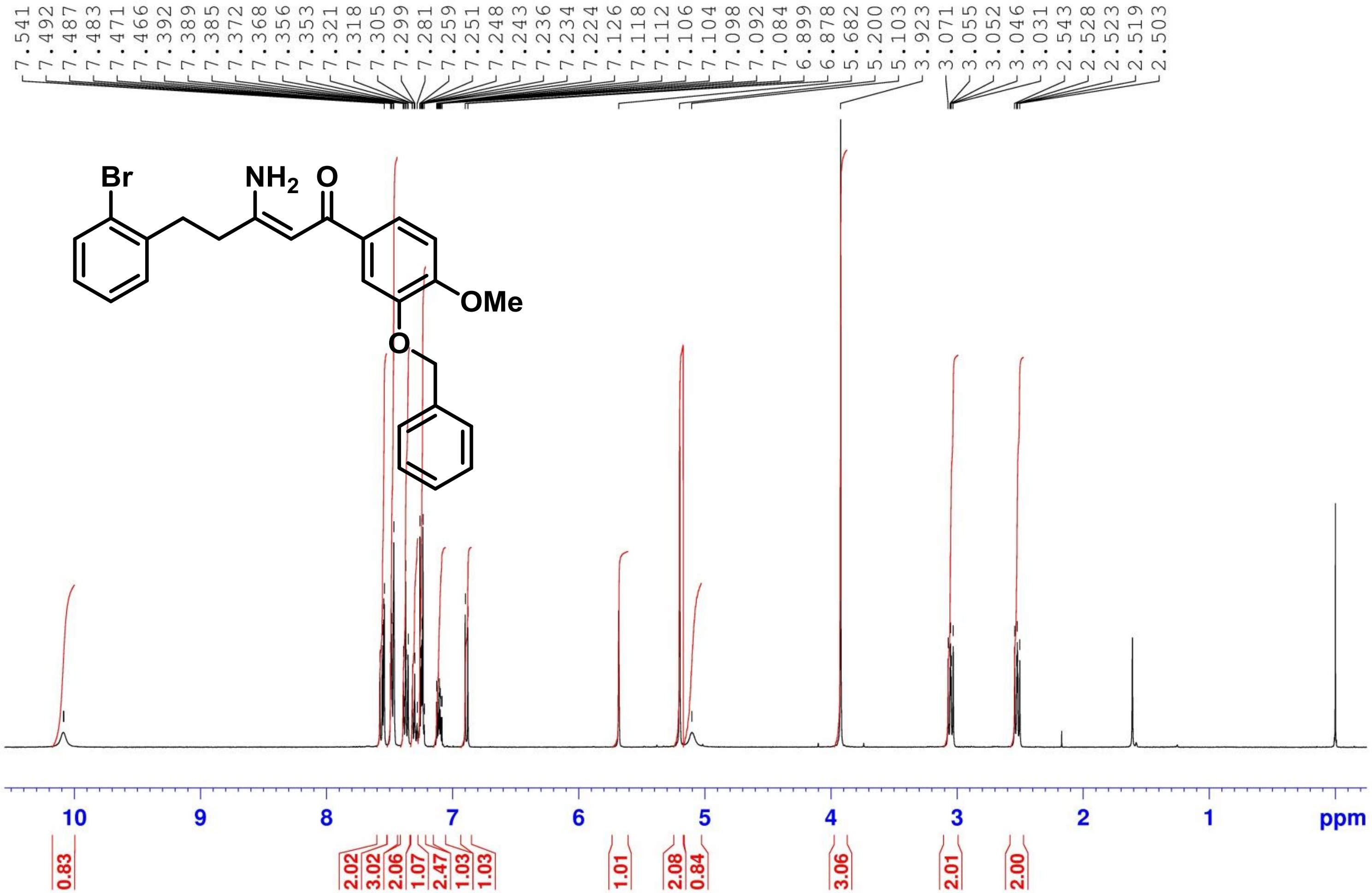

Figure S21a: 400 MHz ^{1}H NMR spectrum of the compound **3c**, a detail.

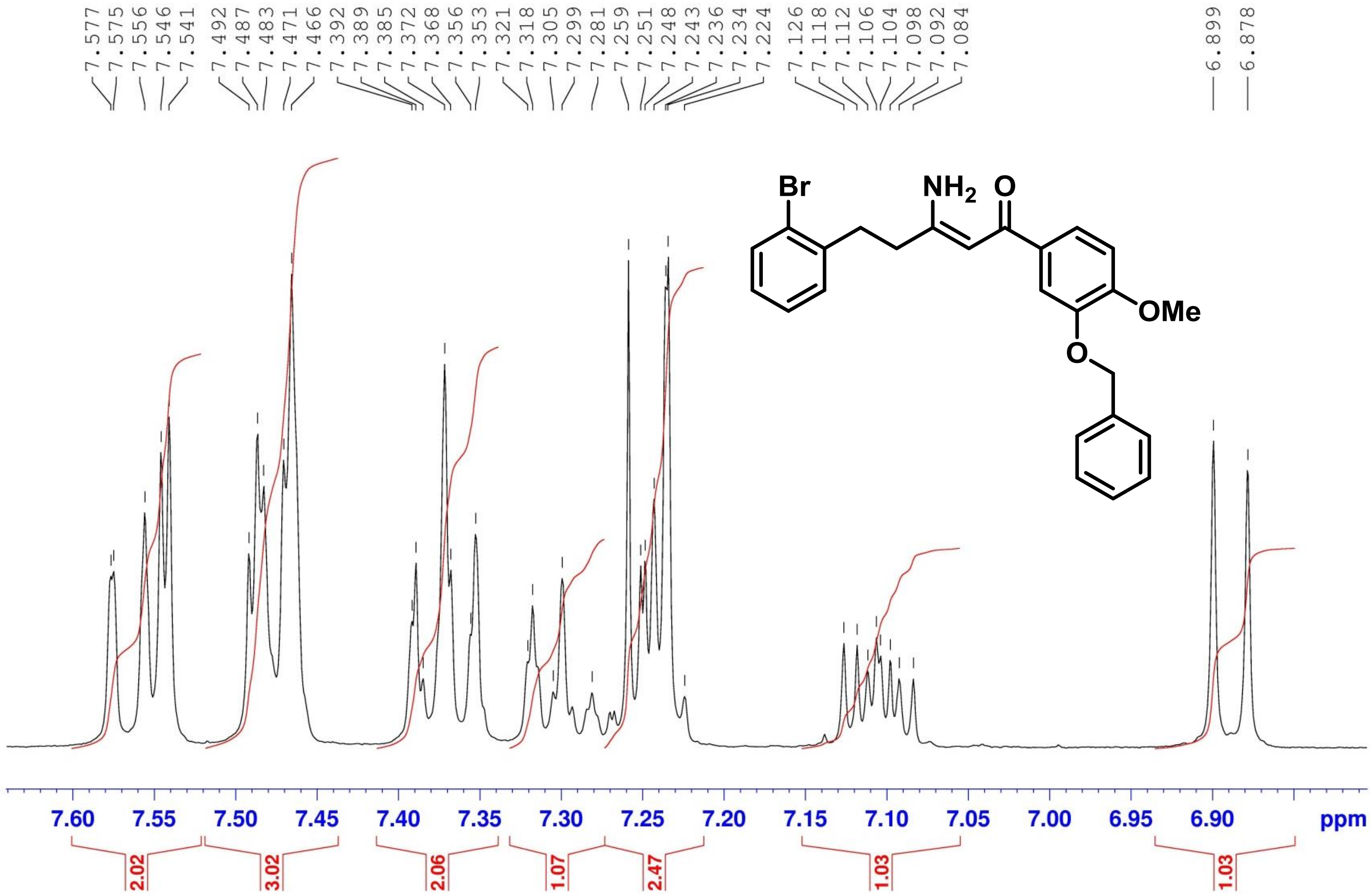

Figure S22: 100 MHz APT spectrum of the compound **3c**.

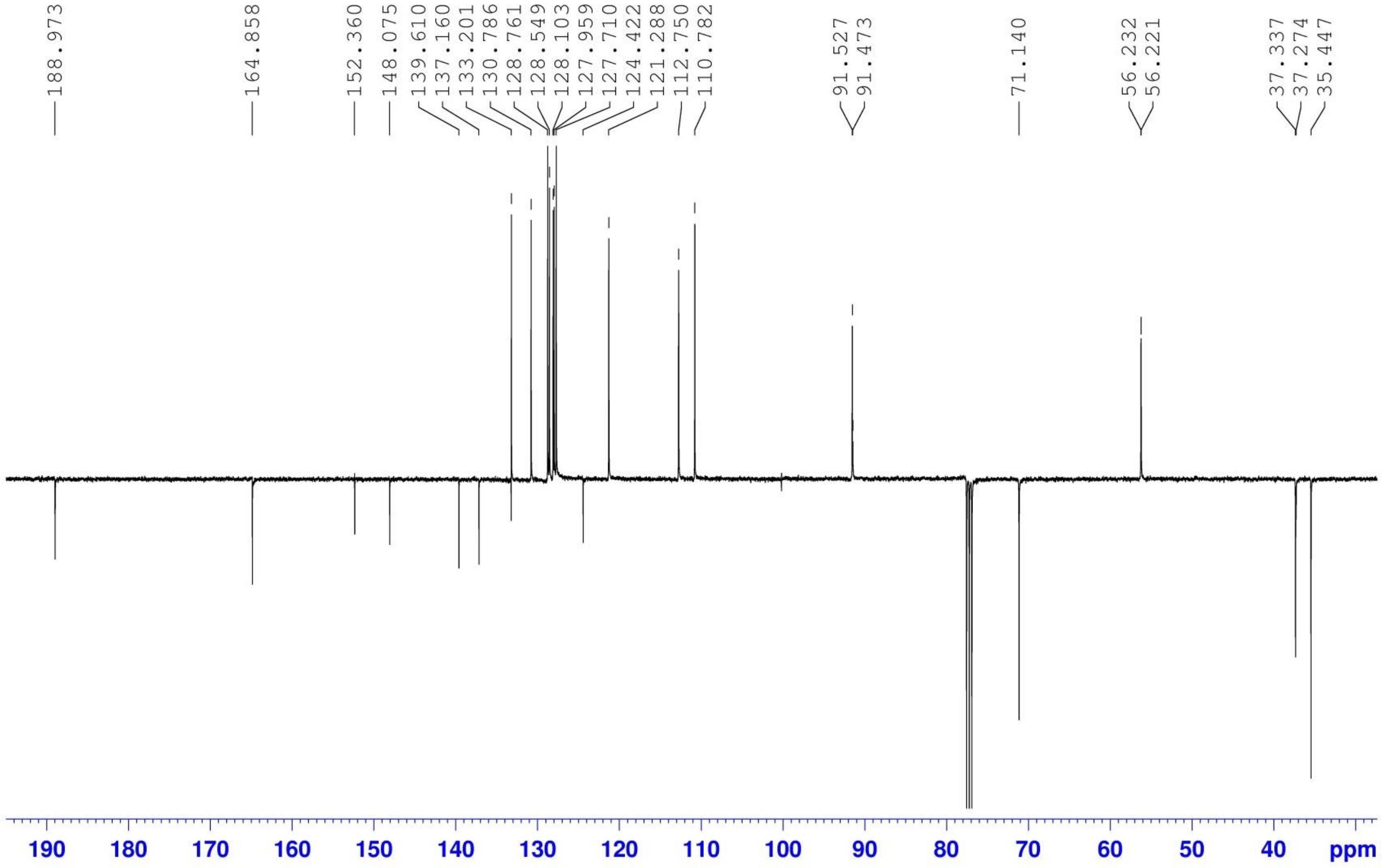

Figure S23: 400 MHz ^{1}H NMR spectrum of the compound **3d**.

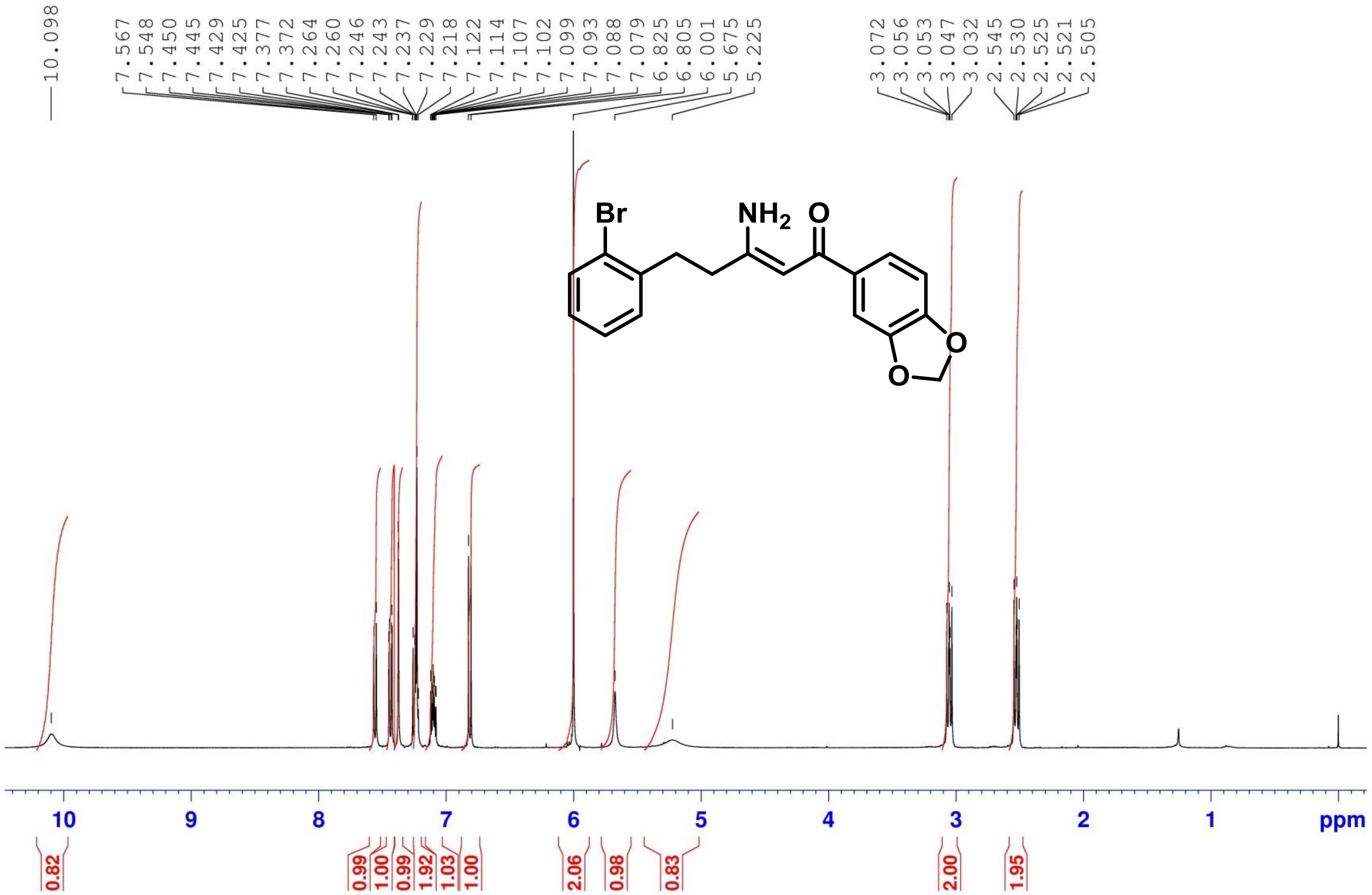

Figure S23a: 400 MHz ^{1}H NMR spectrum of the compound **3d**, a detail.

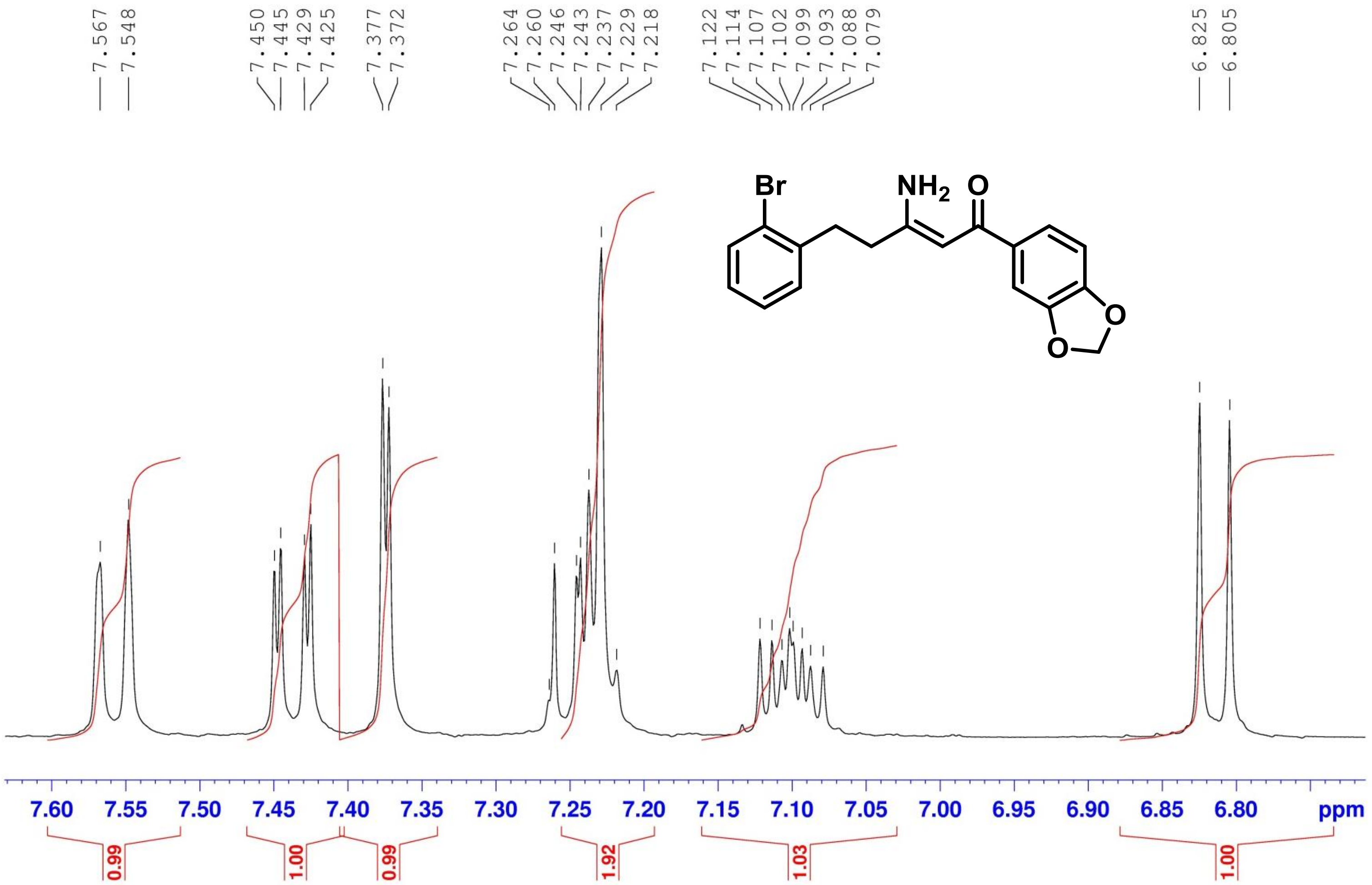

Figure S24: 100 MHz APT spectrum of the compound **3d**.

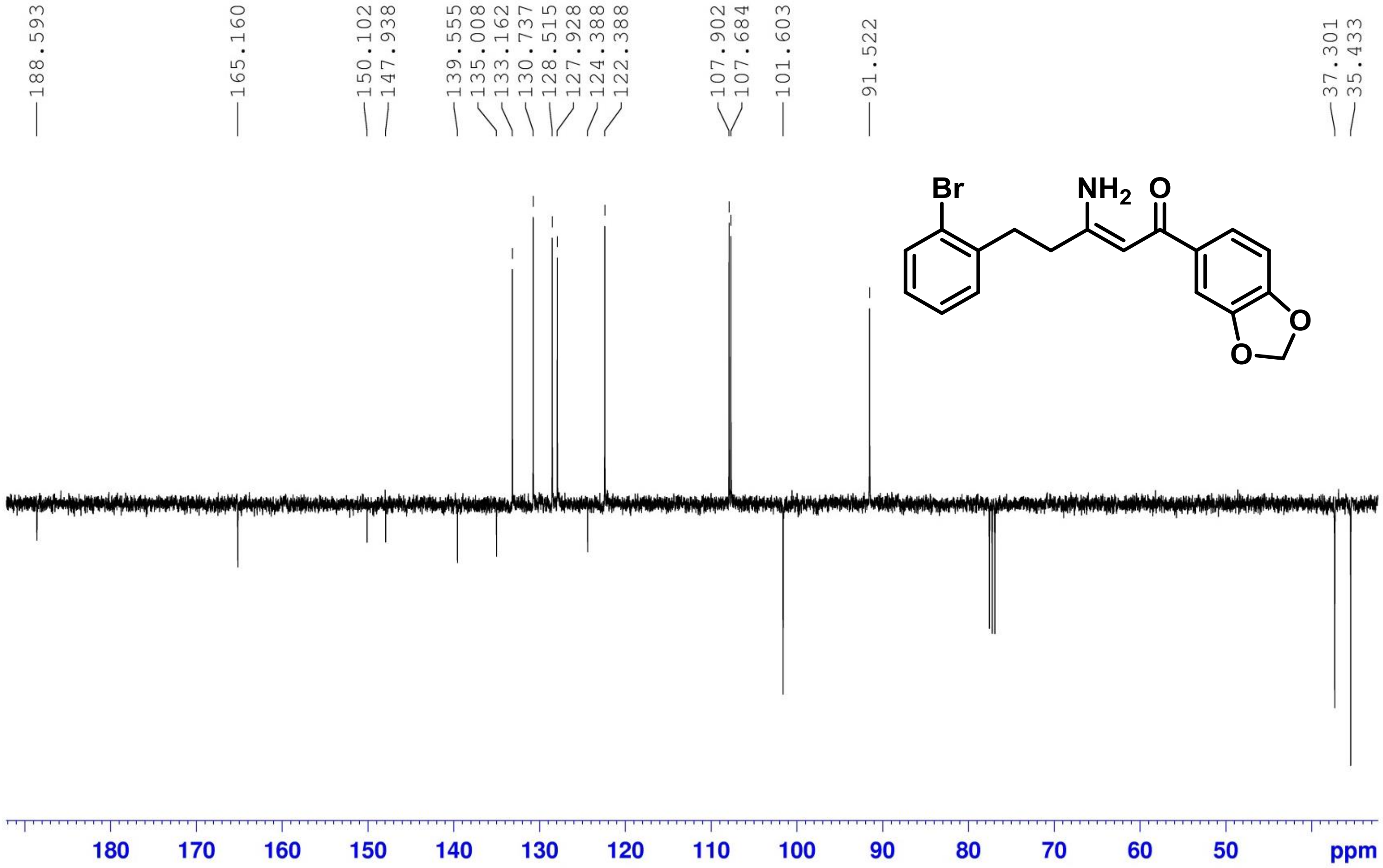

Figure S25: 400 MHz ^{1}H–^{13}C HMBC spectrum of the compound **3d**.

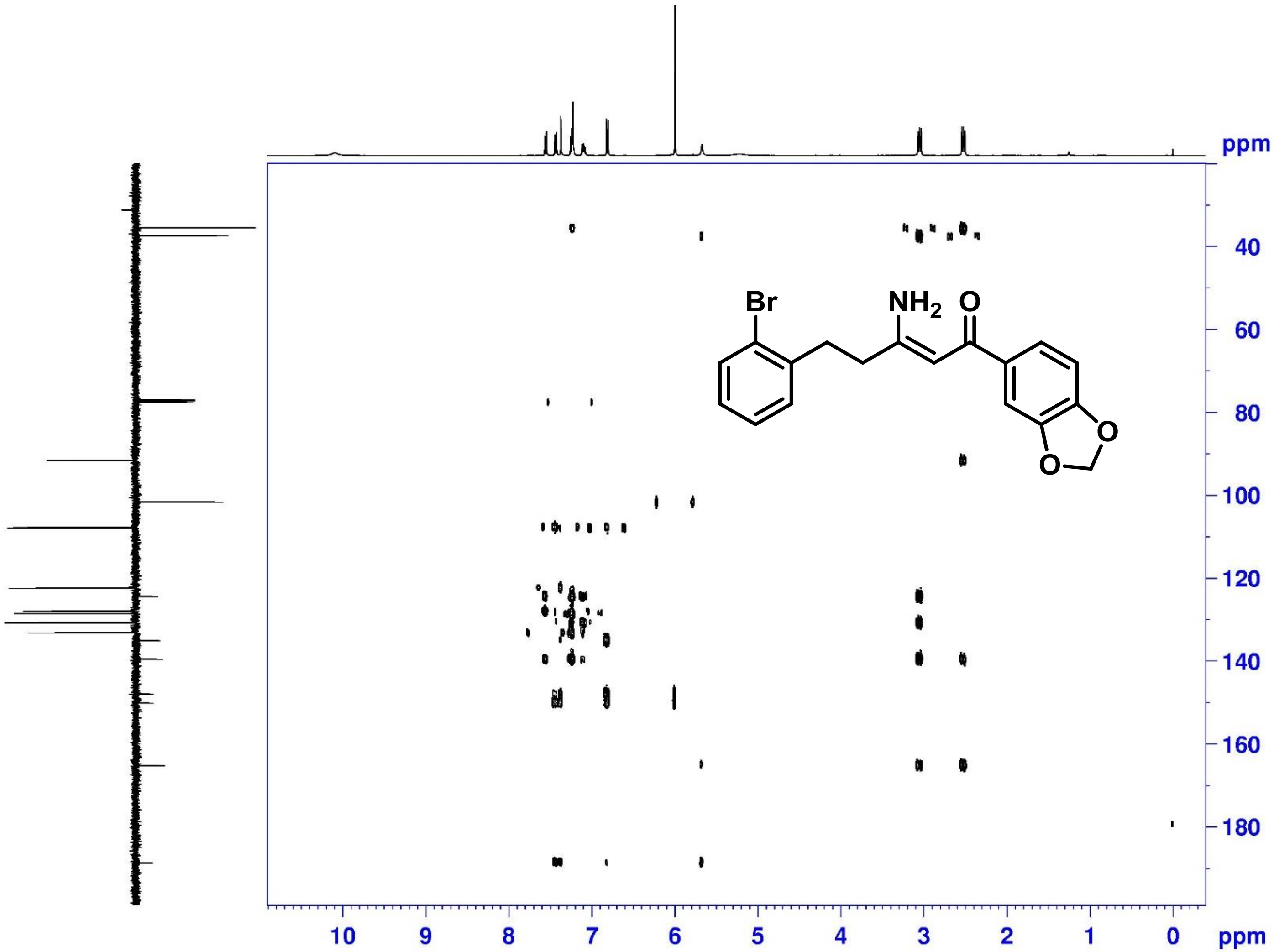

Figure S26: 400 MHz ^{1}H NMR spectrum of the compound **3e**.

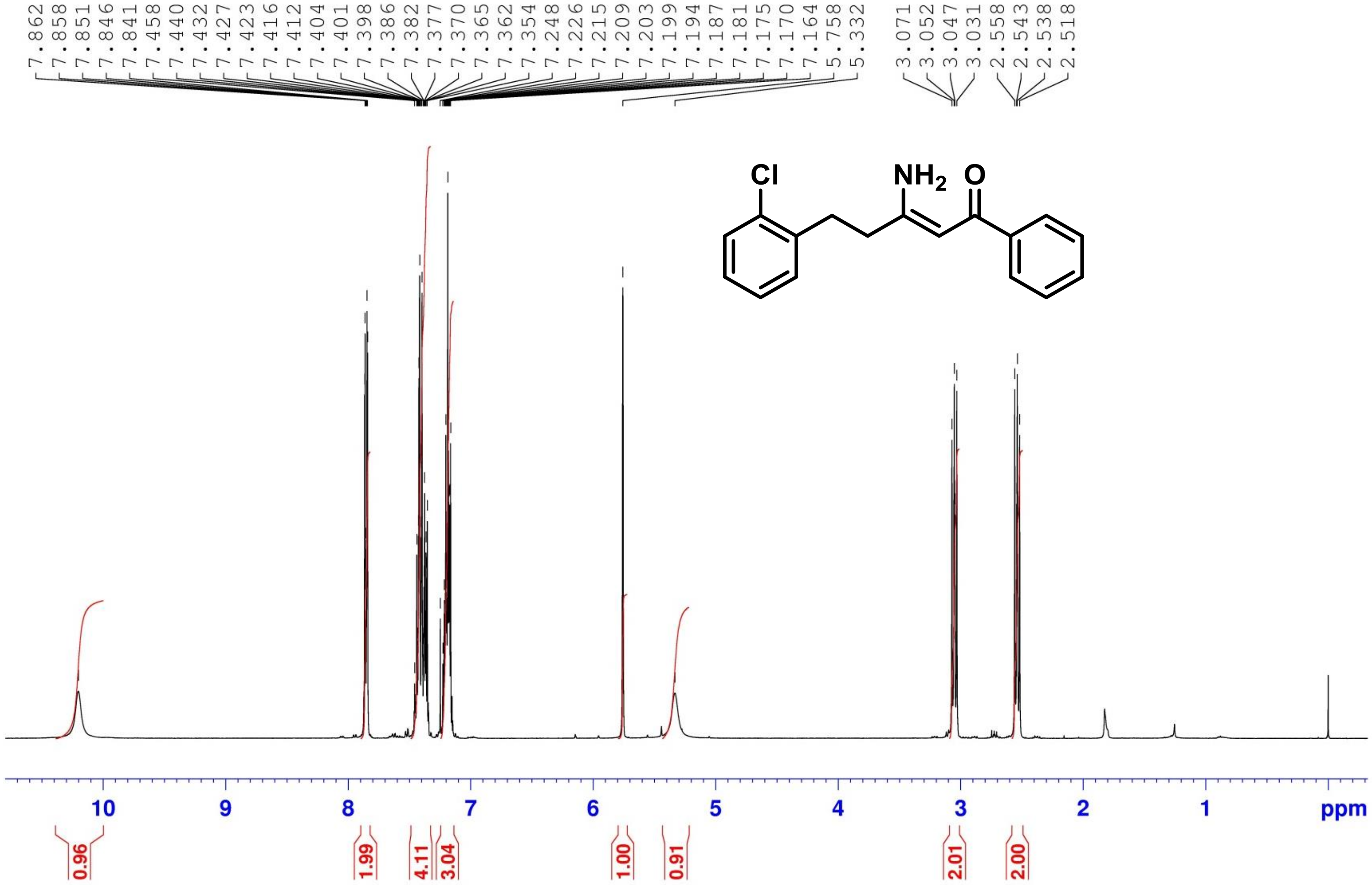

Figure S26a: 400 MHz ^{1}H NMR spectrum of the compound **3e**, a detail.

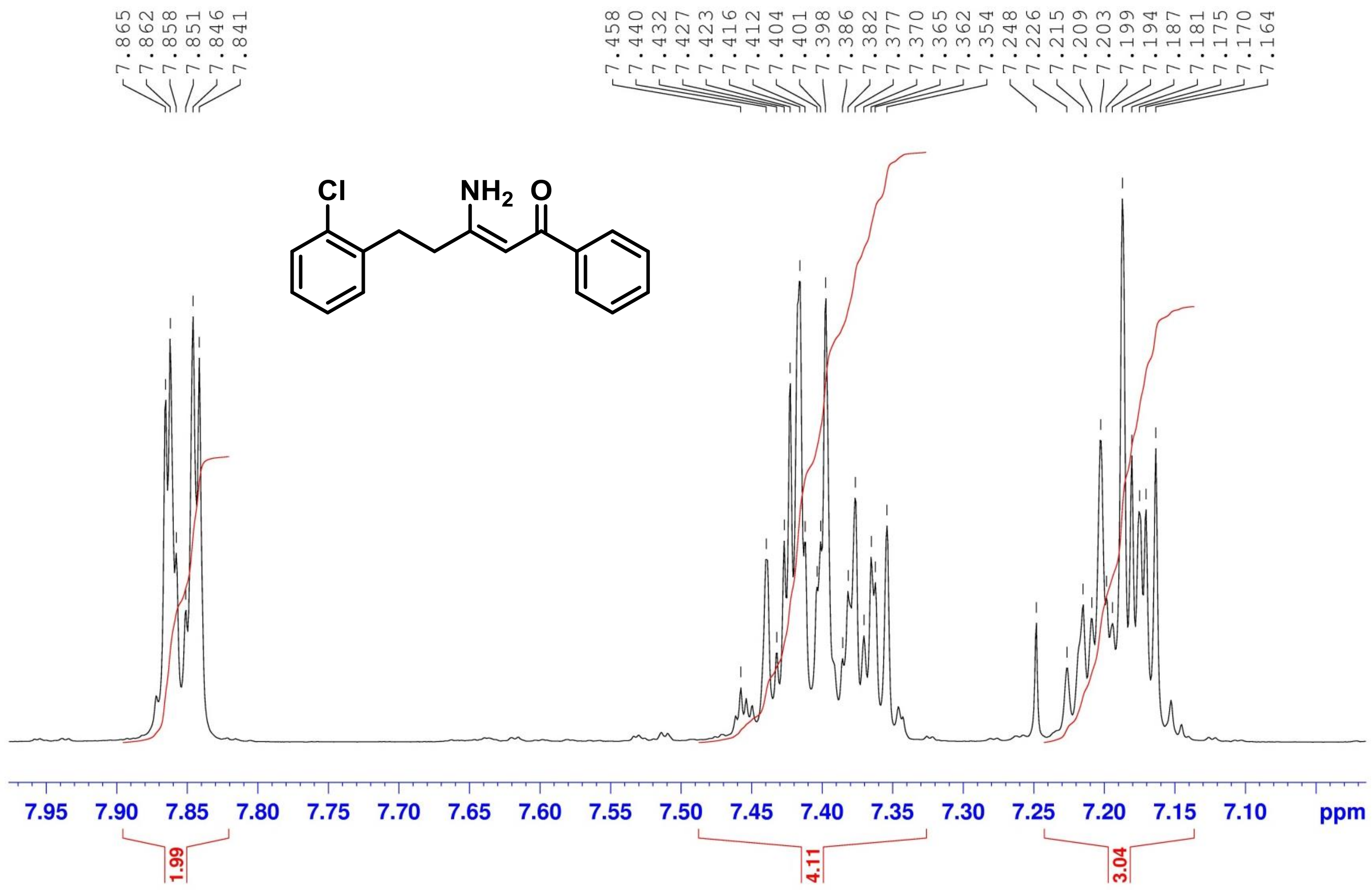

Figure S27: 100 MHz ^{13}C NMR spectrum of the compound **3e**.

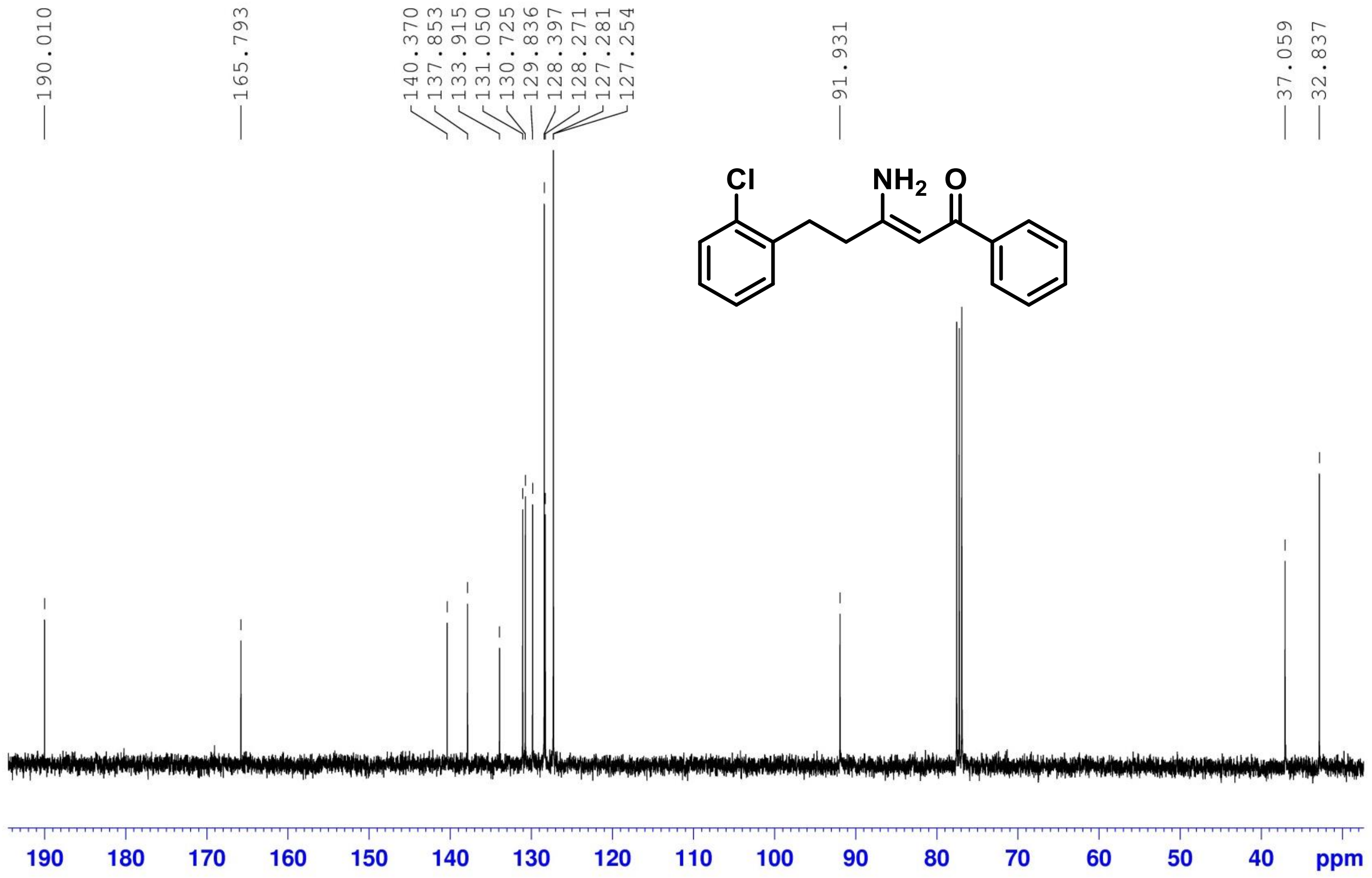

Figure S28: 400 MHz ^{1}H NMR spectrum of the compound 3**f**.

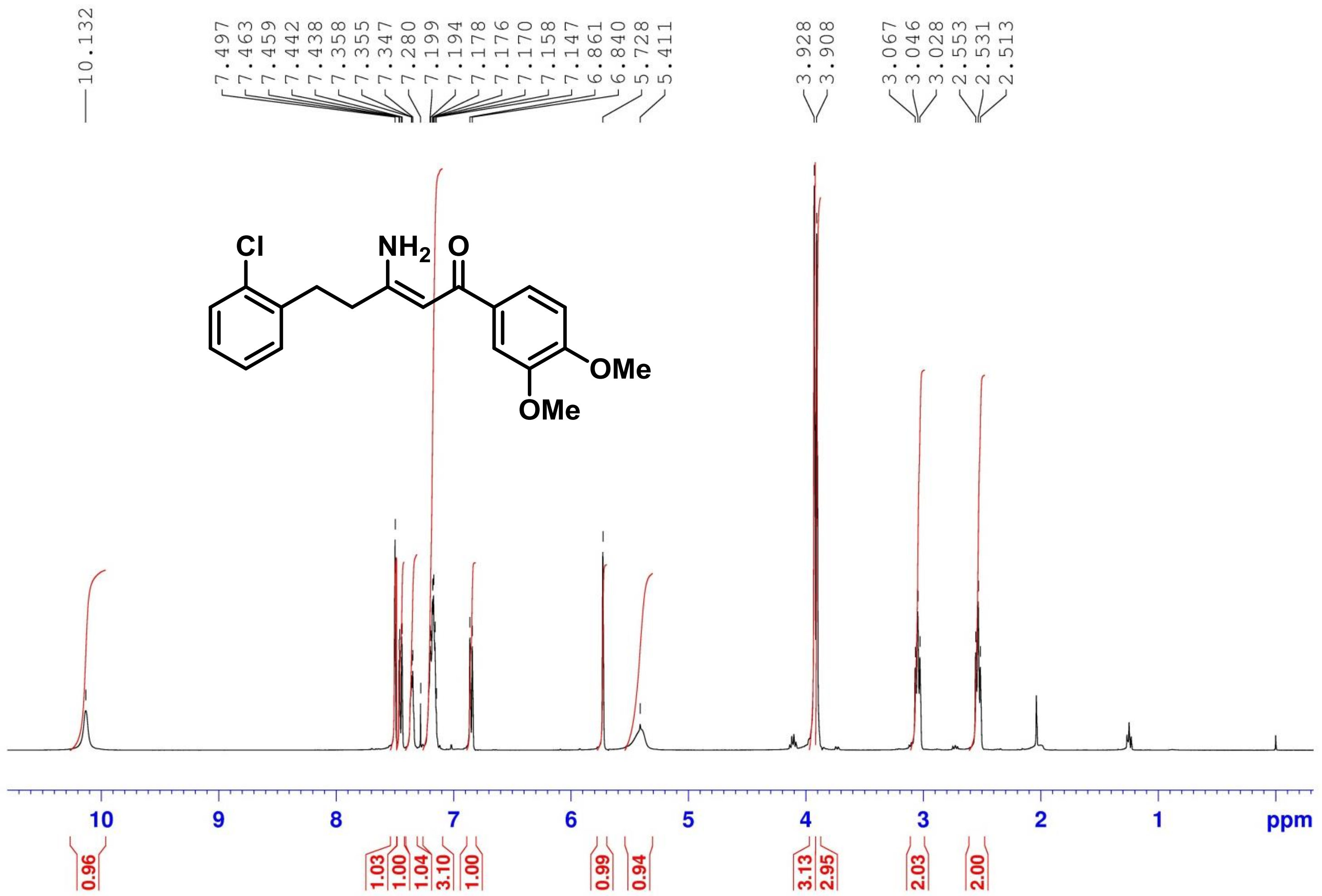

Figure S29: 100 MHz ^{13}C NMR spectrum of the compound **3f**.

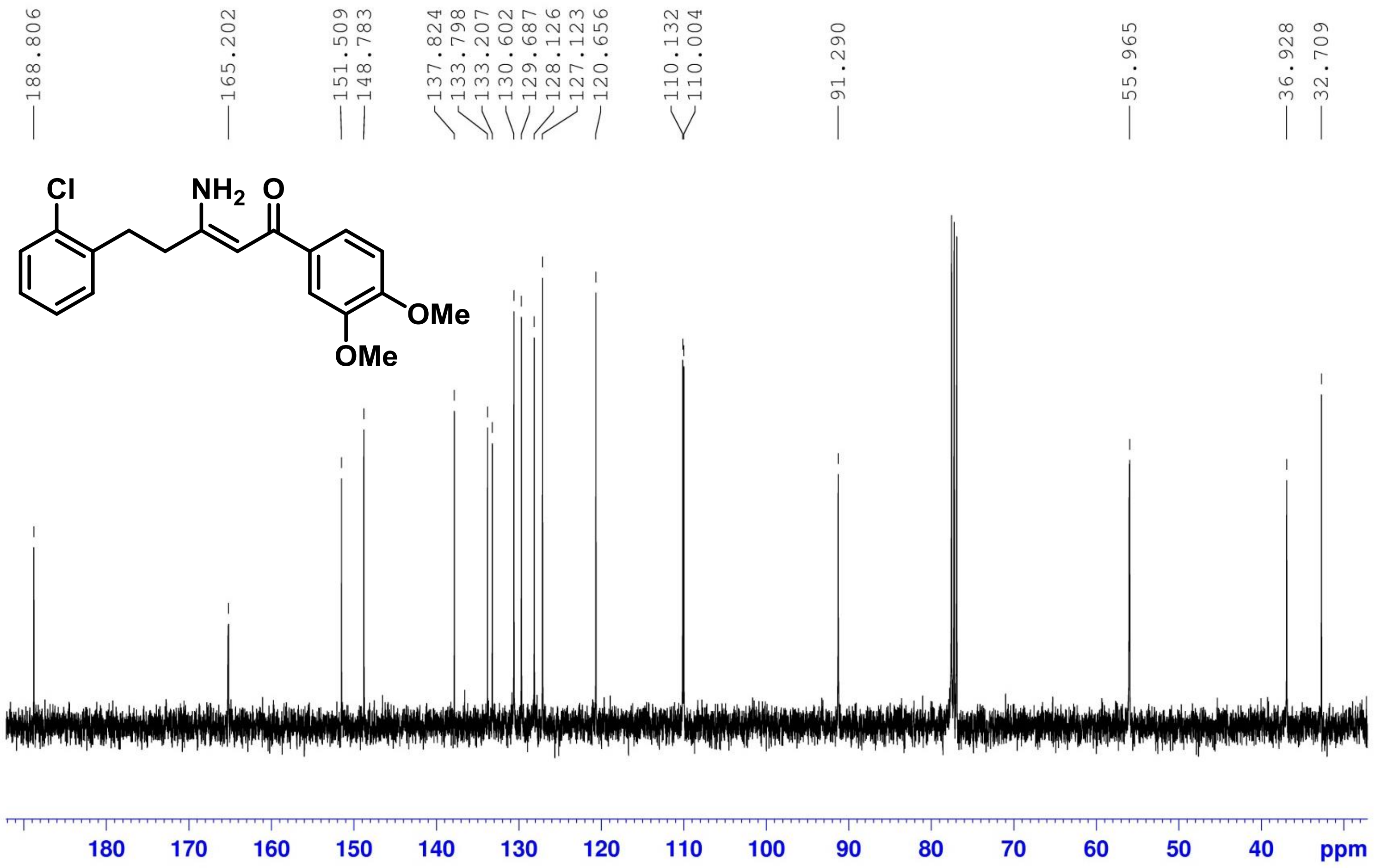

Figure S30: 400 MHz ^{1}H NMR spectrum of the compound **3g**.

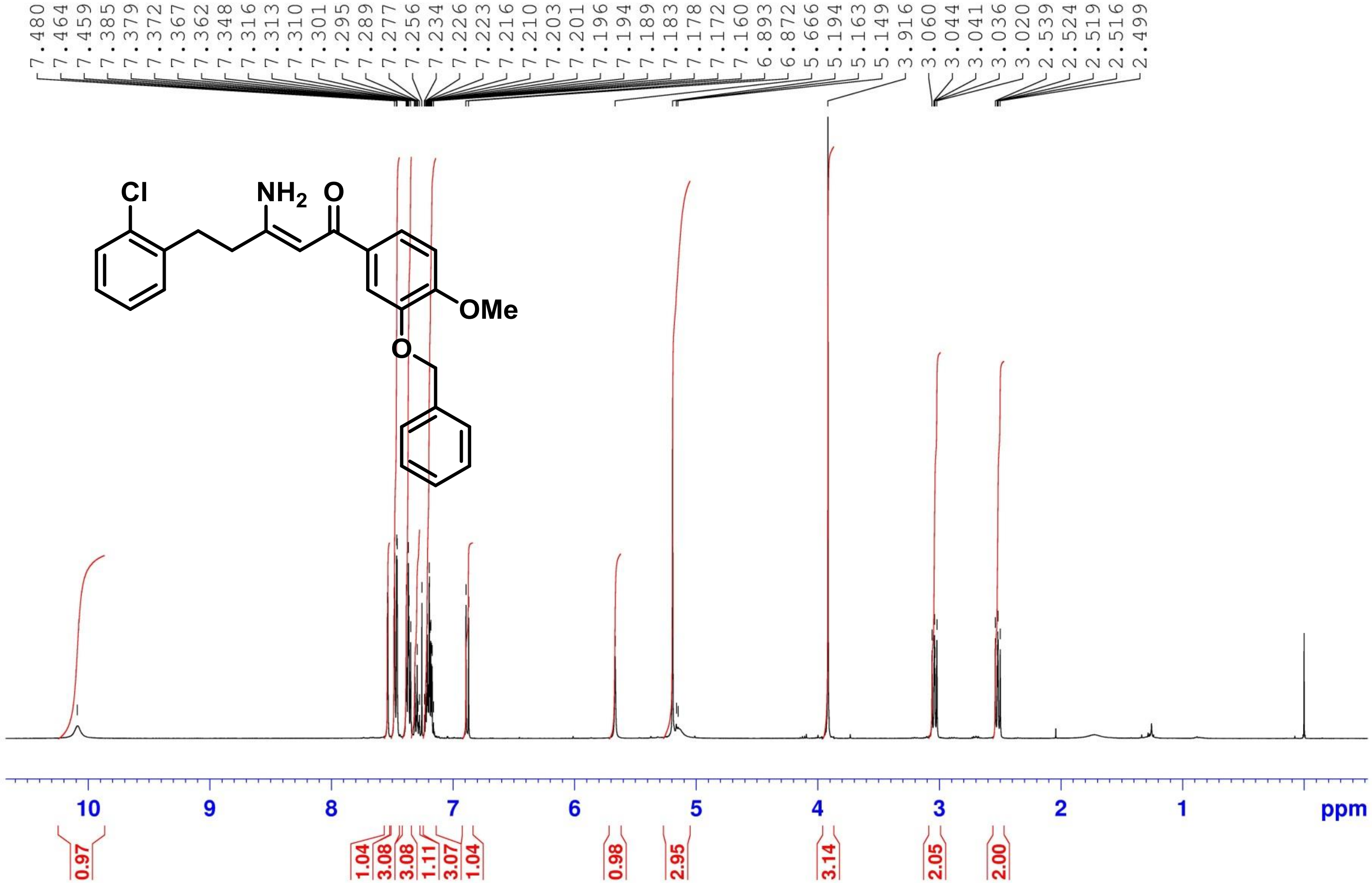

Figure S30a: 400 MHz ^{1}H NMR spectrum of the compound **3g**, a detail.

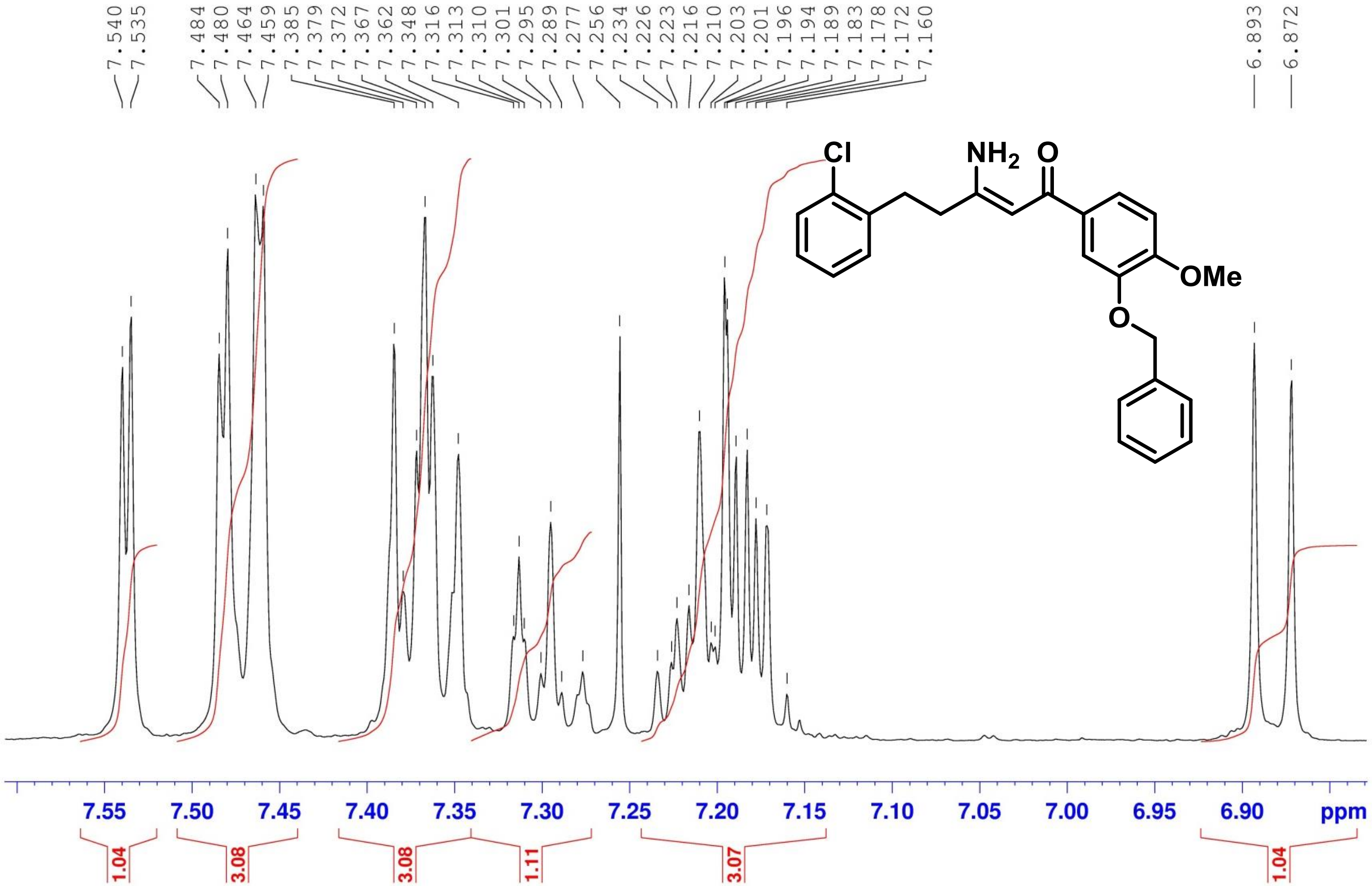

Figure S31: 100 MHz APT spectrum of the compound **3g**.

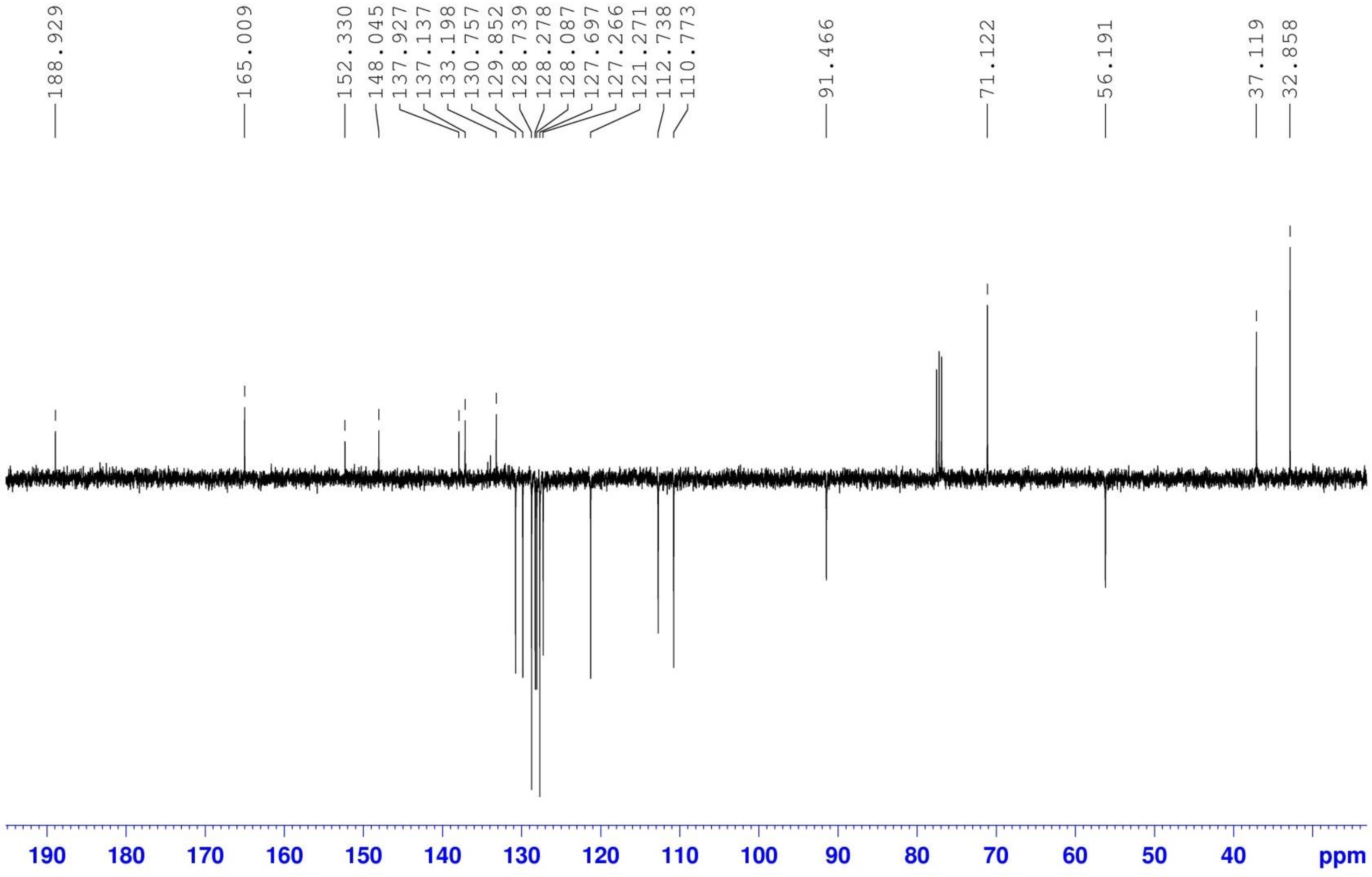

Figure S32: 400 MHz ^{1}H NMR spectrum of the compound 3**h**.

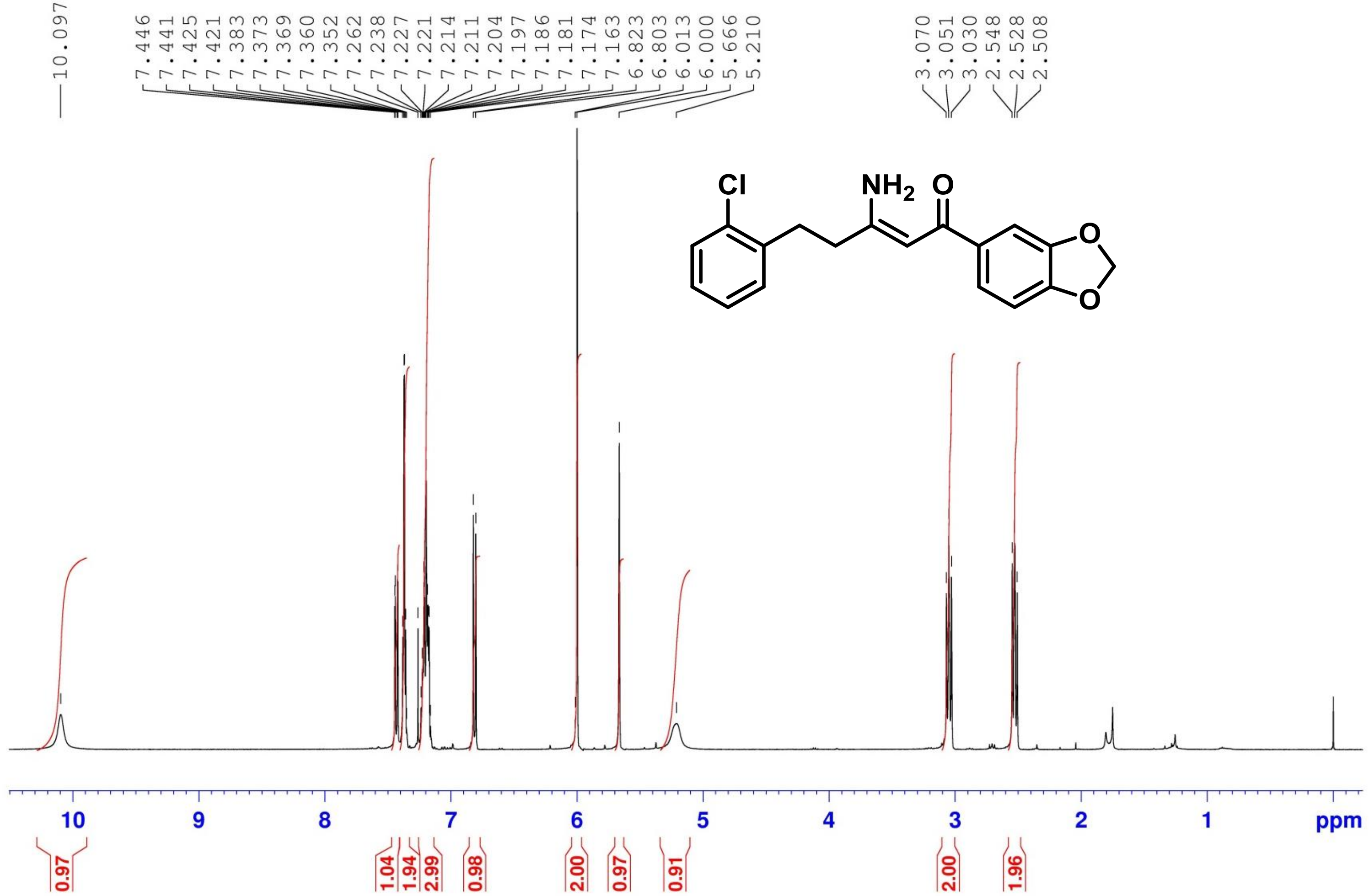

Figure S32a: 400 MHz ^{1}H NMR spectrum of the compound **3h**, a detail.

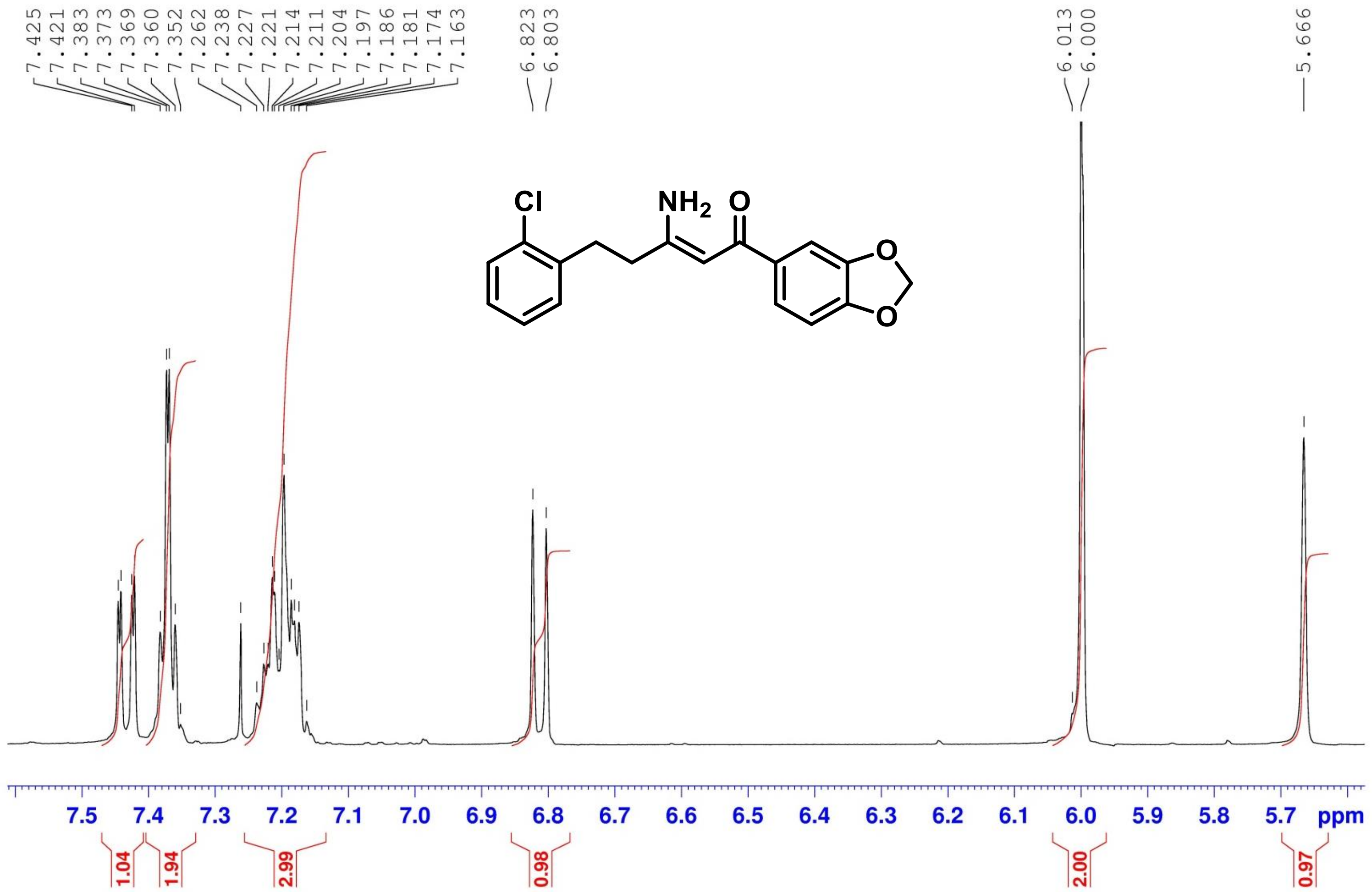

Figure S33: 400 MHz ^{1}H NMR spectrum of the compound **1a**.

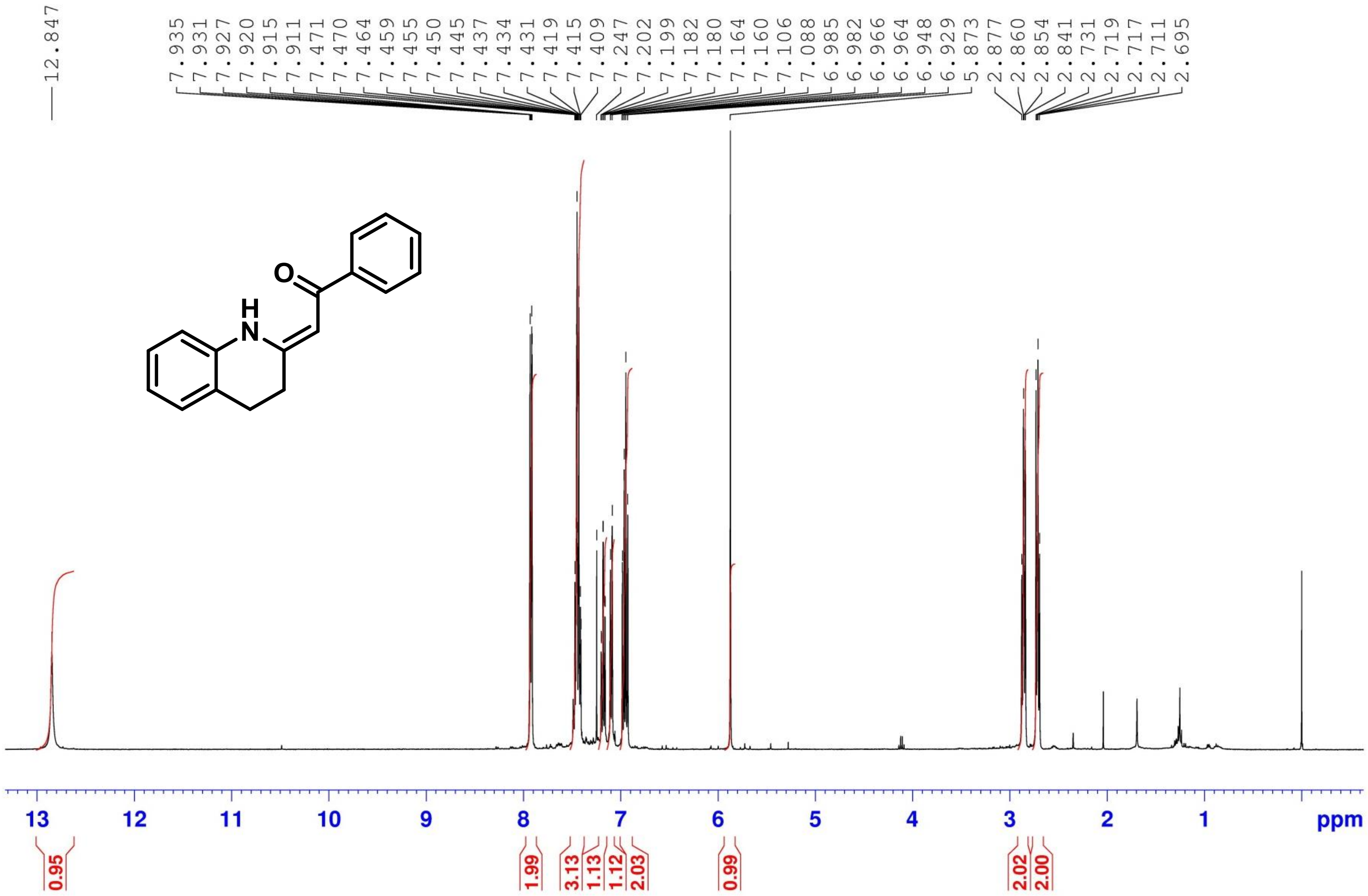

Figure S33a: 400 MHz ^{1}H NMR spectrum of the compound **1a**, a detail.

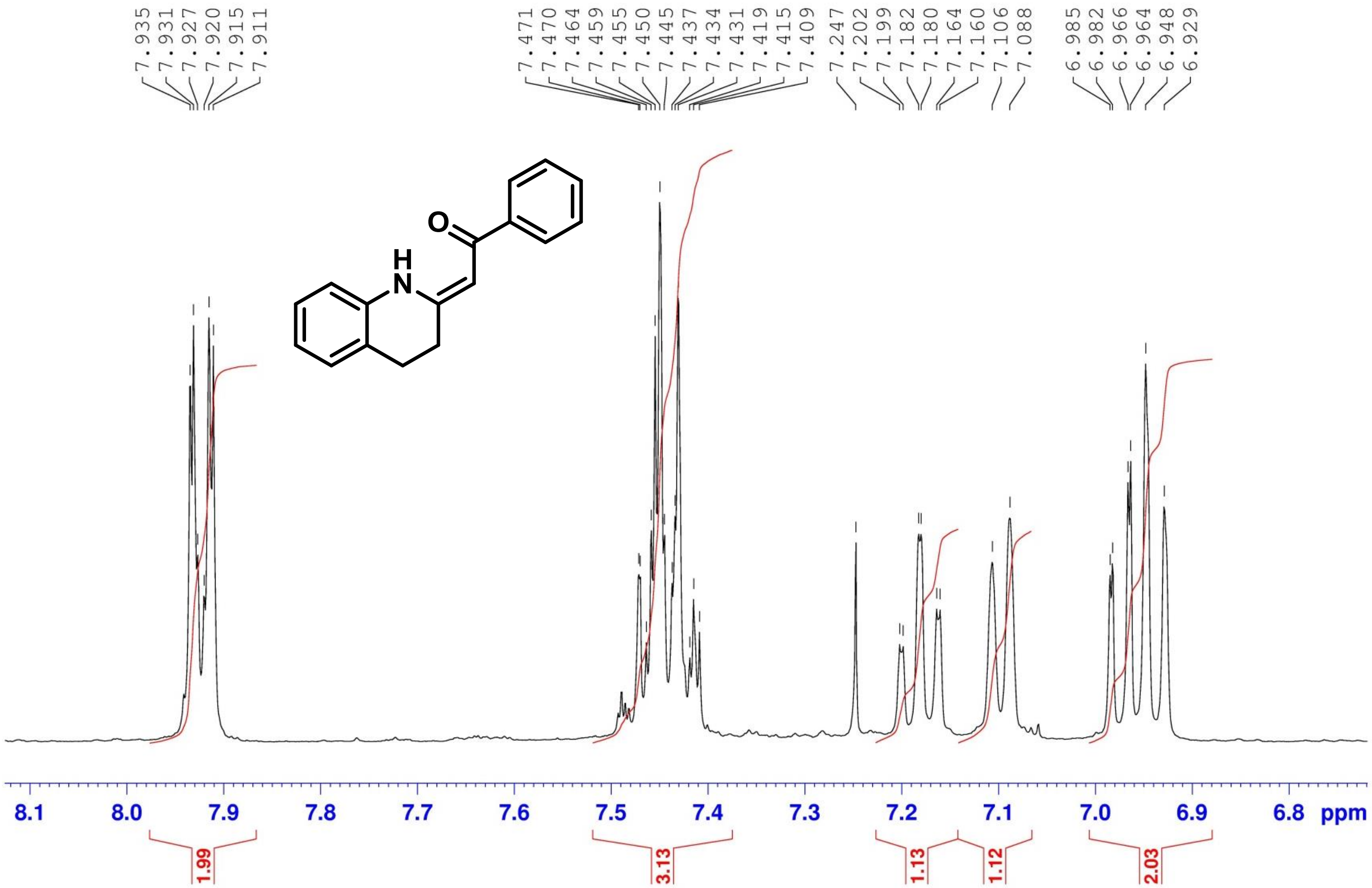

Figure S34: 100 MHz APT spectrum of the compound **1a**.

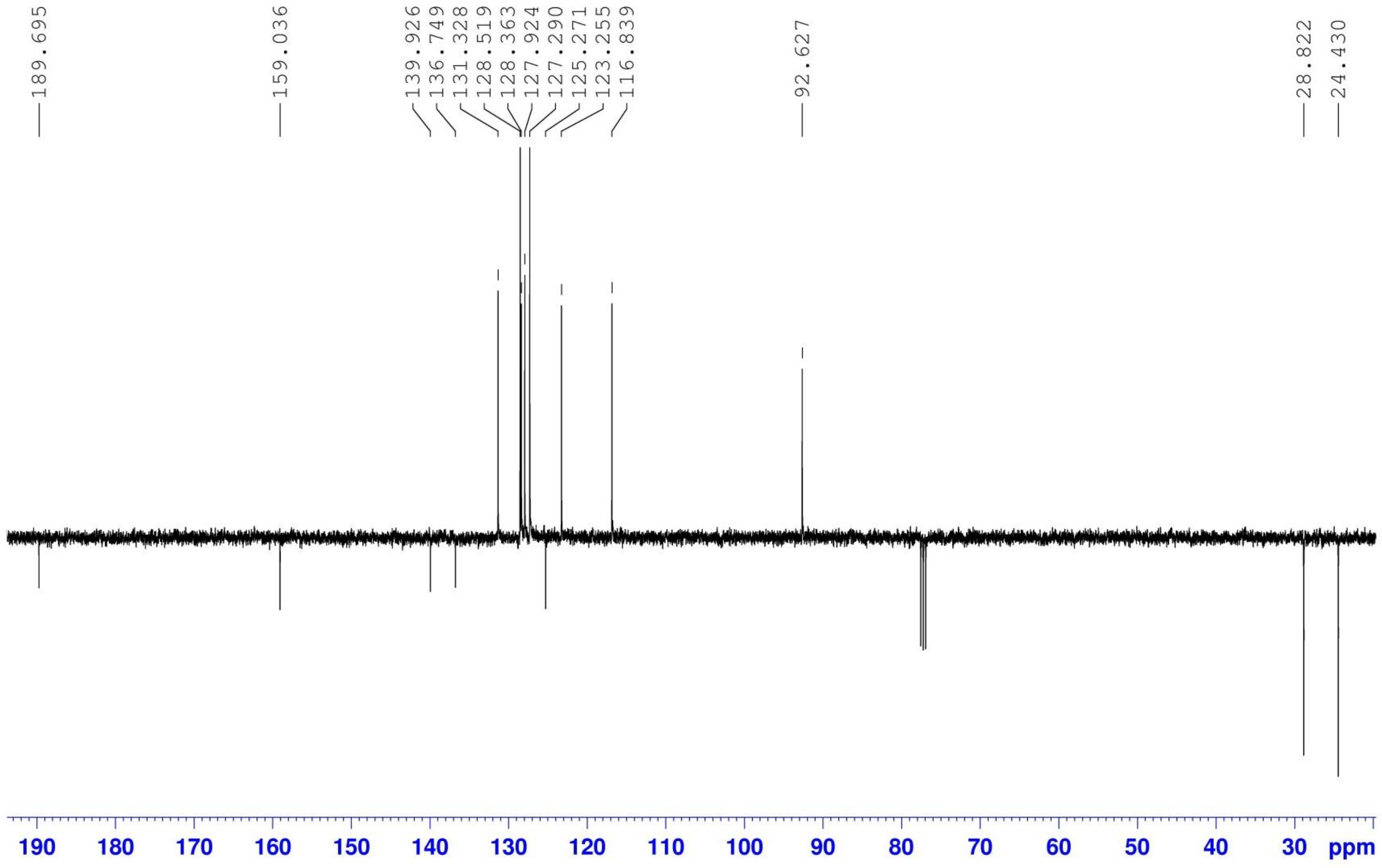

Figure S35: 400 MHz ^{1}H NMR spectrum of the compound **1b**.

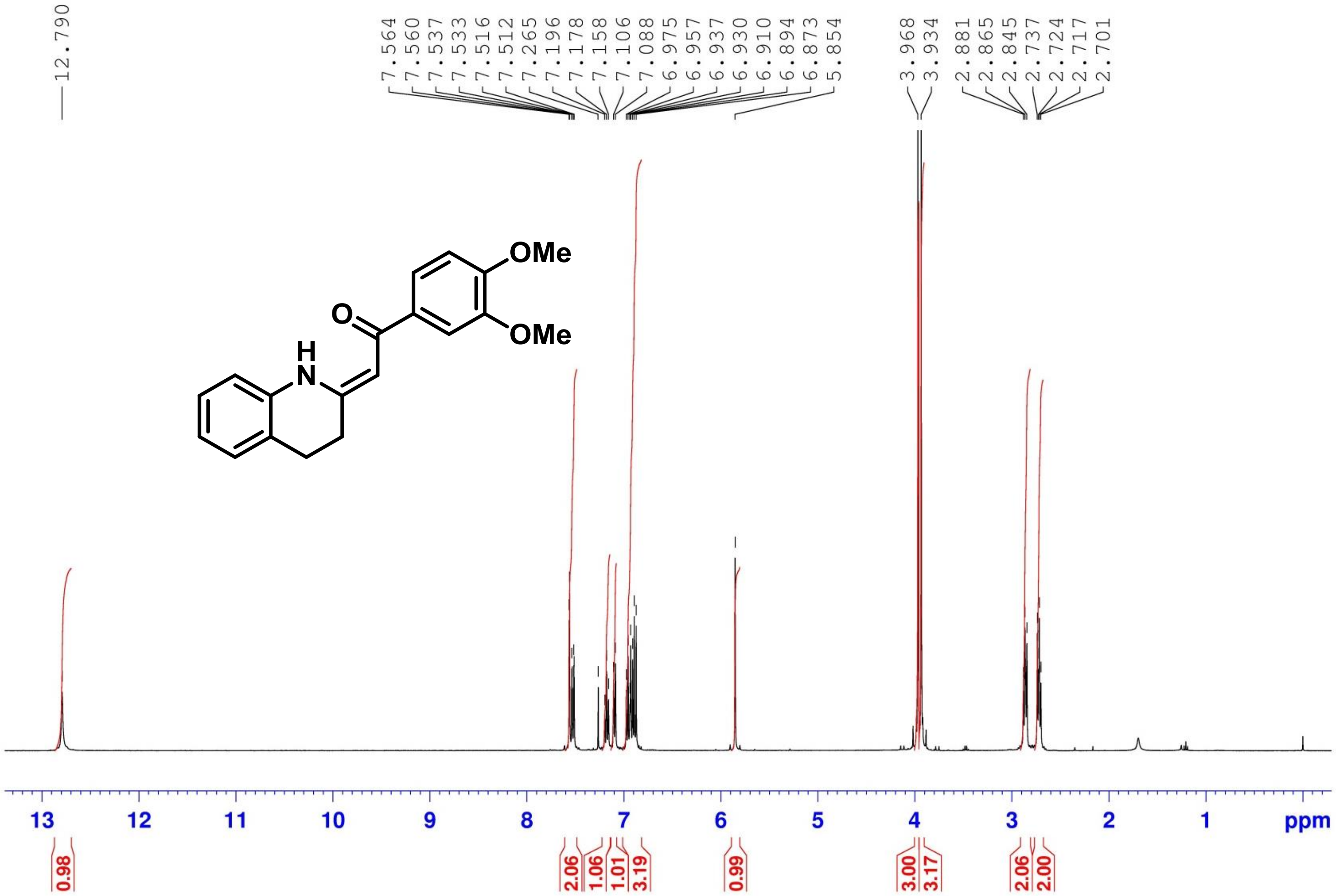

Figure S35a: 400 MHz ^{1}H NMR spectrum of the compound **1b**, a detail.

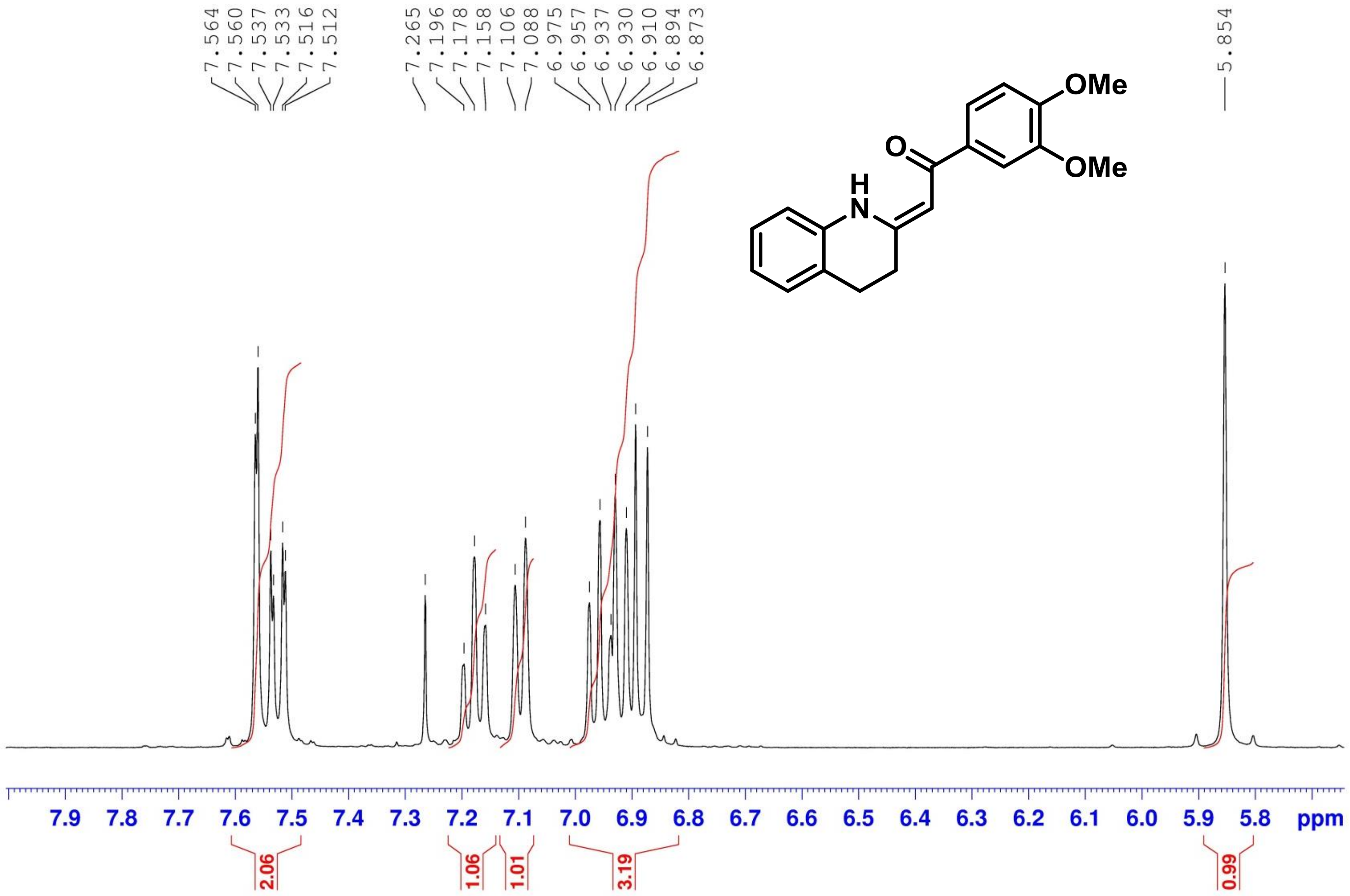

Figure S36: 100 MHz ^{13}C NMR spectrum of the compound **1b**.

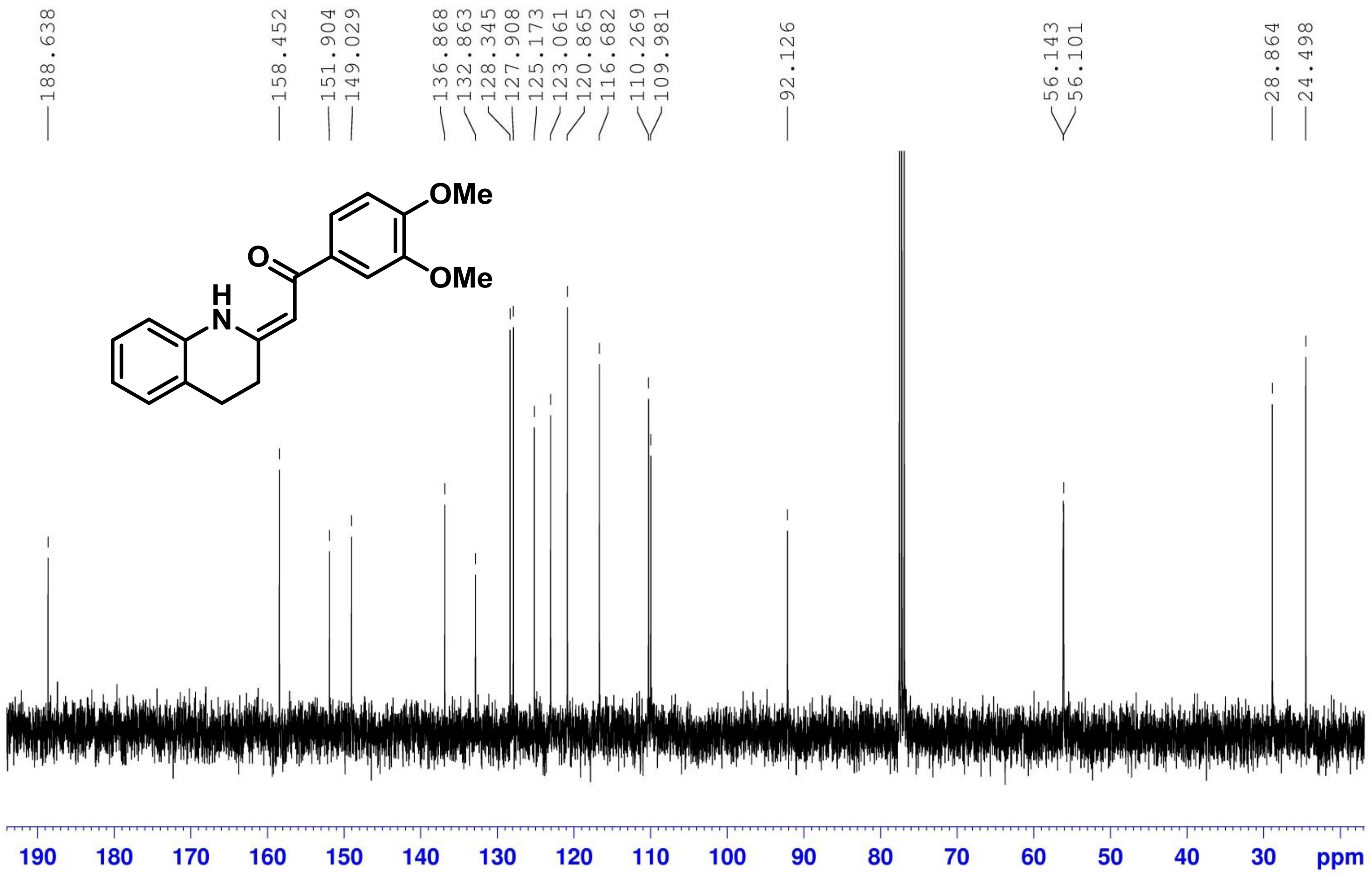

Figure S37: 500 MHz ^{1}H NMR spectrum of the compound **1c**.

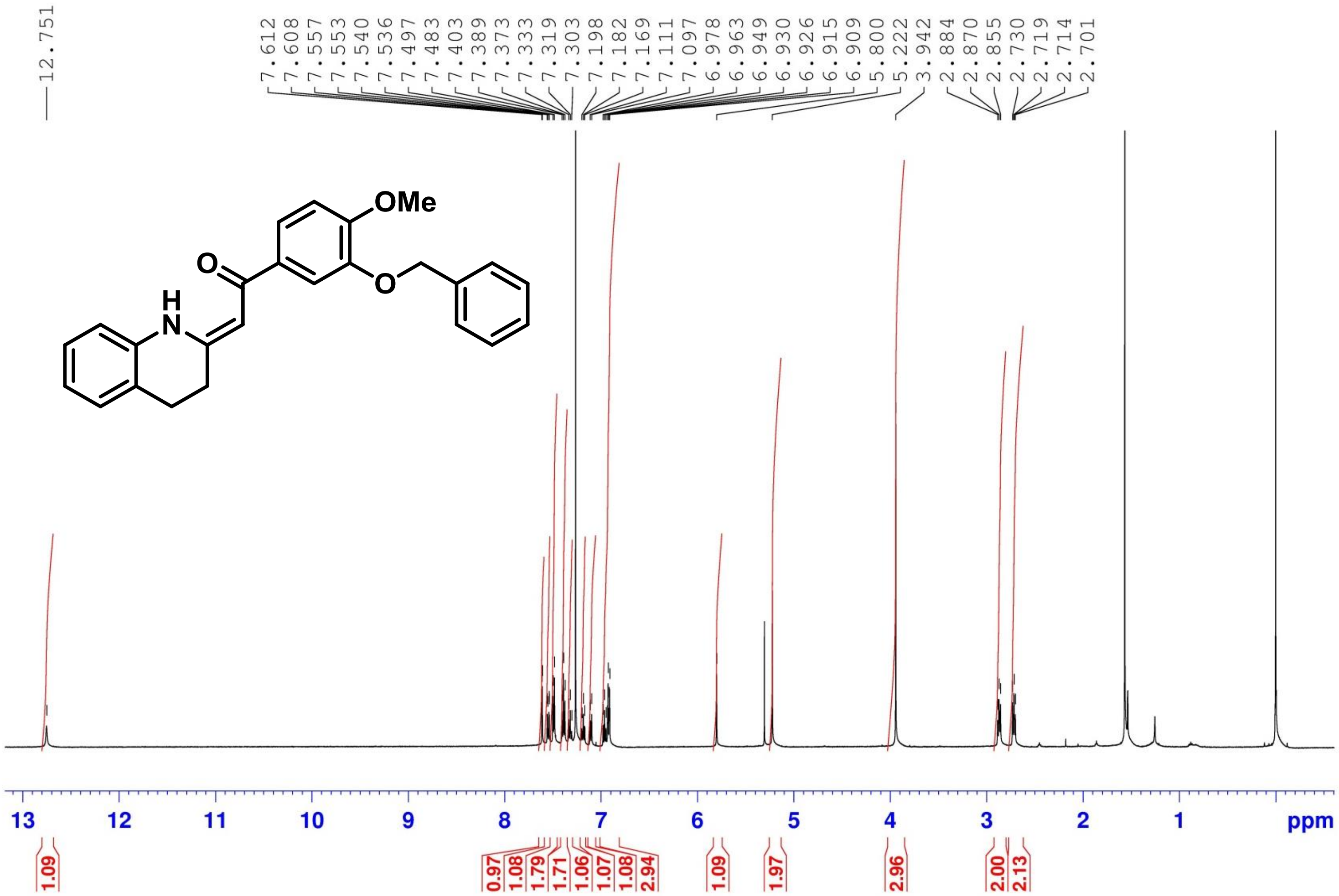

Figure S37a: 500 MHz ¹H NMR spectrum of the compound **1c**, a detail.

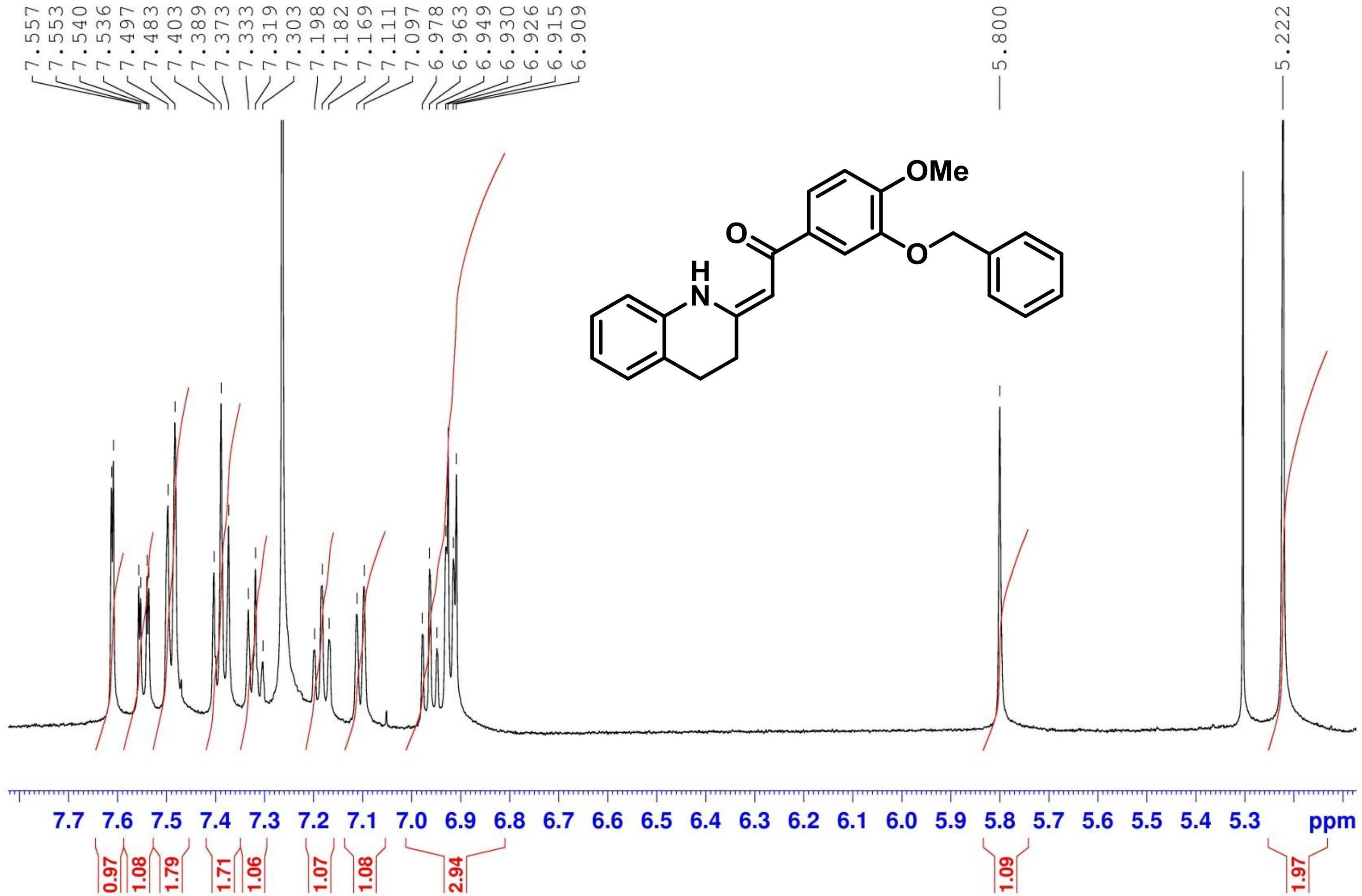

Figure S38: 125 MHz ^{13}C NMR spectrum of the compound **1c**.

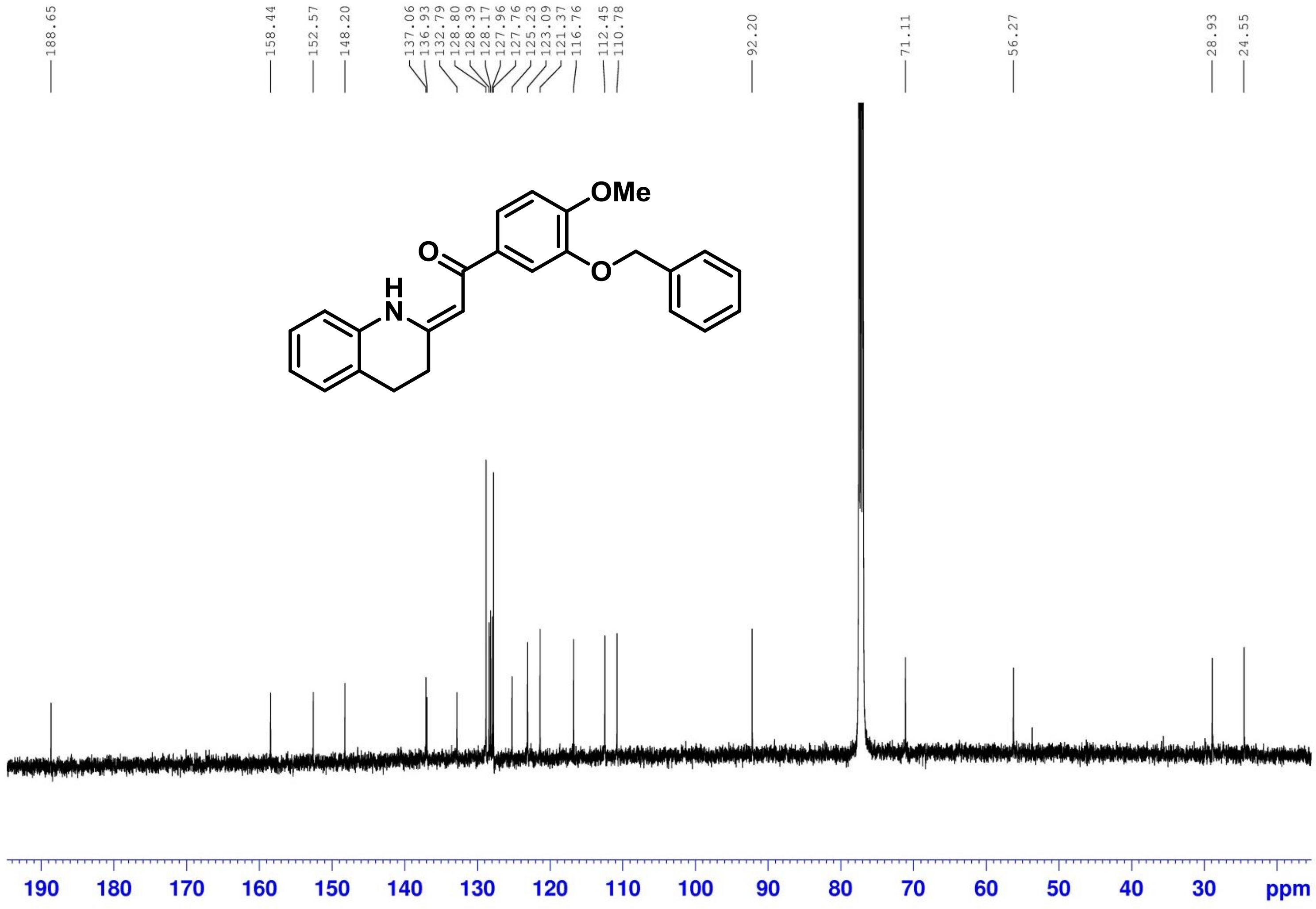

Figure S39: 400 MHz ^{1}H NMR spectrum of the compound **1d**.

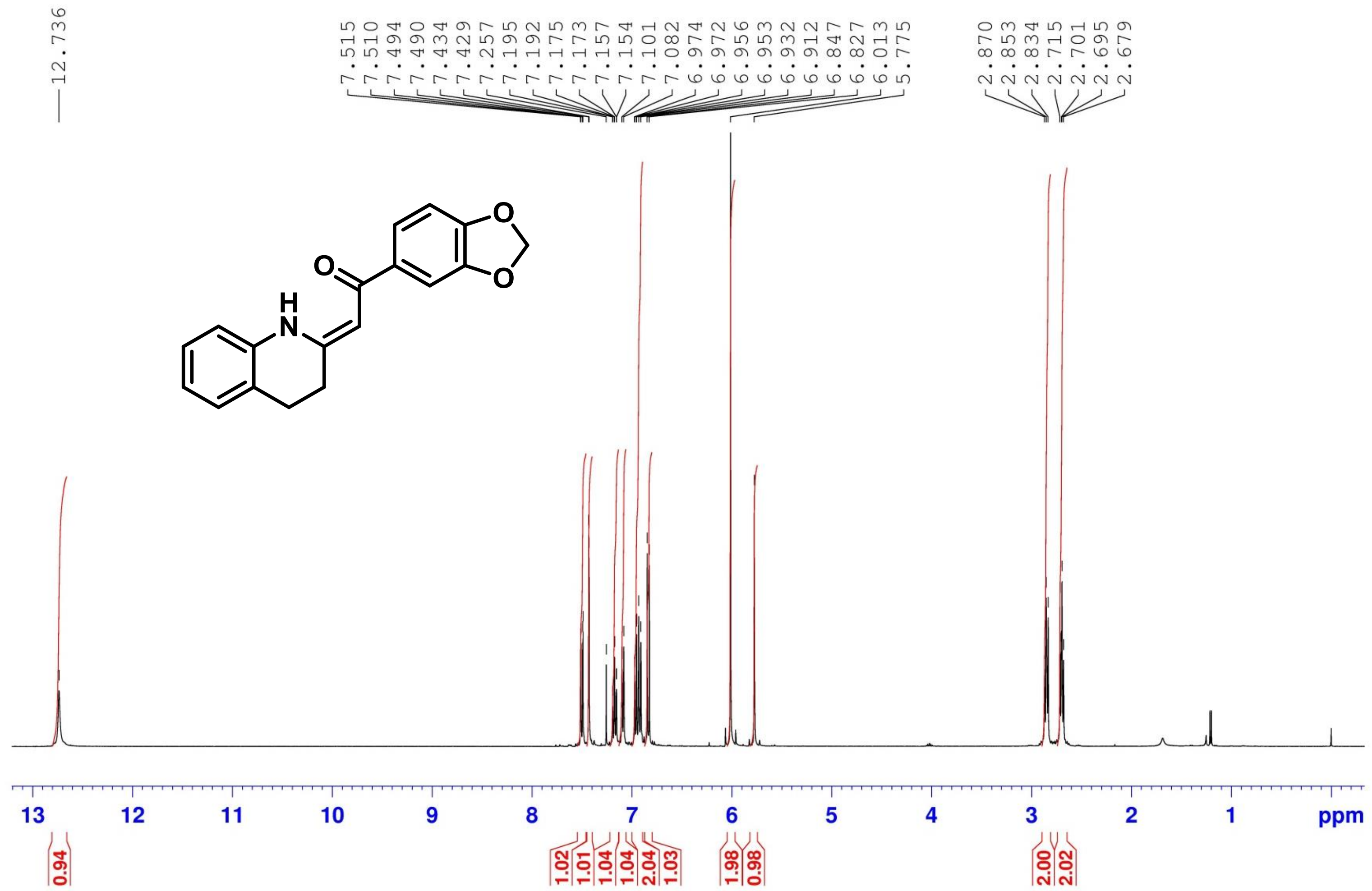

Figure S39a: 400 MHz ^{1}H NMR spectrum of the compound **1d**, a detail.

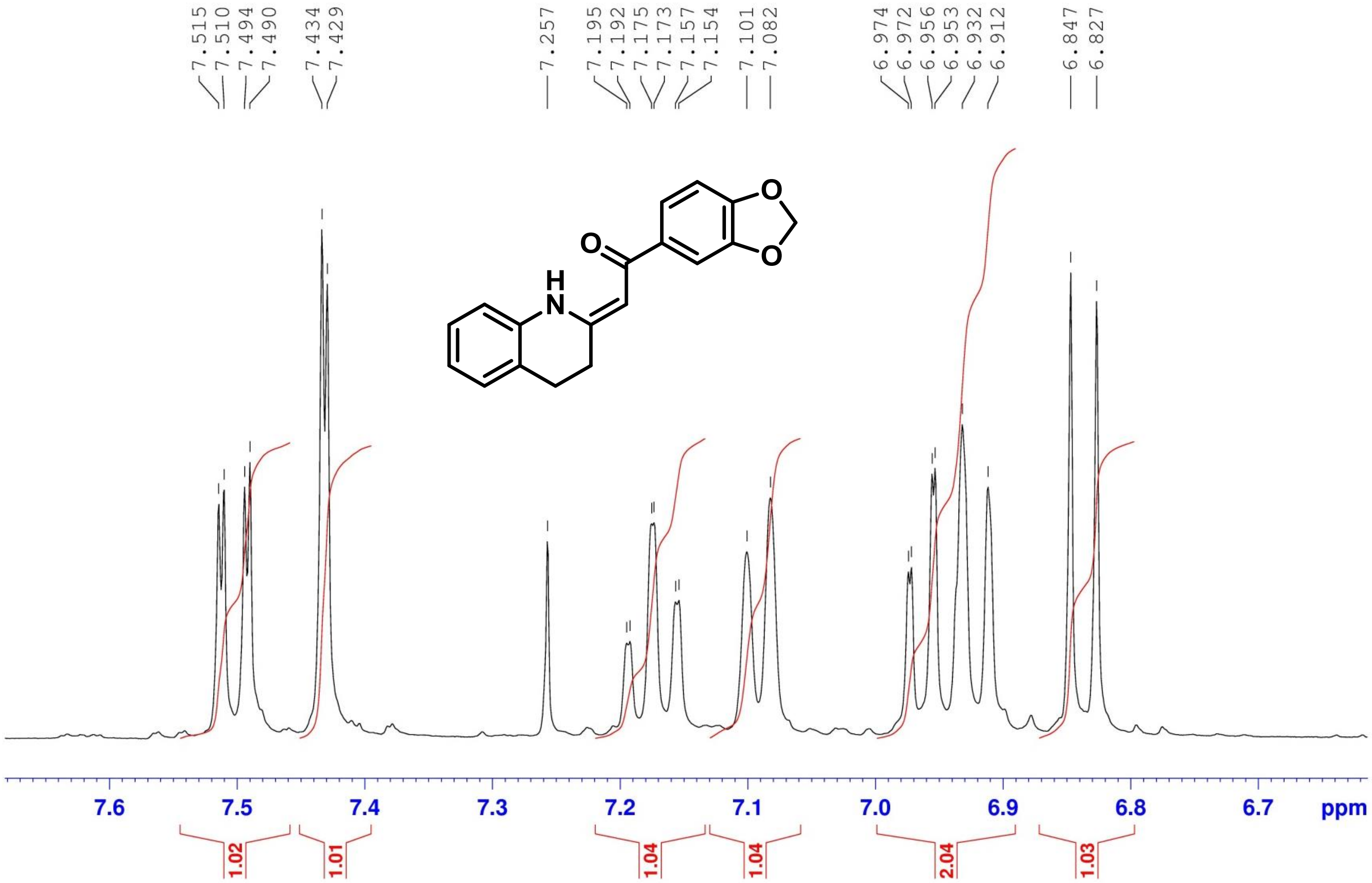

Figure S40: 100 MHz ^{1}H NMR spectrum of the compound **1d**.

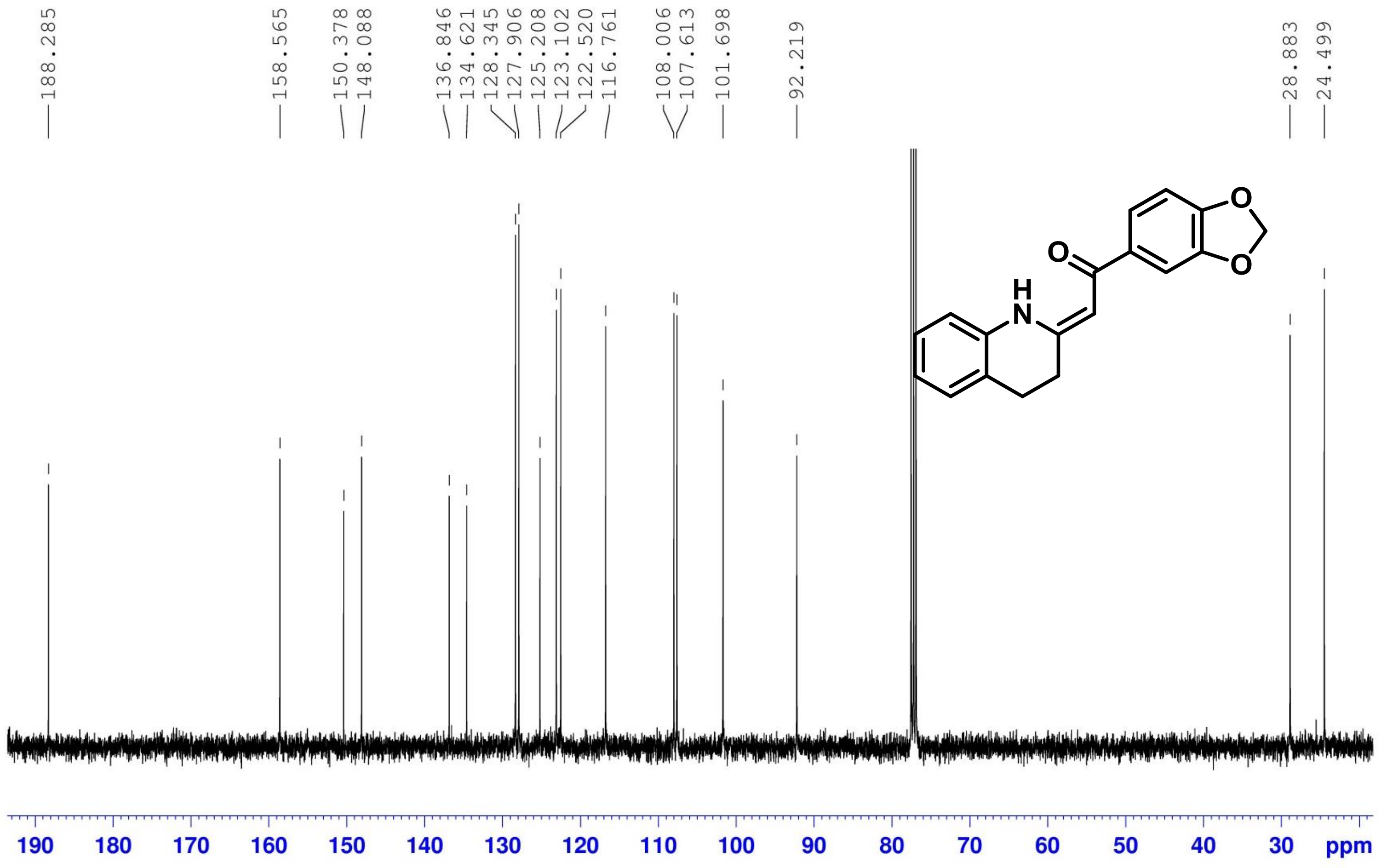

Figure S41: 400 MHz ^{1}H NMR spectrum of the compound **1e**.

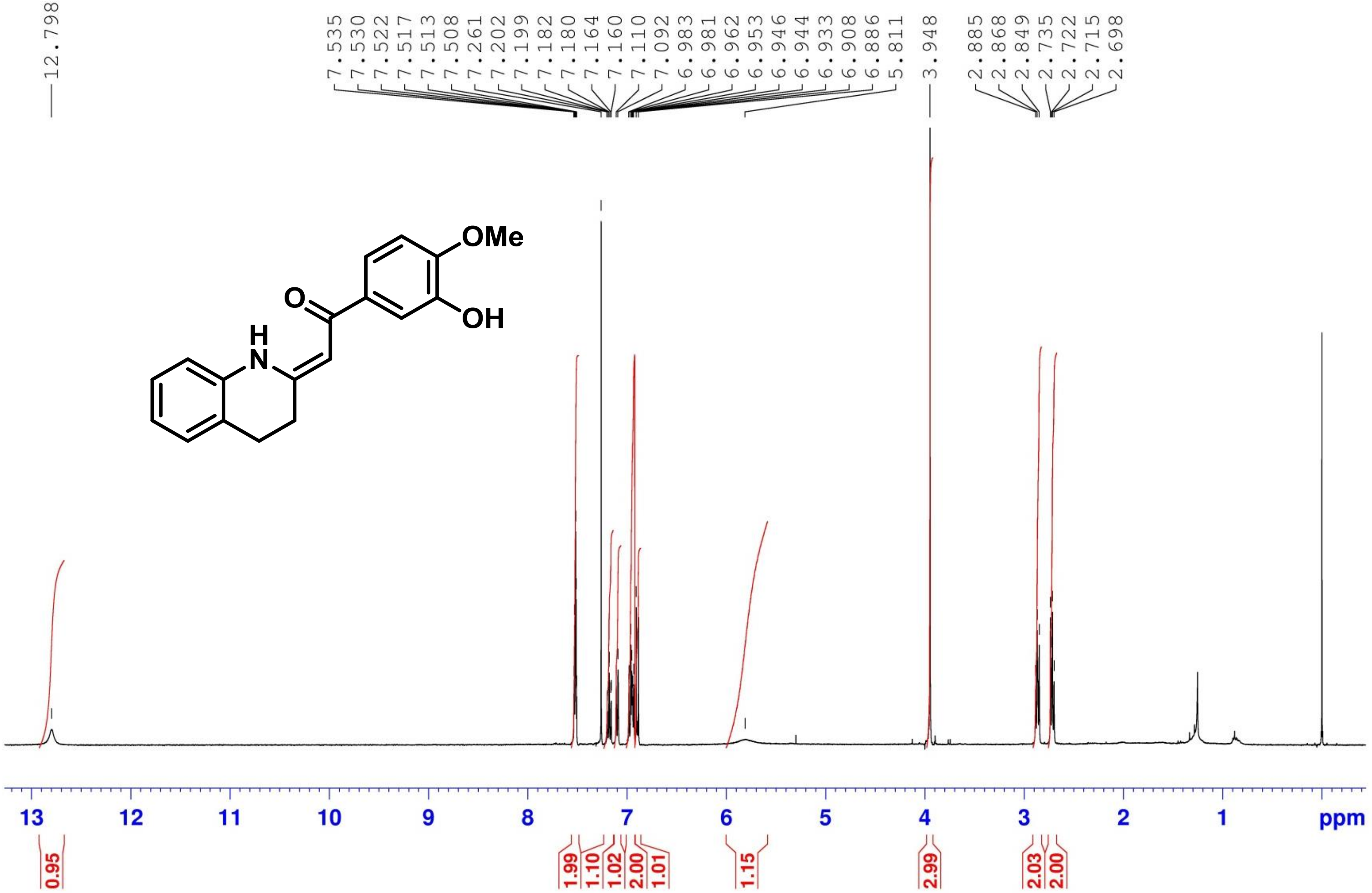

Figure S41a: 400 MHz ^{1}H NMR spectrum of the compound **1e**, a detail.

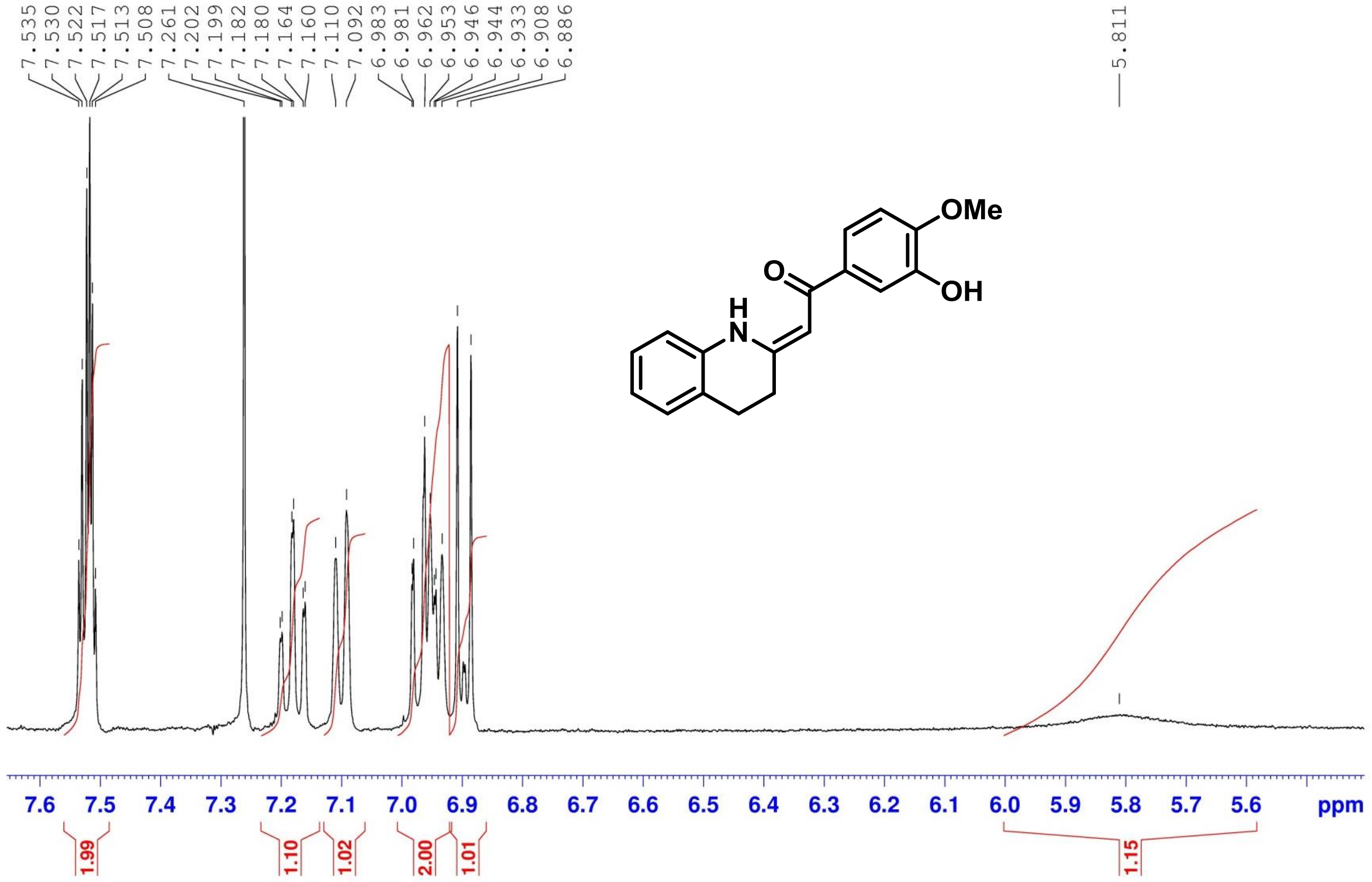

Figure S42: 100 MHz ^{13}C NMR spectrum of the compound **1e**.

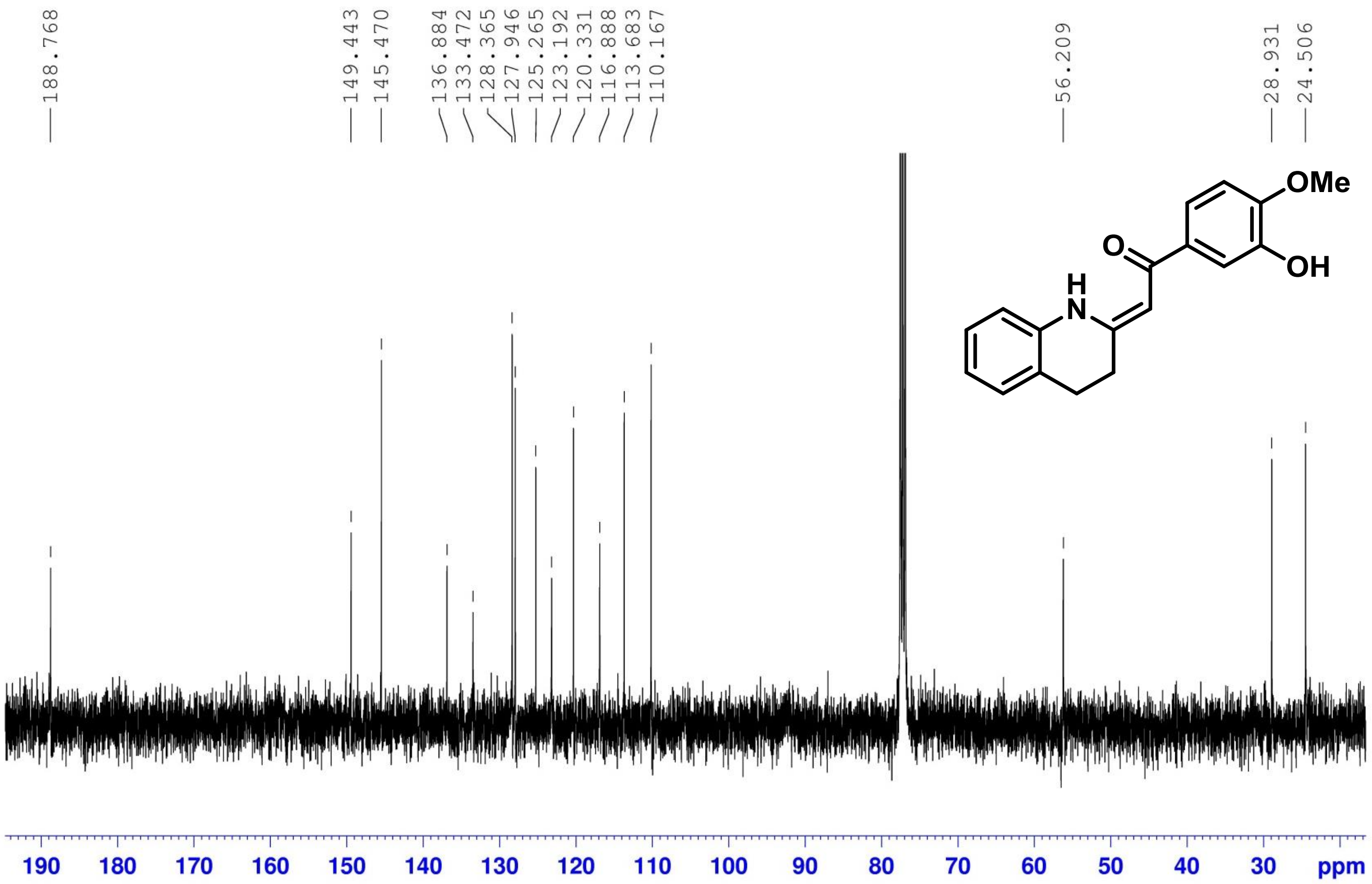

References

1. Otwinowski, Z.; Minor, W. *Macromol. Crystallography*, *Pt A*, **1997**, *276*, 307–326. doi: 10.1016/s0076-6879(97)76066-x
2. Coppens, P., Ahmed, F. R.; Hall, S. R.; Huber, C. P. *Crystallographic Computing;* Munksgaard: Copenhagen, 1970*;* pp 255–270.
3. Altomare, A., Cascarano, G.; Giacovazzo, C.; Guagliardi, A. *J. Appl. Crystall.*, **1994**, *27*, 1045–1050. doi: 10.1107/s002188989400422x
4. Sheldrick G. M. *SHELXL-97*, University of Göttingen, Göttingen, **2008**.
5. Murarka, S.; Zhang, C.; Konieczynska, M. D.; Seidel, D. *Org. Lett.* **2009**, *11*, 129–132. doi: 10.1021/ol802519r
6. Zhang, S.T.; Cheng, K.; Wang, X. H.; Yin, H. *Bioorg. Med. Chem.* **2012**, *20*, 6073–6079. doi: 10.1016/j.bmc.2012.08.022
7. Takuwa, T.; Minowa, T.; Fujisawa, H.; Mukaiyama, T. *Chem. Pharm. Bull.* **2005**, *53*, 476–480. doi: 10.1248/cpb.53.476
8. Greiner, A.C.; Spyckerelle, C.; Albrecht, P. *Tetrahedron* **1976**, *32*, 257–260. 10.1016/0040-4020(76)87011-1
9. O'Connell, J.L.; Simpson, J. S.; Dumanski, P. G.; Simpson, G. W.; Easton, C. J. *Org. Biomol. Chem.* **2006**, *4*, 2716–2723. doi: 10.1039/b605010g
10. Mennen, S.M.; Miller, S. J. *J. Org. Chem.* **2007**, *72*, 5260–5269. doi: 10.1021/jo070676d
11. Rosiak, A., Frey, W.; Christoffers, J. *Eur. J. Org. Chem.* **2006**, 4044–4054. doi: 10.1002/ejoc.200600372
12. Bose, P.; Banerji, J. *Phytochemistry* **1991**, *30*, 2438–2439. doi: 10.1016/0031-9422(91)83677-d
13. Simunek, P.; Svobodova, M.; Bertolasi, V.; Machacek, V. *Synthesis* **2008**, 1761–1766. doi: 10.1055/s-2008-1067042
14. Greenhill, J.V.; Loghmani-Khouzani, H.; Maitland, D. J. *Tetrahedron* **1988**, *44*, 3319–3326. doi: 10.1016/s0040-4020(01)85965-2
15. Wang, D.-W.; Wang, X.-B.; Wang, D.-S.; Lu, S.-M.; Zhou, Y.-G.; Li, Y.-X. *J. Org. Chem.* **2009**, *74*, 2780–2787. doi: 10.1021/jo900073z

16. Wang, X.-B.; Wang, D. W.; Lu, S.-M.; Yu, C.-B.; Yhou, Y.-G. *Tetrahedron: Asymmetry* **2009**, *20*, 1040–1045. doi: 10.1016/j.tetasy.2009.03.037
